# Performance of the ATLAS trigger system in 2015

**DOI:** 10.1140/epjc/s10052-017-4852-3

**Published:** 2017-05-18

**Authors:** Atlas Collaboration, M. Aaboud, G. Aad, B. Abbott, J. Abdallah, O. Abdinov, B. Abeloos, R. Aben, O. S. AbouZeid, N. L. Abraham, H. Abramowicz, H. Abreu, R. Abreu, Y. Abulaiti, B. S. Acharya, S. Adachi, L. Adamczyk, D. L. Adams, J. Adelman, S. Adomeit, T. Adye, A. A. Affolder, T. Agatonovic-Jovin, J. A. Aguilar-Saavedra, S. P. Ahlen, F. Ahmadov, G. Aielli, H. Akerstedt, T. P. A. Åkesson, A. V. Akimov, G. L. Alberghi, J. Albert, S. Albrand, M. J. Alconada Verzini, M. Aleksa, I. N. Aleksandrov, C. Alexa, G. Alexander, T. Alexopoulos, M. Alhroob, B. Ali, M. Aliev, G. Alimonti, J. Alison, S. P. Alkire, B. M. M. Allbrooke, B. W. Allen, P. P. Allport, A. Aloisio, A. Alonso, F. Alonso, C. Alpigiani, A. A. Alshehri, M. Alstaty, B. Alvarez Gonzalez, D. Álvarez Piqueras, M. G. Alviggi, B. T. Amadio, Y. Amaral Coutinho, C. Amelung, D. Amidei, S. P. Amor Dos Santos, A. Amorim, S. Amoroso, G. Amundsen, C. Anastopoulos, L. S. Ancu, N. Andari, T. Andeen, C. F. Anders, G. Anders, J. K. Anders, K. J. Anderson, A. Andreazza, V. Andrei, S. Angelidakis, I. Angelozzi, A. Angerami, F. Anghinolfi, A. V. Anisenkov, N. Anjos, A. Annovi, C. Antel, M. Antonelli, A. Antonov, D. J. Antrim, F. Anulli, M. Aoki, L. Aperio Bella, G. Arabidze, Y. Arai, J. P. Araque, A. T. H. Arce, F. A. Arduh, J.-F. Arguin, S. Argyropoulos, M. Arik, A. J. Armbruster, L. J. Armitage, O. Arnaez, H. Arnold, M. Arratia, O. Arslan, A. Artamonov, G. Artoni, S. Artz, S. Asai, N. Asbah, A. Ashkenazi, B. Åsman, L. Asquith, K. Assamagan, R. Astalos, M. Atkinson, N. B. Atlay, K. Augsten, G. Avolio, B. Axen, M. K. Ayoub, G. Azuelos, M. A. Baak, A. E. Baas, M. J. Baca, H. Bachacou, K. Bachas, M. Backes, M. Backhaus, P. Bagiacchi, P. Bagnaia, Y. Bai, J. T. Baines, M. Bajic, O. K. Baker, E. M. Baldin, P. Balek, T. Balestri, F. Balli, W. K. Balunas, E. Banas, Sw. Banerjee, A. A. E. Bannoura, L. Barak, E. L. Barberio, D. Barberis, M. Barbero, T. Barillari, M.-S. Barisits, T. Barklow, N. Barlow, S. L. Barnes, B. M. Barnett, R. M. Barnett, Z. Barnovska-Blenessy, A. Baroncelli, G. Barone, A. J. Barr, L. Barranco Navarro, F. Barreiro, J. Barreiro Guimarães da Costa, R. Bartoldus, A. E. Barton, P. Bartos, A. Basalaev, A. Bassalat, R. L. Bates, S. J. Batista, J. R. Batley, M. Battaglia, M. Bauce, F. Bauer, H. S. Bawa, J. B. Beacham, M. D. Beattie, T. Beau, P. H. Beauchemin, P. Bechtle, H. P. Beck, K. Becker, M. Becker, M. Beckingham, C. Becot, A. J. Beddall, A. Beddall, V. A. Bednyakov, M. Bedognetti, C. P. Bee, L. J. Beemster, T. A. Beermann, M. Begel, J. K. Behr, A. S. Bell, G. Bella, L. Bellagamba, A. Bellerive, M. Bellomo, K. Belotskiy, O. Beltramello, N. L. Belyaev, O. Benary, D. Benchekroun, M. Bender, K. Bendtz, N. Benekos, Y. Benhammou, E. Benhar Noccioli, J. Benitez, D. P. Benjamin, J. R. Bensinger, S. Bentvelsen, L. Beresford, M. Beretta, D. Berge, E. Bergeaas Kuutmann, N. Berger, J. Beringer, S. Berlendis, N. R. Bernard, C. Bernius, F. U. Bernlochner, T. Berry, P. Berta, C. Bertella, G. Bertoli, F. Bertolucci, I. A. Bertram, C. Bertsche, D. Bertsche, G. J. Besjes, O. Bessidskaia Bylund, M. Bessner, N. Besson, C. Betancourt, A. Bethani, S. Bethke, A. J. Bevan, R. M. Bianchi, M. Bianco, O. Biebel, D. Biedermann, R. Bielski, N. V. Biesuz, M. Biglietti, J. Bilbao De Mendizabal, T. R. V. Billoud, H. Bilokon, M. Bindi, A. Bingul, C. Bini, S. Biondi, T. Bisanz, D. M. Bjergaard, C. W. Black, J. E. Black, K. M. Black, D. Blackburn, R. E. Blair, T. Blazek, I. Bloch, C. Blocker, A. Blue, W. Blum, U. Blumenschein, S. Blunier, G. J. Bobbink, V. S. Bobrovnikov, S. S. Bocchetta, A. Bocci, C. Bock, M. Boehler, D. Boerner, J. A. Bogaerts, D. Bogavac, A. G. Bogdanchikov, C. Bohm, V. Boisvert, P. Bokan, T. Bold, A. S. Boldyrev, M. Bomben, M. Bona, M. Boonekamp, A. Borisov, G. Borissov, J. Bortfeldt, D. Bortoletto, V. Bortolotto, K. Bos, D. Boscherini, M. Bosman, J. D. Bossio Sola, J. Boudreau, J. Bouffard, E. V. Bouhova-Thacker, D. Boumediene, C. Bourdarios, S. K. Boutle, A. Boveia, J. Boyd, I. R. Boyko, J. Bracinik, A. Brandt, G. Brandt, O. Brandt, U. Bratzler, B. Brau, J. E. Brau, W. D. Breaden Madden, K. Brendlinger, A. J. Brennan, L. Brenner, R. Brenner, S. Bressler, T. M. Bristow, D. Britton, D. Britzger, F. M. Brochu, I. Brock, R. Brock, G. Brooijmans, T. Brooks, W. K. Brooks, J. Brosamer, E. Brost, J. H Broughton, P. A. Bruckman de Renstrom, D. Bruncko, R. Bruneliere, A. Bruni, G. Bruni, L. S. Bruni, BH Brunt, M. Bruschi, N. Bruscino, P. Bryant, L. Bryngemark, T. Buanes, Q. Buat, P. Buchholz, A. G. Buckley, I. A. Budagov, F. Buehrer, M. K. Bugge, O. Bulekov, D. Bullock, H. Burckhart, S. Burdin, C. D. Burgard, A. M. Burger, B. Burghgrave, K. Burka, S. Burke, I. Burmeister, J. T. P. Burr, E. Busato, D. Büscher, V. Büscher, P. Bussey, J. M. Butler, C. M. Buttar, J. M. Butterworth, P. Butti, W. Buttinger, A. Buzatu, A. R. Buzykaev, S. Cabrera Urbán, D. Caforio, V. M. Cairo, O. Cakir, N. Calace, P. Calafiura, A. Calandri, G. Calderini, P. Calfayan, G. Callea, L. P. Caloba, S. Calvente Lopez, D. Calvet, S. Calvet, T. P. Calvet, R. Camacho Toro, S. Camarda, P. Camarri, D. Cameron, R. Caminal Armadans, C. Camincher, S. Campana, M. Campanelli, A. Camplani, A. Campoverde, V. Canale, A. Canepa, M. Cano Bret, J. Cantero, T. Cao, M. D. M. Capeans Garrido, I. Caprini, M. Caprini, M. Capua, R. M. Carbone, R. Cardarelli, F. Cardillo, I. Carli, T. Carli, G. Carlino, B. T. Carlson, L. Carminati, R. M. D. Carney, S. Caron, E. Carquin, G. D. Carrillo-Montoya, J. R. Carter, J. Carvalho, D. Casadei, M. P. Casado, M. Casolino, D. W. Casper, E. Castaneda-Miranda, R. Castelijn, A. Castelli, V. Castillo Gimenez, N. F. Castro, A. Catinaccio, J. R. Catmore, A. Cattai, J. Caudron, V. Cavaliere, E. Cavallaro, D. Cavalli, M. Cavalli-Sforza, V. Cavasinni, F. Ceradini, L. Cerda Alberich, A. S. Cerqueira, A. Cerri, L. Cerrito, F. Cerutti, A. Cervelli, S. A. Cetin, A. Chafaq, D. Chakraborty, S. K. Chan, Y. L. Chan, P. Chang, J. D. Chapman, D. G. Charlton, A. Chatterjee, C. C. Chau, C. A. Chavez Barajas, S. Che, S. Cheatham, A. Chegwidden, S. Chekanov, S. V. Chekulaev, G. A. Chelkov, M. A. Chelstowska, C. Chen, H. Chen, K. Chen, S. Chen, S. Chen, X. Chen, Y. Chen, H. C. Cheng, H. J. Cheng, Y. Cheng, A. Cheplakov, E. Cheremushkina, R. Cherkaoui El Moursli, V. Chernyatin, E. Cheu, L. Chevalier, V. Chiarella, G. Chiarelli, G. Chiodini, A. S. Chisholm, A. Chitan, M. V. Chizhov, K. Choi, A. R. Chomont, S. Chouridou, B. K. B. Chow, V. Christodoulou, D. Chromek-Burckhart, J. Chudoba, A. J. Chuinard, J. J. Chwastowski, L. Chytka, G. Ciapetti, A. K. Ciftci, D. Cinca, V. Cindro, I. A. Cioara, C. Ciocca, A. Ciocio, F. Cirotto, Z. H. Citron, M. Citterio, M. Ciubancan, A. Clark, B. L. Clark, M. R. Clark, P. J. Clark, R. N. Clarke, C. Clement, Y. Coadou, M. Cobal, A. Coccaro, J. Cochran, L. Colasurdo, B. Cole, A. P. Colijn, J. Collot, T. Colombo, G. Compostella, P. Conde Muiño, E. Coniavitis, S. H. Connell, I. A. Connelly, V. Consorti, S. Constantinescu, G. Conti, F. Conventi, M. Cooke, B. D. Cooper, A. M. Cooper-Sarkar, F. Cormier, K. J. R. Cormier, T. Cornelissen, M. Corradi, F. Corriveau, A. Cortes-Gonzalez, G. Cortiana, G. Costa, M. J. Costa, D. Costanzo, G. Cottin, G. Cowan, B. E. Cox, K. Cranmer, S. J. Crawley, G. Cree, S. Crépé-Renaudin, F. Crescioli, W. A. Cribbs, M. Crispin Ortuzar, M. Cristinziani, V. Croft, G. Crosetti, A. Cueto, T. Cuhadar Donszelmann, J. Cummings, M. Curatolo, J. Cúth, H. Czirr, P. Czodrowski, G. D’amen, S. D’Auria, M. D’Onofrio, M. J. Da Cunha Sargedas De Sousa, C. Da Via, W. Dabrowski, T. Dado, T. Dai, O. Dale, F. Dallaire, C. Dallapiccola, M. Dam, J. R. Dandoy, N. P. Dang, A. C. Daniells, N. S. Dann, M. Danninger, M. Dano Hoffmann, V. Dao, G. Darbo, S. Darmora, J. Dassoulas, A. Dattagupta, W. Davey, C. David, T. Davidek, M. Davies, P. Davison, E. Dawe, I. Dawson, K. De, R. de Asmundis, A. De Benedetti, S. De Castro, S. De Cecco, N. De Groot, P. de Jong, H. De la Torre, F. De Lorenzi, A. De Maria, D. De Pedis, A. De Salvo, U. De Sanctis, A. De Santo, J. B. De Vivie De Regie, W. J. Dearnaley, R. Debbe, C. Debenedetti, D. V. Dedovich, N. Dehghanian, I. Deigaard, M. Del Gaudio, J. Del Peso, T. Del Prete, D. Delgove, F. Deliot, C. M. Delitzsch, A. Dell’Acqua, L. Dell’Asta, M. Dell’Orso, M. Della Pietra, D. della Volpe, M. Delmastro, P. A. Delsart, D. A. DeMarco, S. Demers, M. Demichev, A. Demilly, S. P. Denisov, D. Denysiuk, D. Derendarz, J. E. Derkaoui, F. Derue, P. Dervan, K. Desch, C. Deterre, K. Dette, P. O. Deviveiros, A. Dewhurst, S. Dhaliwal, A. Di Ciaccio, L. Di Ciaccio, W. K. Di Clemente, C. Di Donato, A. Di Girolamo, B. Di Girolamo, B. Di Micco, R. Di Nardo, A. Di Simone, R. Di Sipio, D. Di Valentino, C. Diaconu, M. Diamond, F. A. Dias, M. A. Diaz, E. B. Diehl, J. Dietrich, S. Díez Cornell, A. Dimitrievska, J. Dingfelder, P. Dita, S. Dita, F. Dittus, F. Djama, T. Djobava, J. I. Djuvsland, M. A. B. do Vale, D. Dobos, M. Dobre, C. Doglioni, J. Dolejsi, Z. Dolezal, M. Donadelli, S. Donati, P. Dondero, J. Donini, J. Dopke, A. Doria, M. T. Dova, A. T. Doyle, E. Drechsler, M. Dris, Y. Du, J. Duarte-Campderros, E. Duchovni, G. Duckeck, O. A. Ducu, D. Duda, A. Dudarev, A. Chr. Dudder, E. M. Duffield, L. Duflot, M. Dührssen, M. Dumancic, A. K. Duncan, M. Dunford, H. Duran Yildiz, M. Düren, A. Durglishvili, D. Duschinger, B. Dutta, M. Dyndal, C. Eckardt, K. M. Ecker, R. C. Edgar, N. C. Edwards, T. Eifert, G. Eigen, K. Einsweiler, T. Ekelof, M. El Kacimi, V. Ellajosyula, M. Ellert, S. Elles, F. Ellinghaus, A. A. Elliot, N. Ellis, J. Elmsheuser, M. Elsing, D. Emeliyanov, Y. Enari, O. C. Endner, J. S. Ennis, J. Erdmann, A. Ereditato, G. Ernis, J. Ernst, M. Ernst, S. Errede, E. Ertel, M. Escalier, H. Esch, C. Escobar, B. Esposito, A. I. Etienvre, E. Etzion, H. Evans, A. Ezhilov, M. Ezzi, F. Fabbri, L. Fabbri, G. Facini, R. M. Fakhrutdinov, S. Falciano, R. J. Falla, J. Faltova, Y. Fang, M. Fanti, A. Farbin, A. Farilla, C. Farina, E. M. Farina, T. Farooque, S. Farrell, S. M. Farrington, P. Farthouat, F. Fassi, P. Fassnacht, D. Fassouliotis, M. Faucci Giannelli, A. Favareto, W. J. Fawcett, L. Fayard, O. L. Fedin, W. Fedorko, S. Feigl, L. Feligioni, C. Feng, E. J. Feng, H. Feng, A. B. Fenyuk, L. Feremenga, P. Fernandez Martinez, S. Fernandez Perez, J. Ferrando, A. Ferrari, P. Ferrari, R. Ferrari, D. E. Ferreira de Lima, A. Ferrer, D. Ferrere, C. Ferretti, F. Fiedler, A. Filipčič, M. Filipuzzi, F. Filthaut, M. Fincke-Keeler, K. D. Finelli, M. C. N. Fiolhais, L. Fiorini, A. Fischer, C. Fischer, J. Fischer, W. C. Fisher, N. Flaschel, I. Fleck, P. Fleischmann, G. T. Fletcher, R. R. M. Fletcher, T. Flick, B. M. Flierl, L. R. Flores Castillo, M. J. Flowerdew, G. T. Forcolin, A. Formica, A. Forti, A. G. Foster, D. Fournier, H. Fox, S. Fracchia, P. Francavilla, M. Franchini, D. Francis, L. Franconi, M. Franklin, M. Frate, M. Fraternali, D. Freeborn, S. M. Fressard-Batraneanu, F. Friedrich, D. Froidevaux, J. A. Frost, C. Fukunaga, E. Fullana Torregrosa, T. Fusayasu, J. Fuster, C. Gabaldon, O. Gabizon, A. Gabrielli, A. Gabrielli, G. P. Gach, S. Gadatsch, G. Gagliardi, L. G. Gagnon, P. Gagnon, C. Galea, B. Galhardo, E. J. Gallas, B. J. Gallop, P. Gallus, G. Galster, K. K. Gan, S. Ganguly, J. Gao, Y. Gao, Y. S. Gao, F. M. Garay Walls, C. García, J. E. García Navarro, M. Garcia-Sciveres, R. W. Gardner, N. Garelli, V. Garonne, A. Gascon Bravo, K. Gasnikova, C. Gatti, A. Gaudiello, G. Gaudio, L. Gauthier, I. L. Gavrilenko, C. Gay, G. Gaycken, E. N. Gazis, Z. Gecse, C. N. P. Gee, Ch. Geich-Gimbel, M. Geisen, M. P. Geisler, K. Gellerstedt, C. Gemme, M. H. Genest, C. Geng, S. Gentile, C. Gentsos, S. George, D. Gerbaudo, A. Gershon, S. Ghasemi, M. Ghneimat, B. Giacobbe, S. Giagu, P. Giannetti, S. M. Gibson, M. Gignac, M. Gilchriese, T. P. S. Gillam, D. Gillberg, G. Gilles, D. M. Gingrich, N. Giokaris, M. P. Giordani, F. M. Giorgi, P. F. Giraud, P. Giromini, D. Giugni, F. Giuli, C. Giuliani, M. Giulini, B. K. Gjelsten, S. Gkaitatzis, I. Gkialas, E. L. Gkougkousis, L. K. Gladilin, C. Glasman, J. Glatzer, P. C. F. Glaysher, A. Glazov, M. Goblirsch-Kolb, J. Godlewski, S. Goldfarb, T. Golling, D. Golubkov, A. Gomes, R. Gonçalo, J. Goncalves Pinto Firmino Da Costa, G. Gonella, L. Gonella, A. Gongadze, S. González de la Hoz, S. Gonzalez-Sevilla, L. Goossens, P. A. Gorbounov, H. A. Gordon, I. Gorelov, B. Gorini, E. Gorini, A. Gorišek, E. Gornicki, A. T. Goshaw, C. Gössling, M. I. Gostkin, C. R. Goudet, D. Goujdami, A. G. Goussiou, N. Govender, E. Gozani, L. Graber, I. Grabowska-Bold, P. O. J. Gradin, P. Grafström, J. Gramling, E. Gramstad, S. Grancagnolo, V. Gratchev, P. M. Gravila, H. M. Gray, E. Graziani, Z. D. Greenwood, C. Grefe, K. Gregersen, I. M. Gregor, P. Grenier, K. Grevtsov, J. Griffiths, A. A. Grillo, K. Grimm, S. Grinstein, Ph. Gris, J.-F. Grivaz, S. Groh, E. Gross, J. Grosse-Knetter, G. C. Grossi, Z. J. Grout, L. Guan, W. Guan, J. Guenther, F. Guescini, D. Guest, O. Gueta, B. Gui, E. Guido, T. Guillemin, S. Guindon, U. Gul, C. Gumpert, J. Guo, Y. Guo, R. Gupta, S. Gupta, G. Gustavino, P. Gutierrez, N. G. Gutierrez Ortiz, C. Gutschow, C. Guyot, C. Gwenlan, C. B. Gwilliam, A. Haas, C. Haber, H. K. Hadavand, N. Haddad, A. Hadef, S. Hageböck, M. Hagihara, Z. Hajduk, H. Hakobyan, M. Haleem, J. Haley, G. Halladjian, G. D. Hallewell, K. Hamacher, P. Hamal, K. Hamano, A. Hamilton, G. N. Hamity, P. G. Hamnett, L. Han, K. Hanagaki, K. Hanawa, M. Hance, B. Haney, P. Hanke, R. Hanna, J. B. Hansen, J. D. Hansen, M. C. Hansen, P. H. Hansen, K. Hara, A. S. Hard, T. Harenberg, F. Hariri, S. Harkusha, R. D. Harrington, P. F. Harrison, F. Hartjes, N. M. Hartmann, M. Hasegawa, Y. Hasegawa, A. Hasib, S. Hassani, S. Haug, R. Hauser, L. Hauswald, M. Havranek, C. M. Hawkes, R. J. Hawkings, D. Hayakawa, D. Hayden, C. P. Hays, J. M. Hays, H. S. Hayward, S. J. Haywood, S. J. Head, T. Heck, V. Hedberg, L. Heelan, S. Heim, T. Heim, B. Heinemann, J. J. Heinrich, L. Heinrich, C. Heinz, J. Hejbal, L. Helary, S. Hellman, C. Helsens, J. Henderson, R. C. W. Henderson, Y. Heng, S. Henkelmann, A. M. Henriques Correia, S. Henrot-Versille, G. H. Herbert, H. Herde, V. Herget, Y. Hernández Jiménez, G. Herten, R. Hertenberger, L. Hervas, G. G. Hesketh, N. P. Hessey, J. W. Hetherly, E. Higón-Rodriguez, E. Hill, J. C. Hill, K. H. Hiller, S. J. Hillier, I. Hinchliffe, E. Hines, M. Hirose, D. Hirschbuehl, X. Hoad, J. Hobbs, N. Hod, M. C. Hodgkinson, P. Hodgson, A. Hoecker, M. R. Hoeferkamp, F. Hoenig, D. Hohn, T. R. Holmes, M. Homann, T. Honda, T. M. Hong, B. H. Hooberman, W. H. Hopkins, Y. Horii, A. J. Horton, J.-Y. Hostachy, S. Hou, A. Hoummada, J. Howarth, J. Hoya, M. Hrabovsky, I. Hristova, J. Hrivnac, T. Hryn’ova, A. Hrynevich, P. J. Hsu, S.-C. Hsu, Q. Hu, S. Hu, Y. Huang, Z. Hubacek, F. Hubaut, F. Huegging, T. B. Huffman, E. W. Hughes, G. Hughes, M. Huhtinen, P. Huo, N. Huseynov, J. Huston, J. Huth, G. Iacobucci, G. Iakovidis, I. Ibragimov, L. Iconomidou-Fayard, E. Ideal, Z. Idrissi, P. Iengo, O. Igonkina, T. Iizawa, T. Ikai, Y. Ikegami, M. Ikeno, Y. Ilchenko, D. Iliadis, N. Ilic, G. Introzzi, P. Ioannou, M. Iodice, K. Iordanidou, V. Ippolito, N. Ishijima, M. Ishino, M. Ishitsuka, R. Ishmukhametov, C. Issever, S. Istin, F. Ito, J. M. Iturbe Ponce, R. Iuppa, W. Iwanski, H. Iwasaki, J. M. Izen, V. Izzo, S. Jabbar, B. Jackson, P. Jackson, V. Jain, K. B. Jakobi, K. Jakobs, S. Jakobsen, T. Jakoubek, D. O. Jamin, D. K. Jana, R. Jansky, J. Janssen, M. Janus, P. A. Janus, G. Jarlskog, N. Javadov, T. Javůrek, F. Jeanneau, L. Jeanty, J. Jejelava, G.-Y. Jeng, D. Jennens, P. Jenni, C. Jeske, S. Jézéquel, H. Ji, J. Jia, H. Jiang, Y. Jiang, Z. Jiang, S. Jiggins, J. Jimenez Pena, S. Jin, A. Jinaru, O. Jinnouchi, H. Jivan, P. Johansson, K. A. Johns, W. J. Johnson, K. Jon-And, G. Jones, R. W. L. Jones, S. Jones, T. J. Jones, J. Jongmanns, P. M. Jorge, J. Jovicevic, X. Ju, A. Juste Rozas, M. K. Köhler, A. Kaczmarska, M. Kado, H. Kagan, M. Kagan, S. J. Kahn, T. Kaji, E. Kajomovitz, C. W. Kalderon, A. Kaluza, S. Kama, A. Kamenshchikov, N. Kanaya, S. Kaneti, L. Kanjir, V. A. Kantserov, J. Kanzaki, B. Kaplan, L. S. Kaplan, A. Kapliy, D. Kar, K. Karakostas, A. Karamaoun, N. Karastathis, M. J. Kareem, E. Karentzos, M. Karnevskiy, S. N. Karpov, Z. M. Karpova, K. Karthik, V. Kartvelishvili, A. N. Karyukhin, K. Kasahara, L. Kashif, R. D. Kass, A. Kastanas, Y. Kataoka, C. Kato, A. Katre, J. Katzy, K. Kawade, K. Kawagoe, T. Kawamoto, G. Kawamura, V. F. Kazanin, R. Keeler, R. Kehoe, J. S. Keller, J. J. Kempster, H. Keoshkerian, O. Kepka, B. P. Kerševan, S. Kersten, R. A. Keyes, M. Khader, F. Khalil-zada, A. Khanov, A. G. Kharlamov, T. Kharlamova, T. J. Khoo, V. Khovanskiy, E. Khramov, J. Khubua, S. Kido, C. R. Kilby, H. Y. Kim, S. H. Kim, Y. K. Kim, N. Kimura, O. M. Kind, B. T. King, M. King, J. Kirk, A. E. Kiryunin, T. Kishimoto, D. Kisielewska, F. Kiss, K. Kiuchi, O. Kivernyk, E. Kladiva, M. H. Klein, M. Klein, U. Klein, K. Kleinknecht, P. Klimek, A. Klimentov, R. Klingenberg, T. Klioutchnikova, E.-E. Kluge, P. Kluit, S. Kluth, J. Knapik, E. Kneringer, E. B. F. G. Knoops, A. Knue, A. Kobayashi, D. Kobayashi, T. Kobayashi, M. Kobel, M. Kocian, P. Kodys, T. Koffas, E. Koffeman, N. M. Köhler, T. Koi, H. Kolanoski, M. Kolb, I. Koletsou, A. A. Komar, Y. Komori, T. Kondo, N. Kondrashova, K. Köneke, A. C. König, T. Kono, R. Konoplich, N. Konstantinidis, R. Kopeliansky, S. Koperny, L. Köpke, A. K. Kopp, K. Korcyl, K. Kordas, A. Korn, A. A. Korol, I. Korolkov, E. V. Korolkova, O. Kortner, S. Kortner, T. Kosek, V. V. Kostyukhin, A. Kotwal, A. Koulouris, A. Kourkoumeli-Charalampidi, C. Kourkoumelis, V. Kouskoura, A. B. Kowalewska, R. Kowalewski, T. Z. Kowalski, C. Kozakai, W. Kozanecki, A. S. Kozhin, V. A. Kramarenko, G. Kramberger, D. Krasnopevtsev, M. W. Krasny, A. Krasznahorkay, A. Kravchenko, M. Kretz, J. Kretzschmar, K. Kreutzfeldt, P. Krieger, K. Krizka, K. Kroeninger, H. Kroha, J. Kroll, J. Kroseberg, J. Krstic, U. Kruchonak, H. Krüger, N. Krumnack, M. C. Kruse, M. Kruskal, T. Kubota, H. Kucuk, S. Kuday, J. T. Kuechler, S. Kuehn, A. Kugel, F. Kuger, T. Kuhl, V. Kukhtin, R. Kukla, Y. Kulchitsky, S. Kuleshov, M. Kuna, T. Kunigo, A. Kupco, H. Kurashige, L. L. Kurchaninov, Y. A. Kurochkin, M. G. Kurth, V. Kus, E. S. Kuwertz, M. Kuze, J. Kvita, T. Kwan, D. Kyriazopoulos, A. La Rosa, J. L. La Rosa Navarro, L. La Rotonda, C. Lacasta, F. Lacava, J. Lacey, H. Lacker, D. Lacour, V. R. Lacuesta, E. Ladygin, R. Lafaye, B. Laforge, T. Lagouri, S. Lai, S. Lammers, W. Lampl, E. Lançon, U. Landgraf, M. P. J. Landon, M. C. Lanfermann, V. S. Lang, J. C. Lange, A. J. Lankford, F. Lanni, K. Lantzsch, A. Lanza, S. Laplace, C. Lapoire, J. F. Laporte, T. Lari, F. Lasagni Manghi, M. Lassnig, P. Laurelli, W. Lavrijsen, A. T. Law, P. Laycock, T. Lazovich, M. Lazzaroni, B. Le, O. Le Dortz, E. Le Guirriec, E. P. Le Quilleuc, M. LeBlanc, T. LeCompte, F. Ledroit-Guillon, C. A. Lee, S. C. Lee, L. Lee, B. Lefebvre, G. Lefebvre, M. Lefebvre, F. Legger, C. Leggett, A. Lehan, G. Lehmann Miotto, X. Lei, W. A. Leight, A. G. Leister, M. A. L. Leite, R. Leitner, D. Lellouch, B. Lemmer, K. J. C. Leney, T. Lenz, B. Lenzi, R. Leone, S. Leone, C. Leonidopoulos, S. Leontsinis, G. Lerner, C. Leroy, A. A. J. Lesage, C. G. Lester, M. Levchenko, J. Levêque, D. Levin, L. J. Levinson, M. Levy, D. Lewis, M. Leyton, B. Li, C. Li, H. Li, L. Li, L. Li, Q. Li, S. Li, X. Li, Y. Li, Z. Liang, B. Liberti, A. Liblong, P. Lichard, K. Lie, J. Liebal, W. Liebig, A. Limosani, S. C. Lin, T. H. Lin, B. E. Lindquist, A. E. Lionti, E. Lipeles, A. Lipniacka, M. Lisovyi, T. M. Liss, A. Lister, A. M. Litke, B. Liu, D. Liu, H. Liu, H. Liu, J. Liu, J. B. Liu, K. Liu, L. Liu, M. Liu, Y. L. Liu, Y. Liu, M. Livan, A. Lleres, J. Llorente Merino, S. L. Lloyd, F. Lo Sterzo, E. M. Lobodzinska, P. Loch, F. K. Loebinger, K. M. Loew, A. Loginov, T. Lohse, K. Lohwasser, M. Lokajicek, B. A. Long, J. D. Long, R. E. Long, L. Longo, K. A. Looper, J. A. Lopez Lopez, D. Lopez Mateos, B. Lopez Paredes, I. Lopez Paz, A. Lopez Solis, J. Lorenz, N. Lorenzo Martinez, M. Losada, P. J. Lösel, X. Lou, A. Lounis, J. Love, P. A. Love, H. Lu, N. Lu, H. J. Lubatti, C. Luci, A. Lucotte, C. Luedtke, F. Luehring, W. Lukas, L. Luminari, O. Lundberg, B. Lund-Jensen, P. M. Luzi, D. Lynn, R. Lysak, E. Lytken, V. Lyubushkin, H. Ma, L. L. Ma, Y. Ma, G. Maccarrone, A. Macchiolo, C. M. Macdonald, B. Maček, J. Machado Miguens, D. Madaffari, R. Madar, H. J. Maddocks, W. F. Mader, A. Madsen, J. Maeda, S. Maeland, T. Maeno, A. Maevskiy, E. Magradze, J. Mahlstedt, C. Maiani, C. Maidantchik, A. A. Maier, T. Maier, A. Maio, S. Majewski, Y. Makida, N. Makovec, B. Malaescu, Pa. Malecki, V. P. Maleev, F. Malek, U. Mallik, D. Malon, C. Malone, C. Malone, S. Maltezos, S. Malyukov, J. Mamuzic, G. Mancini, L. Mandelli, I. Mandić, J. Maneira, L. Manhaes de Andrade Filho, J. Manjarres Ramos, A. Mann, A. Manousos, B. Mansoulie, J. D. Mansour, R. Mantifel, M. Mantoani, S. Manzoni, L. Mapelli, G. Marceca, L. March, G. Marchiori, M. Marcisovsky, M. Marjanovic, D. E. Marley, F. Marroquim, S. P. Marsden, Z. Marshall, S. Marti-Garcia, B. Martin, T. A. Martin, V. J. Martin, B. Martin dit Latour, M. Martinez, V. I. Martinez Outschoorn, S. Martin-Haugh, V. S. Martoiu, A. C. Martyniuk, A. Marzin, L. Masetti, T. Mashimo, R. Mashinistov, J. Masik, A. L. Maslennikov, I. Massa, L. Massa, P. Mastrandrea, A. Mastroberardino, T. Masubuchi, P. Mättig, J. Mattmann, J. Maurer, S. J. Maxfield, D. A. Maximov, R. Mazini, I. Maznas, S. M. Mazza, N. C. Mc Fadden, G. Mc Goldrick, S. P. Mc Kee, A. McCarn, R. L. McCarthy, T. G. McCarthy, L. I. McClymont, E. F. McDonald, J. A. Mcfayden, G. Mchedlidze, S. J. McMahon, P. C. McNamara, R. A. McPherson, M. Medinnis, S. Meehan, S. Mehlhase, A. Mehta, K. Meier, C. Meineck, B. Meirose, D. Melini, B. R. Mellado Garcia, M. Melo, F. Meloni, S. B. Menary, L. Meng, X. T. Meng, A. Mengarelli, S. Menke, E. Meoni, S. Mergelmeyer, P. Mermod, L. Merola, C. Meroni, F. S. Merritt, A. Messina, J. Metcalfe, A. S. Mete, C. Meyer, C. Meyer, J.-P. Meyer, J. Meyer, H. Meyer Zu Theenhausen, F. Miano, R. P. Middleton, S. Miglioranzi, L. Mijović, G. Mikenberg, M. Mikestikova, M. Mikuž, M. Milesi, A. Milic, D. W. Miller, C. Mills, A. Milov, D. A. Milstead, A. A. Minaenko, Y. Minami, I. A. Minashvili, A. I. Mincer, B. Mindur, M. Mineev, Y. Minegishi, Y. Ming, L. M. Mir, K. P. Mistry, T. Mitani, J. Mitrevski, V. A. Mitsou, A. Miucci, P. S. Miyagawa, A. Mizukami, J. U. Mjörnmark, M. Mlynarikova, T. Moa, K. Mochizuki, P. Mogg, S. Mohapatra, S. Molander, R. Moles-Valls, R. Monden, M. C. Mondragon, K. Mönig, J. Monk, E. Monnier, A. Montalbano, J. Montejo Berlingen, F. Monticelli, S. Monzani, R. W. Moore, N. Morange, D. Moreno, M. Moreno Llácer, P. Morettini, S. Morgenstern, D. Mori, T. Mori, M. Morii, M. Morinaga, V. Morisbak, S. Moritz, A. K. Morley, G. Mornacchi, J. D. Morris, S. S. Mortensen, L. Morvaj, P. Moschovakos, M. Mosidze, H. J. Moss, J. Moss, K. Motohashi, R. Mount, E. Mountricha, E. J. W. Moyse, S. Muanza, R. D. Mudd, F. Mueller, J. Mueller, R. S. P. Mueller, T. Mueller, D. Muenstermann, P. Mullen, G. A. Mullier, F. J. Munoz Sanchez, J. A. Murillo Quijada, W. J. Murray, H. Musheghyan, M. Muškinja, A. G. Myagkov, M. Myska, B. P. Nachman, O. Nackenhorst, K. Nagai, R. Nagai, K. Nagano, Y. Nagasaka, K. Nagata, M. Nagel, E. Nagy, A. M. Nairz, Y. Nakahama, K. Nakamura, T. Nakamura, I. Nakano, R. F. Naranjo Garcia, R. Narayan, D. I. Narrias Villar, I. Naryshkin, T. Naumann, G. Navarro, R. Nayyar, H. A. Neal, P. Yu. Nechaeva, T. J. Neep, A. Negri, M. Negrini, S. Nektarijevic, C. Nellist, A. Nelson, S. Nemecek, P. Nemethy, A. A. Nepomuceno, M. Nessi, M. S. Neubauer, M. Neumann, R. M. Neves, P. Nevski, P. R. Newman, D. H. Nguyen, T. Nguyen Manh, R. B. Nickerson, R. Nicolaidou, J. Nielsen, A. Nikiforov, V. Nikolaenko, I. Nikolic-Audit, K. Nikolopoulos, J. K. Nilsen, P. Nilsson, Y. Ninomiya, A. Nisati, R. Nisius, T. Nobe, M. Nomachi, I. Nomidis, T. Nooney, S. Norberg, M. Nordberg, N. Norjoharuddeen, O. Novgorodova, S. Nowak, M. Nozaki, L. Nozka, K. Ntekas, E. Nurse, F. Nuti, F. O’grady, D. C. O’Neil, A. A. O’Rourke, V. O’Shea, F. G. Oakham, H. Oberlack, T. Obermann, J. Ocariz, A. Ochi, I. Ochoa, J. P. Ochoa-Ricoux, S. Oda, S. Odaka, H. Ogren, A. Oh, S. H. Oh, C. C. Ohm, H. Ohman, H. Oide, H. Okawa, Y. Okumura, T. Okuyama, A. Olariu, L. F. Oleiro Seabra, S. A. Olivares Pino, D. Oliveira Damazio, A. Olszewski, J. Olszowska, A. Onofre, K. Onogi, P. U. E. Onyisi, M. J. Oreglia, Y. Oren, D. Orestano, N. Orlando, R. S. Orr, B. Osculati, R. Ospanov, G. Otero y Garzon, H. Otono, M. Ouchrif, F. Ould-Saada, A. Ouraou, K. P. Oussoren, Q. Ouyang, M. Owen, R. E. Owen, V. E. Ozcan, N. Ozturk, K. Pachal, A. Pacheco Pages, L. Pacheco Rodriguez, C. Padilla Aranda, M. Pagáčová, S. Pagan Griso, M. Paganini, F. Paige, P. Pais, K. Pajchel, G. Palacino, S. Palazzo, S. Palestini, M. Palka, D. Pallin, E. St. Panagiotopoulou, I. Panagoulias, C. E. Pandini, J. G. Panduro Vazquez, P. Pani, S. Panitkin, D. Pantea, L. Paolozzi, Th. D. Papadopoulou, K. Papageorgiou, A. Paramonov, D. Paredes Hernandez, A. J. Parker, M. A. Parker, K. A. Parker, F. Parodi, J. A. Parsons, U. Parzefall, V. R. Pascuzzi, E. Pasqualucci, S. Passaggio, Fr. Pastore, G. Pásztor, S. Pataraia, J. R. Pater, T. Pauly, J. Pearce, B. Pearson, L. E. Pedersen, M. Pedersen, S. Pedraza Lopez, R. Pedro, S. V. Peleganchuk, O. Penc, C. Peng, H. Peng, J. Penwell, B. S. Peralva, M. M. Perego, D. V. Perepelitsa, E. Perez Codina, L. Perini, H. Pernegger, S. Perrella, R. Peschke, V. D. Peshekhonov, K. Peters, R. F. Y. Peters, B. A. Petersen, T. C. Petersen, E. Petit, A. Petridis, C. Petridou, P. Petroff, E. Petrolo, M. Petrov, F. Petrucci, N. E. Pettersson, A. Peyaud, R. Pezoa, P. W. Phillips, G. Piacquadio, E. Pianori, A. Picazio, E. Piccaro, M. Piccinini, M. A. Pickering, R. Piegaia, J. E. Pilcher, A. D. Pilkington, A. W. J. Pin, M. Pinamonti, J. L. Pinfold, A. Pingel, S. Pires, H. Pirumov, M. Pitt, L. Plazak, M.-A. Pleier, V. Pleskot, E. Plotnikova, D. Pluth, R. Poettgen, L. Poggioli, D. Pohl, G. Polesello, A. Poley, A. Policicchio, R. Polifka, A. Polini, C. S. Pollard, V. Polychronakos, K. Pommès, L. Pontecorvo, B. G. Pope, G. A. Popeneciu, A. Poppleton, S. Pospisil, K. Potamianos, I. N. Potrap, C. J. Potter, C. T. Potter, G. Poulard, J. Poveda, V. Pozdnyakov, M. E. Pozo Astigarraga, P. Pralavorio, A. Pranko, S. Prell, D. Price, L. E. Price, M. Primavera, S. Prince, K. Prokofiev, F. Prokoshin, S. Protopopescu, J. Proudfoot, M. Przybycien, D. Puddu, M. Purohit, P. Puzo, J. Qian, G. Qin, Y. Qin, A. Quadt, W. B. Quayle, M. Queitsch-Maitland, D. Quilty, S. Raddum, V. Radeka, V. Radescu, S. K. Radhakrishnan, P. Radloff, P. Rados, F. Ragusa, G. Rahal, J. A. Raine, S. Rajagopalan, M. Rammensee, C. Rangel-Smith, M. G. Ratti, D. M. Rauch, F. Rauscher, S. Rave, T. Ravenscroft, I. Ravinovich, M. Raymond, A. L. Read, N. P. Readioff, M. Reale, D. M. Rebuzzi, A. Redelbach, G. Redlinger, R. Reece, R. G. Reed, K. Reeves, L. Rehnisch, J. Reichert, A. Reiss, C. Rembser, H. Ren, M. Rescigno, S. Resconi, O. L. Rezanova, P. Reznicek, R. Rezvani, R. Richter, S. Richter, E. Richter-Was, O. Ricken, M. Ridel, P. Rieck, C. J. Riegel, J. Rieger, O. Rifki, M. Rijssenbeek, A. Rimoldi, M. Rimoldi, L. Rinaldi, B. Ristić, E. Ritsch, I. Riu, F. Rizatdinova, E. Rizvi, C. Rizzi, S. H. Robertson, A. Robichaud-Veronneau, D. Robinson, J. E. M. Robinson, A. Robson, C. Roda, Y. Rodina, A. Rodriguez Perez, D. Rodriguez Rodriguez, S. Roe, C. S. Rogan, O. Røhne, J. Roloff, A. Romaniouk, M. Romano, S. M. Romano Saez, E. Romero Adam, N. Rompotis, M. Ronzani, L. Roos, E. Ros, S. Rosati, K. Rosbach, P. Rose, N.-A. Rosien, V. Rossetti, E. Rossi, L. P. Rossi, J. H. N. Rosten, R. Rosten, M. Rotaru, I. Roth, J. Rothberg, D. Rousseau, A. Rozanov, Y. Rozen, X. Ruan, F. Rubbo, M. S. Rudolph, F. Rühr, A. Ruiz-Martinez, Z. Rurikova, N. A. Rusakovich, A. Ruschke, H. L. Russell, J. P. Rutherfoord, N. Ruthmann, Y. F. Ryabov, M. Rybar, G. Rybkin, S. Ryu, A. Ryzhov, G. F. Rzehorz, A. F. Saavedra, G. Sabato, S. Sacerdoti, H. F.-W. Sadrozinski, R. Sadykov, F. Safai Tehrani, P. Saha, M. Sahinsoy, M. Saimpert, T. Saito, H. Sakamoto, Y. Sakurai, G. Salamanna, A. Salamon, J. E. Salazar Loyola, D. Salek, P. H. Sales De Bruin, D. Salihagic, A. Salnikov, J. Salt, D. Salvatore, F. Salvatore, A. Salvucci, A. Salzburger, D. Sammel, D. Sampsonidis, J. Sánchez, V. Sanchez Martinez, A. Sanchez Pineda, H. Sandaker, R. L. Sandbach, M. Sandhoff, C. Sandoval, D. P. C. Sankey, M. Sannino, A. Sansoni, C. Santoni, R. Santonico, H. Santos, I. Santoyo Castillo, K. Sapp, A. Sapronov, J. G. Saraiva, B. Sarrazin, O. Sasaki, K. Sato, E. Sauvan, G. Savage, P. Savard, N. Savic, C. Sawyer, L. Sawyer, J. Saxon, C. Sbarra, A. Sbrizzi, T. Scanlon, D. A. Scannicchio, M. Scarcella, V. Scarfone, J. Schaarschmidt, P. Schacht, B. M. Schachtner, D. Schaefer, L. Schaefer, R. Schaefer, J. Schaeffer, S. Schaepe, S. Schaetzel, U. Schäfer, A. C. Schaffer, D. Schaile, R. D. Schamberger, V. Scharf, V. A. Schegelsky, D. Scheirich, M. Schernau, C. Schiavi, S. Schier, C. Schillo, M. Schioppa, S. Schlenker, K. R. Schmidt-Sommerfeld, K. Schmieden, C. Schmitt, S. Schmitt, S. Schmitz, B. Schneider, U. Schnoor, L. Schoeffel, A. Schoening, B. D. Schoenrock, E. Schopf, M. Schott, J. F. P. Schouwenberg, J. Schovancova, S. Schramm, M. Schreyer, N. Schuh, A. Schulte, M. J. Schultens, H.-C. Schultz-Coulon, H. Schulz, M. Schumacher, B. A. Schumm, Ph. Schune, A. Schwartzman, T. A. Schwarz, H. Schweiger, Ph. Schwemling, R. Schwienhorst, J. Schwindling, T. Schwindt, G. Sciolla, F. Scuri, F. Scutti, J. Searcy, P. Seema, S. C. Seidel, A. Seiden, F. Seifert, J. M. Seixas, G. Sekhniaidze, K. Sekhon, S. J. Sekula, D. M. Seliverstov, N. Semprini-Cesari, C. Serfon, L. Serin, L. Serkin, M. Sessa, R. Seuster, H. Severini, T. Sfiligoj, F. Sforza, A. Sfyrla, E. Shabalina, N. W. Shaikh, L. Y. Shan, R. Shang, J. T. Shank, M. Shapiro, P. B. Shatalov, K. Shaw, S. M. Shaw, A. Shcherbakova, C. Y. Shehu, P. Sherwood, L. Shi, S. Shimizu, C. O. Shimmin, M. Shimojima, S. Shirabe, M. Shiyakova, A. Shmeleva, D. Shoaleh Saadi, M. J. Shochet, S. Shojaii, D. R. Shope, S. Shrestha, E. Shulga, M. A. Shupe, P. Sicho, A. M. Sickles, P. E. Sidebo, E. Sideras Haddad, O. Sidiropoulou, D. Sidorov, A. Sidoti, F. Siegert, Dj. Sijacki, J. Silva, S. B. Silverstein, V. Simak, Lj. Simic, S. Simion, E. Simioni, B. Simmons, D. Simon, M. Simon, P. Sinervo, N. B. Sinev, M. Sioli, G. Siragusa, S. Yu. Sivoklokov, J. Sjölin, M. B. Skinner, H. P. Skottowe, P. Skubic, M. Slater, T. Slavicek, M. Slawinska, K. Sliwa, R. Slovak, V. Smakhtin, B. H. Smart, L. Smestad, J. Smiesko, S. Yu. Smirnov, Y. Smirnov, L. N. Smirnova, O. Smirnova, J. W. Smith, M. N. K. Smith, R. W. Smith, M. Smizanska, K. Smolek, A. A. Snesarev, I. M. Snyder, S. Snyder, R. Sobie, F. Socher, A. Soffer, D. A. Soh, G. Sokhrannyi, C. A. Solans Sanchez, M. Solar, E. Yu. Soldatov, U. Soldevila, A. A. Solodkov, A. Soloshenko, O. V. Solovyanov, V. Solovyev, P. Sommer, H. Son, H. Y. Song, A. Sood, A. Sopczak, V. Sopko, V. Sorin, D. Sosa, C. L. Sotiropoulou, R. Soualah, A. M. Soukharev, D. South, B. C. Sowden, S. Spagnolo, M. Spalla, M. Spangenberg, F. Spanò, D. Sperlich, F. Spettel, T. M. Spieker, R. Spighi, G. Spigo, L. A. Spiller, M. Spousta, R. D. St. Denis, A. Stabile, R. Stamen, S. Stamm, E. Stanecka, R. W. Stanek, C. Stanescu, M. Stanescu-Bellu, M. M. Stanitzki, S. Stapnes, E. A. Starchenko, G. H. Stark, J. Stark, P. Staroba, P. Starovoitov, S. Stärz, R. Staszewski, P. Steinberg, B. Stelzer, H. J. Stelzer, O. Stelzer-Chilton, H. Stenzel, G. A. Stewart, J. A. Stillings, M. C. Stockton, M. Stoebe, G. Stoicea, P. Stolte, S. Stonjek, A. R. Stradling, A. Straessner, M. E. Stramaglia, J. Strandberg, S. Strandberg, A. Strandlie, M. Strauss, P. Strizenec, R. Ströhmer, D. M. Strom, R. Stroynowski, A. Strubig, S. A. Stucci, B. Stugu, N. A. Styles, D. Su, J. Su, S. Suchek, Y. Sugaya, M. Suk, V. V. Sulin, S. Sultansoy, T. Sumida, S. Sun, X. Sun, J. E. Sundermann, K. Suruliz, C. J. E. Suster, M. R. Sutton, S. Suzuki, M. Svatos, M. Swiatlowski, S. P. Swift, I. Sykora, T. Sykora, D. Ta, C. Taccini, K. Tackmann, J. Taenzer, A. Taffard, R. Tafirout, N. Taiblum, H. Takai, R. Takashima, T. Takeshita, Y. Takubo, M. Talby, A. A. Talyshev, K. G. Tan, J. Tanaka, M. Tanaka, R. Tanaka, S. Tanaka, R. Tanioka, B. B. Tannenwald, S. Tapia Araya, S. Tapprogge, S. Tarem, G. F. Tartarelli, P. Tas, M. Tasevsky, T. Tashiro, E. Tassi, A. Tavares Delgado, Y. Tayalati, A. C. Taylor, G. N. Taylor, P. T. E. Taylor, W. Taylor, F. A. Teischinger, P. Teixeira-Dias, K. K. Temming, D. Temple, H. Ten Kate, P. K. Teng, J. J. Teoh, F. Tepel, S. Terada, K. Terashi, J. Terron, S. Terzo, M. Testa, R. J. Teuscher, T. Theveneaux-Pelzer, J. P. Thomas, J. Thomas-Wilsker, P. D. Thompson, A. S. Thompson, L. A. Thomsen, E. Thomson, M. J. Tibbetts, R. E. Ticse Torres, V. O. Tikhomirov, Yu. A. Tikhonov, S. Timoshenko, P. Tipton, S. Tisserant, K. Todome, T. Todorov, S. Todorova-Nova, J. Tojo, S. Tokár, K. Tokushuku, E. Tolley, L. Tomlinson, M. Tomoto, L. Tompkins, K. Toms, B. Tong, P. Tornambe, E. Torrence, H. Torres, E. Torró Pastor, J. Toth, F. Touchard, D. R. Tovey, T. Trefzger, A. Tricoli, I. M. Trigger, S. Trincaz-Duvoid, M. F. Tripiana, W. Trischuk, B. Trocmé, A. Trofymov, C. Troncon, M. Trottier-McDonald, M. Trovatelli, L. Truong, M. Trzebinski, A. Trzupek, J. C.-L. Tseng, P. V. Tsiareshka, G. Tsipolitis, N. Tsirintanis, S. Tsiskaridze, V. Tsiskaridze, E. G. Tskhadadze, K. M. Tsui, I. I. Tsukerman, V. Tsulaia, S. Tsuno, D. Tsybychev, Y. Tu, A. Tudorache, V. Tudorache, T. T. Tulbure, A. N. Tuna, S. A. Tupputi, S. Turchikhin, D. Turgeman, I. Turk Cakir, R. Turra, P. M. Tuts, G. Ucchielli, I. Ueda, M. Ughetto, F. Ukegawa, G. Unal, A. Undrus, G. Unel, F. C. Ungaro, Y. Unno, C. Unverdorben, J. Urban, P. Urquijo, P. Urrejola, G. Usai, J. Usui, L. Vacavant, V. Vacek, B. Vachon, C. Valderanis, E. Valdes Santurio, N. Valencic, S. Valentinetti, A. Valero, L. Valery, S. Valkar, J. A. Valls Ferrer, W. Van Den Wollenberg, P. C. Van Der Deijl, H. van der Graaf, N. van Eldik, P. van Gemmeren, J. Van Nieuwkoop, I. van Vulpen, M. C. van Woerden, M. Vanadia, W. Vandelli, R. Vanguri, A. Vaniachine, P. Vankov, G. Vardanyan, R. Vari, E. W. Varnes, T. Varol, D. Varouchas, A. Vartapetian, K. E. Varvell, J. G. Vasquez, G. A. Vasquez, F. Vazeille, T. Vazquez Schroeder, J. Veatch, V. Veeraraghavan, L. M. Veloce, F. Veloso, S. Veneziano, A. Ventura, M. Venturi, N. Venturi, A. Venturini, V. Vercesi, M. Verducci, W. Verkerke, J. C. Vermeulen, A. Vest, M. C. Vetterli, O. Viazlo, I. Vichou, T. Vickey, O. E. Vickey Boeriu, G. H. A. Viehhauser, S. Viel, L. Vigani, M. Villa, M. Villaplana Perez, E. Vilucchi, M. G. Vincter, V. B. Vinogradov, C. Vittori, I. Vivarelli, S. Vlachos, M. Vlasak, M. Vogel, P. Vokac, G. Volpi, M. Volpi, H. von der Schmitt, E. von Toerne, V. Vorobel, K. Vorobev, M. Vos, R. Voss, J. H. Vossebeld, N. Vranjes, M. Vranjes Milosavljevic, V. Vrba, M. Vreeswijk, R. Vuillermet, I. Vukotic, P. Wagner, W. Wagner, H. Wahlberg, S. Wahrmund, J. Wakabayashi, J. Walder, R. Walker, W. Walkowiak, V. Wallangen, C. Wang, C. Wang, F. Wang, H. Wang, H. Wang, J. Wang, J. Wang, K. Wang, R. Wang, S. M. Wang, T. Wang, W. Wang, C. Wanotayaroj, A. Warburton, C. P. Ward, D. R. Wardrope, A. Washbrook, P. M. Watkins, A. T. Watson, M. F. Watson, G. Watts, S. Watts, B. M. Waugh, S. Webb, M. S. Weber, S. W. Weber, S. A. Weber, J. S. Webster, A. R. Weidberg, B. Weinert, J. Weingarten, C. Weiser, H. Weits, P. S. Wells, T. Wenaus, T. Wengler, S. Wenig, N. Wermes, M. D. Werner, P. Werner, M. Wessels, J. Wetter, K. Whalen, N. L. Whallon, A. M. Wharton, A. White, M. J. White, R. White, D. Whiteson, F. J. Wickens, W. Wiedenmann, M. Wielers, C. Wiglesworth, L. A. M. Wiik-Fuchs, A. Wildauer, F. Wilk, H. G. Wilkens, H. H. Williams, S. Williams, C. Willis, S. Willocq, J. A. Wilson, I. Wingerter-Seez, F. Winklmeier, O. J. Winston, B. T. Winter, M. Wittgen, T. M. H. Wolf, R. Wolff, M. W. Wolter, H. Wolters, S. D. Worm, B. K. Wosiek, J. Wotschack, M. J. Woudstra, K. W. Wozniak, M. Wu, M. Wu, S. L. Wu, X. Wu, Y. Wu, T. R. Wyatt, B. M. Wynne, S. Xella, Z. Xi, D. Xu, L. Xu, B. Yabsley, S. Yacoob, D. Yamaguchi, Y. Yamaguchi, A. Yamamoto, S. Yamamoto, T. Yamanaka, K. Yamauchi, Y. Yamazaki, Z. Yan, H. Yang, H. Yang, Y. Yang, Z. Yang, W.-M. Yao, Y. C. Yap, Y. Yasu, E. Yatsenko, K. H. Yau Wong, J. Ye, S. Ye, I. Yeletskikh, E. Yildirim, K. Yorita, R. Yoshida, K. Yoshihara, C. Young, C. J. S. Young, S. Youssef, D. R. Yu, J. Yu, J. M. Yu, J. Yu, L. Yuan, S. P. Y. Yuen, I. Yusuff, B. Zabinski, G. Zacharis, R. Zaidan, A. M. Zaitsev, N. Zakharchuk, J. Zalieckas, A. Zaman, S. Zambito, L. Zanello, D. Zanzi, C. Zeitnitz, M. Zeman, A. Zemla, J. C. Zeng, Q. Zeng, O. Zenin, T. Ženiš, D. Zerwas, D. Zhang, F. Zhang, G. Zhang, H. Zhang, J. Zhang, L. Zhang, L. Zhang, M. Zhang, R. Zhang, R. Zhang, X. Zhang, Z. Zhang, X. Zhao, Y. Zhao, Z. Zhao, A. Zhemchugov, J. Zhong, B. Zhou, C. Zhou, L. Zhou, L. Zhou, M. Zhou, N. Zhou, C. G. Zhu, H. Zhu, J. Zhu, Y. Zhu, X. Zhuang, K. Zhukov, A. Zibell, D. Zieminska, N. I. Zimine, C. Zimmermann, S. Zimmermann, Z. Zinonos, M. Zinser, M. Ziolkowski, L. Živković, G. Zobernig, A. Zoccoli, M. zur Nedden, L. Zwalinski

**Affiliations:** 1 Department of Physics, University of Adelaide, Adelaide, Australia; 2 Physics Department, SUNY Albany, Albany, NY USA; 3 Department of Physics, University of Alberta, Edmonton, AB Canada; 4 Department of Physics, Ankara University, Ankara, Turkey; 5 Istanbul Aydin University, Istanbul, Turkey; 6 Division of Physics, TOBB University of Economics and Technology, Ankara, Turkey; 7 LAPP, CNRS/IN2P3 and Université Savoie Mont Blanc, Annecy-le-Vieux, France; 8 High Energy Physics Division, Argonne National Laboratory, Argonne, IL USA; 9 Department of Physics, University of Arizona, Tucson, AZ USA; 10 Department of Physics, The University of Texas at Arlington, Arlington, TX USA; 11 Physics Department, National and Kapodistrian University of Athens, Athens, Greece; 12 Physics Department, National Technical University of Athens, Zografou, Greece; 13 Department of Physics, The University of Texas at Austin, Austin, TX USA; 14 Institute of Physics, Azerbaijan Academy of Sciences, Baku, Azerbaijan; 15 Institut de Física d’Altes Energies (IFAE), The Barcelona Institute of Science and Technology, Barcelona, Spain; 16 Institute of Physics, University of Belgrade, Belgrade, Serbia; 17 Department for Physics and Technology, University of Bergen, Bergen, Norway; 18 Physics Division, Lawrence Berkeley National Laboratory and University of California, Berkeley, CA USA; 19 Department of Physics, Humboldt University, Berlin, Germany; 20 Albert Einstein Center for Fundamental Physics and Laboratory for High Energy Physics, University of Bern, Bern, Switzerland; 21 School of Physics and Astronomy, University of Birmingham, Birmingham, UK; 22 Department of Physics, Bogazici University, Istanbul, Turkey; 23 Department of Physics Engineering, Gaziantep University, Gaziantep, Turkey; 24 Faculty of Engineering and Natural Sciences, Istanbul Bilgi University, Istanbul, Turkey; 25 Faculty of Engineering and Natural Sciences, Bahcesehir University, Istanbul, Turkey; 26 Centro de Investigaciones, Universidad Antonio Narino, Bogotá, Colombia; 27 INFN Sezione di Bologna, Bologna, Italy; 28 Dipartimento di Fisica e Astronomia, Università di Bologna, Bologna, Italy; 29 Physikalisches Institut, University of Bonn, Bonn, Germany; 30 Department of Physics, Boston University, Boston, MA USA; 31 Department of Physics, Brandeis University, Waltham, MA USA; 32 Universidade Federal do Rio De Janeiro COPPE/EE/IF, Rio de Janeiro, Brazil; 33 Electrical Circuits Department, Federal University of Juiz de Fora (UFJF), Juiz de Fora, Brazil; 34 Federal University of Sao Joao del Rei (UFSJ), Sao Joao del Rei, Brazil; 35 Instituto de Fisica, Universidade de Sao Paulo, Sao Paulo, Brazil; 36 Physics Department, Brookhaven National Laboratory, Upton, NY USA; 37 Transilvania University of Brasov, Brasov, Romania; 38 Horia Hulubei National Institute of Physics and Nuclear Engineering, Bucharest, Romania; 39 Physics Department, National Institute for Research and Development of Isotopic and Molecular Technologies, Cluj-Napoca, Romania; 40 University Politehnica Bucharest, Bucharest, Romania; 41 West University in Timisoara, Timisoara, Romania; 42 Departamento de Física, Universidad de Buenos Aires, Buenos Aires, Argentina; 43 Cavendish Laboratory, University of Cambridge, Cambridge, UK; 44 Department of Physics, Carleton University, Ottawa, ON Canada; 45 CERN, Geneva, Switzerland; 46 Enrico Fermi Institute, University of Chicago, Chicago, IL USA; 47 Departamento de Física, Pontificia Universidad Católica de Chile, Santiago, Chile; 48 Departamento de Física, Universidad Técnica Federico Santa María, Valparaiso, Chile; 49 Institute of High Energy Physics, Chinese Academy of Sciences, Beijing, China; 50 Department of Physics, Nanjing University, Nanjing, Jiangsu China; 51 Physics Department, Tsinghua University, Beijing, 100084 China; 52 Department of Modern Physics, University of Science and Technology of China, Hefei, Anhui China; 53 School of Physics, Shandong University, Jinan, Shandong China; 54 Department of Physics and Astronomy, Shanghai Key Laboratory for Particle Physics and Cosmology, Shanghai Jiao Tong University (also affiliated with PKU-CHEP), Shanghai, China; 55 Laboratoire de Physique Corpusculaire, Université Clermont Auvergne, Université Blaise Pascal, CNRS/IN2P3, Clermont-Ferrand, France; 56 Nevis Laboratory, Columbia University, Irvington, NY USA; 57 Niels Bohr Institute, University of Copenhagen, Copenhagen, Denmark; 58 INFN Gruppo Collegato di Cosenza, Laboratori Nazionali di Frascati, Frascati, Italy; 59 Dipartimento di Fisica, Università della Calabria, Rende, Italy; 60 Faculty of Physics and Applied Computer Science, AGH University of Science and Technology, Kraków, Poland; 61 Marian Smoluchowski Institute of Physics, Jagiellonian University, Kraków, Poland; 62 Institute of Nuclear Physics Polish Academy of Sciences, Kraków, Poland; 63 Physics Department, Southern Methodist University, Dallas, TX USA; 64 Physics Department, University of Texas at Dallas, Richardson, TX USA; 65 DESY, Hamburg and Zeuthen, Germany; 66 Lehrstuhl für Experimentelle Physik IV, Technische Universität Dortmund, Dortmund, Germany; 67 Institut für Kern- und Teilchenphysik, Technische Universität Dresden, Dresden, Germany; 68 Department of Physics, Duke University, Durham, NC USA; 69 SUPA-School of Physics and Astronomy, University of Edinburgh, Edinburgh, UK; 70 INFN Laboratori Nazionali di Frascati, Frascati, Italy; 71 Fakultät für Mathematik und Physik, Albert-Ludwigs-Universität, Freiburg, Germany; 72 Departement de Physique Nucleaire et Corpusculaire, Université de Genève, Geneva, Switzerland; 73 INFN Sezione di Genova, Genoa, Italy; 74 Dipartimento di Fisica, Università di Genova, Genoa, Italy; 75 E. Andronikashvili Institute of Physics, Iv. Javakhishvili Tbilisi State University, Tbilisi, Georgia; 76 High Energy Physics Institute, Tbilisi State University, Tbilisi, Georgia; 77 II Physikalisches Institut, Justus-Liebig-Universität Giessen, Giessen, Germany; 78 SUPA-School of Physics and Astronomy, University of Glasgow, Glasgow, UK; 79 II Physikalisches Institut, Georg-August-Universität, Göttingen, Germany; 80 Laboratoire de Physique Subatomique et de Cosmologie, Université Grenoble-Alpes, CNRS/IN2P3, Grenoble, France; 81 Laboratory for Particle Physics and Cosmology, Harvard University, Cambridge, MA USA; 82 Kirchhoff-Institut für Physik, Ruprecht-Karls-Universität Heidelberg, Heidelberg, Germany; 83 Physikalisches Institut, Ruprecht-Karls-Universität Heidelberg, Heidelberg, Germany; 84 ZITI Institut für technische Informatik, Ruprecht-Karls-Universität Heidelberg, Mannheim, Germany; 85 Faculty of Applied Information Science, Hiroshima Institute of Technology, Hiroshima, Japan; 86 Department of Physics, The Chinese University of Hong Kong, Shatin, N.T. Hong Kong; 87 Department of Physics, The University of Hong Kong, Hong Kong, China; 88 Department of Physics and Institute for Advanced Study, The Hong Kong University of Science and Technology, Clear Water Bay, Kowloon, Hong Kong, China; 89 Department of Physics, National Tsing Hua University, Taiwan, Taiwan; 90 Department of Physics, Indiana University, Bloomington, IN USA; 91 Institut für Astro- und Teilchenphysik, Leopold-Franzens-Universität, Innsbruck, Austria; 92 University of Iowa, Iowa City, IA USA; 93 Department of Physics and Astronomy, Iowa State University, Ames, IA USA; 94 Joint Institute for Nuclear Research, JINR Dubna, Dubna, Russia; 95 KEK, High Energy Accelerator Research Organization, Tsukuba, Japan; 96 Graduate School of Science, Kobe University, Kobe, Japan; 97 Faculty of Science, Kyoto University, Kyoto, Japan; 98 Kyoto University of Education, Kyoto, Japan; 99 Department of Physics, Kyushu University, Fukuoka, Japan; 100 Instituto de Física La Plata, Universidad Nacional de La Plata and CONICET, La Plata, Argentina; 101 Physics Department, Lancaster University, Lancaster, UK; 102 INFN Sezione di Lecce, Lecce, Italy; 103 Dipartimento di Matematica e Fisica, Università del Salento, Lecce, Italy; 104 Oliver Lodge Laboratory, University of Liverpool, Liverpool, UK; 105 Department of Experimental Particle Physics, Jožef Stefan Institute and Department of Physics, University of Ljubljana, Ljubljana, Slovenia; 106 School of Physics and Astronomy, Queen Mary University of London, London, UK; 107 Department of Physics, Royal Holloway University of London, Surrey, UK; 108 Department of Physics and Astronomy, University College London, London, UK; 109 Louisiana Tech University, Ruston, LA USA; 110 Laboratoire de Physique Nucléaire et de Hautes Energies, UPMC and Université Paris-Diderot and CNRS/IN2P3, Paris, France; 111 Fysiska institutionen, Lunds universitet, Lund, Sweden; 112 Departamento de Fisica Teorica C-15, Universidad Autonoma de Madrid, Madrid, Spain; 113 Institut für Physik, Universität Mainz, Mainz, Germany; 114 School of Physics and Astronomy, University of Manchester, Manchester, UK; 115 CPPM, Aix-Marseille Université and CNRS/IN2P3, Marseille, France; 116 Department of Physics, University of Massachusetts, Amherst, MA USA; 117 Department of Physics, McGill University, Montreal, QC Canada; 118 School of Physics, University of Melbourne, Victoria, Australia; 119 Department of Physics, The University of Michigan, Ann Arbor, MI USA; 120 Department of Physics and Astronomy, Michigan State University, East Lansing, MI USA; 121 INFN Sezione di Milano, Milan, Italy; 122 Dipartimento di Fisica, Università di Milano, Milan, Italy; 123 B.I. Stepanov Institute of Physics, National Academy of Sciences of Belarus, Minsk, Republic of Belarus; 124 Research Institute for Nuclear Problems of Byelorussian State University, Minsk, Republic of Belarus; 125 Group of Particle Physics, University of Montreal, Montreal, QC Canada; 126 P.N. Lebedev Physical Institute of the Russian Academy of Sciences, Moscow, Russia; 127 Institute for Theoretical and Experimental Physics (ITEP), Moscow, Russia; 128 National Research Nuclear University MEPhI, Moscow, Russia; 129 D.V. Skobeltsyn Institute of Nuclear Physics, M.V. Lomonosov Moscow State University, Moscow, Russia; 130 Fakultät für Physik, Ludwig-Maximilians-Universität München, Munich, Germany; 131 Max-Planck-Institut für Physik (Werner-Heisenberg-Institut), Munich, Germany; 132 Nagasaki Institute of Applied Science, Nagasaki, Japan; 133 Graduate School of Science and Kobayashi-Maskawa Institute, Nagoya University, Nagoya, Japan; 134 INFN Sezione di Napoli, Naples, Italy; 135 Dipartimento di Fisica, Università di Napoli, Naples, Italy; 136 Department of Physics and Astronomy, University of New Mexico, Albuquerque, NM USA; 137 Institute for Mathematics, Astrophysics and Particle Physics, Radboud University Nijmegen/Nikhef, Nijmegen, The Netherlands; 138 Nikhef National Institute for Subatomic Physics and University of Amsterdam, Amsterdam, The Netherlands; 139 Department of Physics, Northern Illinois University, DeKalb, IL USA; 140 Budker Institute of Nuclear Physics, SB RAS, Novosibirsk, Russia; 141 Department of Physics, New York University, New York, NY USA; 142 Ohio State University, Columbus, OH USA; 143 Faculty of Science, Okayama University, Okayama, Japan; 144 Homer L. Dodge Department of Physics and Astronomy, University of Oklahoma, Norman, OK USA; 145 Department of Physics, Oklahoma State University, Stillwater, OK USA; 146 Palacký University, RCPTM, Olomouc, Czech Republic; 147 Center for High Energy Physics, University of Oregon, Eugene, OR USA; 148 LAL, Univ. Paris-Sud, CNRS/IN2P3, Université Paris-Saclay, Orsay, France; 149 Graduate School of Science, Osaka University, Osaka, Japan; 150 Department of Physics, University of Oslo, Oslo, Norway; 151 Department of Physics, Oxford University, Oxford, UK; 152 INFN Sezione di Pavia, Pavia, Italy; 153 Dipartimento di Fisica, Università di Pavia, Pavia, Italy; 154 Department of Physics, University of Pennsylvania, Philadelphia, PA USA; 155 National Research Centre “Kurchatov Institute” B.P. Konstantinov Petersburg Nuclear Physics Institute, St. Petersburg, Russia; 156 INFN Sezione di Pisa, Pisa, Italy; 157 Dipartimento di Fisica E. Fermi, Università di Pisa, Pisa, Italy; 158 Department of Physics and Astronomy, University of Pittsburgh, Pittsburgh, PA USA; 159 Laboratório de Instrumentação e Física Experimental de Partículas-LIP, Lisbon, Portugal; 160 Faculdade de Ciências, Universidade de Lisboa, Lisbon, Portugal; 161 Department of Physics, University of Coimbra, Coimbra, Portugal; 162 Centro de Física Nuclear da Universidade de Lisboa, Lisbon, Portugal; 163 Departamento de Fisica, Universidade do Minho, Braga, Portugal; 164 Departamento de Fisica Teorica y del Cosmos and CAFPE, Universidad de Granada, Granada, Spain; 165 Dep Fisica and CEFITEC of Faculdade de Ciencias e Tecnologia, Universidade Nova de Lisboa, Caparica, Portugal; 166 Institute of Physics, Academy of Sciences of the Czech Republic, Prague, Czech Republic; 167 Czech Technical University in Prague, Prague, Czech Republic; 168 Faculty of Mathematics and Physics, Charles University in Prague, Prague, Czech Republic; 169 State Research Center Institute for High Energy Physics (Protvino), NRC KI, Protvino, Russia; 170 Particle Physics Department, Rutherford Appleton Laboratory, Didcot, UK; 171 INFN Sezione di Roma, Rome, Italy; 172 Dipartimento di Fisica, Sapienza Università di Roma, Rome, Italy; 173 INFN Sezione di Roma Tor Vergata, Rome, Italy; 174 Dipartimento di Fisica, Università di Roma Tor Vergata, Rome, Italy; 175 INFN Sezione di Roma Tre, Rome, Italy; 176 Dipartimento di Matematica e Fisica, Università Roma Tre, Rome, Italy; 177 Faculté des Sciences Ain Chock, Réseau Universitaire de Physique des Hautes Energies-Université Hassan II, Casablanca, Morocco; 178 Centre National de l’Energie des Sciences Techniques Nucleaires, Rabat, Morocco; 179 Faculté des Sciences Semlalia, Université Cadi Ayyad, LPHEA-Marrakech, Marrakech, Morocco; 180 Faculté des Sciences, Université Mohamed Premier and LPTPM, Oujda, Morocco; 181 Faculté des Sciences, Université Mohammed V, Rabat, Morocco; 182 DSM/IRFU (Institut de Recherches sur les Lois Fondamentales de l’Univers), CEA Saclay (Commissariat à l’Energie Atomique et aux Energies Alternatives), Gif-sur-Yvette, France; 183 Santa Cruz Institute for Particle Physics, University of California Santa Cruz, Santa Cruz, CA USA; 184 Department of Physics, University of Washington, Seattle, WA USA; 185 Department of Physics and Astronomy, University of Sheffield, Sheffield, UK; 186 Department of Physics, Shinshu University, Nagano, Japan; 187 Fachbereich Physik, Universität Siegen, Siegen, Germany; 188 Department of Physics, Simon Fraser University, Burnaby, BC Canada; 189 SLAC National Accelerator Laboratory, Stanford, CA USA; 190 Faculty of Mathematics, Physics and Informatics, Comenius University, Bratislava, Slovak Republic; 191 Department of Subnuclear Physics, Institute of Experimental Physics of the Slovak Academy of Sciences, Kosice, Slovak Republic; 192 Department of Physics, University of Cape Town, Cape Town, South Africa; 193 Department of Physics, University of Johannesburg, Johannesburg, South Africa; 194 School of Physics, University of the Witwatersrand, Johannesburg, South Africa; 195 Department of Physics, Stockholm University, Stockholm, Sweden; 196 The Oskar Klein Centre, Stockholm, Sweden; 197 Physics Department, Royal Institute of Technology, Stockholm, Sweden; 198 Departments of Physics and Astronomy and Chemistry, Stony Brook University, Stony Brook, NY USA; 199 Department of Physics and Astronomy, University of Sussex, Brighton, UK; 200 School of Physics, University of Sydney, Sydney, Australia; 201 Institute of Physics, Academia Sinica, Taipei, Taiwan; 202 Department of Physics, Technion: Israel Institute of Technology, Haifa, Israel; 203 Raymond and Beverly Sackler School of Physics and Astronomy, Tel Aviv University, Tel Aviv, Israel; 204 Department of Physics, Aristotle University of Thessaloniki, Thessaloníki, Greece; 205 International Center for Elementary Particle Physics and Department of Physics, The University of Tokyo, Tokyo, Japan; 206 Graduate School of Science and Technology, Tokyo Metropolitan University, Tokyo, Japan; 207 Department of Physics, Tokyo Institute of Technology, Tokyo, Japan; 208 Tomsk State University, Tomsk, Russia; 209 Department of Physics, University of Toronto, Toronto, ON Canada; 210 INFN-TIFPA, Trento, Italy; 211 University of Trento, Trento, Italy; 212 TRIUMF, Vancouver, BC Canada; 213 Department of Physics and Astronomy, York University, Toronto, ON Canada; 214 Faculty of Pure and Applied Sciences, and Center for Integrated Research in Fundamental Science and Engineering, University of Tsukuba, Tsukuba, Japan; 215 Department of Physics and Astronomy, Tufts University, Medford, MA USA; 216 Department of Physics and Astronomy, University of California Irvine, Irvine, CA USA; 217 INFN Gruppo Collegato di Udine, Sezione di Trieste, Udine, Italy; 218 ICTP, Trieste, Italy; 219 Dipartimento di Chimica, Fisica e Ambiente, Università di Udine, Udine, Italy; 220 Department of Physics and Astronomy, University of Uppsala, Uppsala, Sweden; 221 Department of Physics, University of Illinois, Urbana, IL USA; 222 Instituto de Fisica Corpuscular (IFIC) and Departamento de Fisica Atomica, Molecular y Nuclear and Departamento de Ingeniería Electrónica and Instituto de Microelectrónica de Barcelona (IMB-CNM), University of Valencia and CSIC, Valencia, Spain; 223 Department of Physics, University of British Columbia, Vancouver, BC Canada; 224 Department of Physics and Astronomy, University of Victoria, Victoria, BC Canada; 225 Department of Physics, University of Warwick, Coventry, UK; 226 Waseda University, Tokyo, Japan; 227 Department of Particle Physics, The Weizmann Institute of Science, Rehovot, Israel; 228 Department of Physics, University of Wisconsin, Madison, WI USA; 229 Fakultät für Physik und Astronomie, Julius-Maximilians-Universität, Würzburg, Germany; 230 Fakultät für Mathematik und Naturwissenschaften, Fachgruppe Physik, Bergische Universität Wuppertal, Wuppertal, Germany; 231 Department of Physics, Yale University, New Haven, CT USA; 232 Yerevan Physics Institute, Yerevan, Armenia; 233 Centre de Calcul de l’Institut National de Physique Nucléaire et de Physique des Particules (IN2P3), Villeurbanne, France; 234 CERN, Geneva, Switzerland

## Abstract

During 2015 the ATLAS experiment recorded $3.8\,{\mathrm{fb}}^{-1}$ of proton–proton collision data at a centre-of-mass energy of $13\,{\mathrm{TeV}}$. The ATLAS trigger system is a crucial component of the experiment, responsible for selecting events of interest at a recording rate of approximately 1 kHz from up to 40 MHz of collisions. This paper presents a short overview of the changes to the trigger and data acquisition systems during the first long shutdown of the LHC and shows the performance of the trigger system and its components based on the 2015 proton–proton collision data.

## Introduction

The trigger system is an essential component of any collider experiment as it is responsible for deciding whether or not to keep an event from a given bunch-crossing interaction for later study. During Run 1 (2009 to early 2013) of the Large Hadron Collider (LHC), the trigger system [1–5] of the ATLAS experiment [6] operated efficiently at instantaneous luminosities of up to $8\times 10^{33}$ cm$^{-2}$ s$^{-1}$ and primarily at centre-of-mass energies, $\sqrt{s}$, of 7 $\text{TeV}$ and 8 $\text{TeV}$. In Run 2 (since 2015) the increased centre-of-mass energy of 13 $\text{TeV}$, higher luminosity and increased number of proton–proton interactions per bunch-crossing (pile-up) meant that, without upgrades of the trigger system, the trigger rates would have exceeded the maximum allowed rates when running with the trigger thresholds needed to satisfy the physics programme of the experiment. For this reason, the first long shutdown (LS1) between LHC Run 1 and Run 2 operations was used to improve the trigger system with almost no component left untouched.

After a brief introduction of the ATLAS detector in Sect. 2, Sect. 3 summarises the changes to the trigger and data acquisition during LS1. Section 4 gives an overview of the trigger menu used during 2015 followed by an introduction to the reconstruction algorithms used at the high-level trigger in Sect. 5. The performance of the different trigger signatures is shown in Sect. 6 for the data taken with 25 ns bunch-spacing in 2015 at a peak luminosity of $5\times 10^{33}$ cm$^{-2}$ s$^{-1}$ with comparison to Monte Carlo (MC) simulation.

## ATLAS detector

ATLAS is a general-purpose detector with a forward-backward symmetry, which provides almost full solid angle coverage around the interaction point.^1^ The main components of ATLAS are an inner detector (ID), which is surrounded by a superconducting solenoid providing a 2T axial magnetic field, a calorimeter system, and a muon spectrometer (MS) in a magnetic field generated by three large superconducting toroids with eight coils each. The ID provides track reconstruction within $|\eta | < 2.5$, employing a pixel detector (Pixel) close to the beam pipe, a silicon microstrip detector (SCT) at intermediate radii, and a transition radiation tracker (TRT) at outer radii. A new innermost pixel-detector layer, the insertable B-layer (IBL), was added during LS1 at a radius of 33 mm around a new and thinner beam pipe [7]. The calorimeter system covers the region $|\eta | < 4.9$, the forward region ($3.2< |\eta | < 4.9$) being instrumented with a liquid-argon (LAr) calorimeter for electromagnetic and hadronic measurements. In the central region, a lead/LAr electromagnetic calorimeter covers $|\eta | < 3.2$, while the hadronic calorimeter uses two different detector technologies, with steel/scintillator tiles ($|\eta | < 1.7$) or lead/LAr ($1.5< |\eta | < 3.2$) as absorber/active material. The MS consists of one barrel ($|\eta |<1.05$) and two end-cap sections ($1.05<|\eta |<2.7$). Resistive plate chambers (RPC, three doublet layers for $|\eta |<1.05$) and thin gap chambers (TGC, one triplet layer followed by two doublets for $1.0<|\eta |<2.4$) provide triggering capability as well as $(\eta ,\phi )$ position measurements. A precise momentum measurement for muons with $|\eta |$ up to 2.7 is provided by three layers of monitored drift tubes (MDT), with each chamber providing six to eight $\eta$ measurements along the muon trajectory. For $|\eta |>2$, the inner layer is instrumented with cathode strip chambers (CSC), consisting of four sensitive layers each, instead of MDTs.

**Fig. 1:**
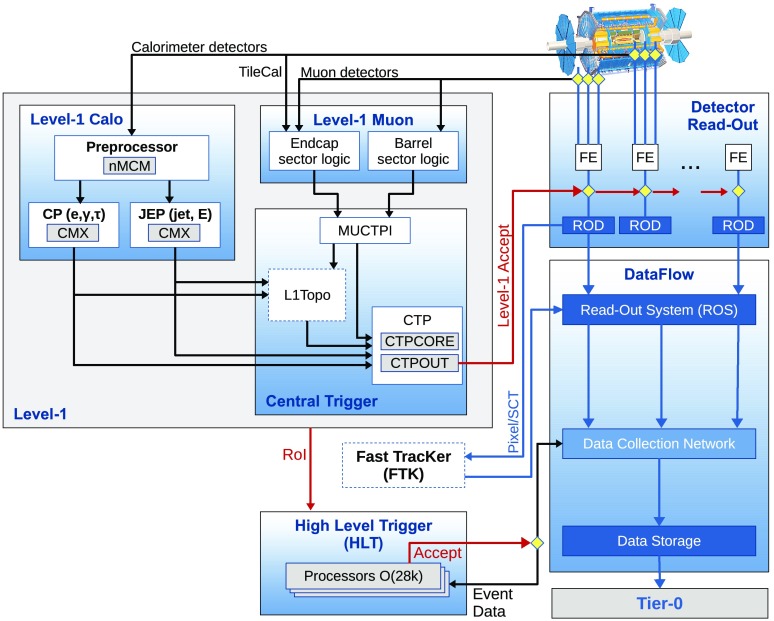
The ATLAS TDAQ system in Run 2 with emphasis on the components relevant for triggering. L1Topo and FTK were being commissioned during 2015 and not used for the results shown here

The Trigger and Data Acquisition (TDAQ) system shown in Fig. 1 consists of a hardware-based first-level trigger (L1) and a software-based high-level trigger (HLT). The L1 trigger decision is formed by the Central Trigger Processor (CTP), which receives inputs from the L1 calorimeter (L1Calo) and L1 muon (L1Muon) triggers as well as several other subsystems such as the Minimum Bias Trigger Scintillators (MBTS), the LUCID Cherenkov counter and the Zero-Degree Calorimeter (ZDC). The CTP is also responsible for applying preventive dead-time. It limits the minimum time between two consecutive L1 accepts (*simple dead-time*) to avoid overlapping readout windows, and restricts the number of L1 accepts allowed in a given number of bunch-crossings (*complex dead-time*) to avoid front-end buffers from overflowing. In 2015 running, the simple dead-time was set to 4 bunch-crossings (100 ns). A more detailed description of the L1 trigger system can be found in Ref. [1]. After the L1 trigger acceptance, the events are buffered in the Read-Out System (ROS) and processed by the HLT. The HLT receives Region-of-Interest (RoI) information from L1, which can be used for regional reconstruction in the trigger algorithms. After the events are accepted by the HLT, they are transferred to local storage at the experimental site and exported to the Tier-0 facility at CERN’s computing centre for offline reconstruction.

Several Monte Carlo simulated datasets were used to assess the performance of the trigger. Fully simulated photon+jet and dijet events generated with Pythia8 [8] using the NNPDF2.3LO [9] parton distribution function (PDF) set were used to study the photon and jet triggers. To study tau and *b*-jet triggers, $Z\rightarrow \tau \tau$ and $t\bar{t}$ samples generated with Powheg-Box 2.0 [10–12] with the CT10 [13] PDF set and interfaced to Pythia8 or Pythia6 [14] with the CTEQ6L1 [15] PDF set were used.

## Changes to the Trigger/DAQ system for Run-2

The TDAQ system used during Run 1 is described in detail in Refs. [1, 16]. Compared to Run 1, the LHC has increased its centre-of-mass energy from 8 to 13 $\text{TeV}$, and the nominal bunch-spacing has decreased from 50 to 25 ns. Due to the larger transverse beam size at the interaction point ($\beta^{*}={80}$ cm compared to 60 cm in 2012) and a lower bunch population ($1.15\times 10^{11}$ instead of $1.6\times 10^{11}$ protons per bunch) the peak luminosity reached in 2015 ($5.0\times 10^{33}$ cm$^{-2}$ s$^{-1}$) was lower than in Run 1 ($7.7\times 10^{33}$ cm$^{-2}$ s$^{-1}$). However, due to the increase in energy, trigger rates are on average 2.0 to 2.5 times larger for the same luminosity and with the same trigger criteria (individual trigger rates, e.g. jets, can have even larger increases). The decrease in bunch-spacing also increases certain trigger rates (e.g. muons) due to additional interactions from neighbouring bunch-crossings (out-of-time pile-up). In order to prepare for the expected higher rates in Run 2, several upgrades and additions were implemented during LS1. The main changes relevant to the trigger system are briefly described below.

In the L1 Central Trigger, a new topological trigger (L1Topo) consisting of two FPGA-based (Field-Programmable Gate Arrays) processor modules was added. The modules are identical hardware-wise and each is programmed to perform selections based on geometric or kinematic association between trigger objects received from the L1Calo or L1Muon systems. This includes the refined calculation of global event quantities such as missing transverse momentum (with magnitude $E_{\text{T}}^{\text{miss}}$). The system was fully installed and commissioned during 2016, i.e. it was not used for the data described in this paper. Details of the hardware implementation can be found in Ref. [17]. The Muon-to-CTP interface (MUCPTI) and the CTP were upgraded to provide inputs to and receive inputs from L1Topo, respectively. In order to better address sub-detector specific requirements, the CTP now supports up to four independent complex dead-time settings operating simultaneously. In addition, the number of L1 trigger selections (512) and bunch-group selections (16), defined later, were doubled compared to Run 1. The changes to the L1Calo and L1Muon trigger systems are described in separate sections below.

In Run 1 the HLT consisted of separate Level-2 (L2) and Event Filter (EF) farms. While L2 requested partial event data over the network, the EF operated on full event information assembled by separate farm nodes dedicated to Event Building (EB). For Run 2, the L2 and EF farms were merged into a single homogeneous farm allowing better resource sharing and an overall simplification of both the hardware and software. RoI-based reconstruction continues to be employed by time-critical algorithms. The functionality of the EB nodes was also integrated into the HLT farm. To achieve higher readout and output rates, the ROS, the data collection network and data storage system were upgraded. The on-detector front-end (FE) electronics and detector-specific readout drivers (ROD) were not changed in any significant way.

A new Fast TracKer (FTK) system [18] will provide global ID track reconstruction at the L1 trigger rate using lookup tables stored in custom associative memory chips for the pattern recognition. Instead of a computationally intensive helix fit, the FPGA-based track fitter performs a fast linear fit and the tracks are made available to the HLT. This system will allow the use of tracks at much higher event rates in the HLT than is currently affordable using CPU systems. This system is currently being installed and expected to be fully commissioned during 2017.

### Level-1 calorimeter trigger

**Fig. 2:**
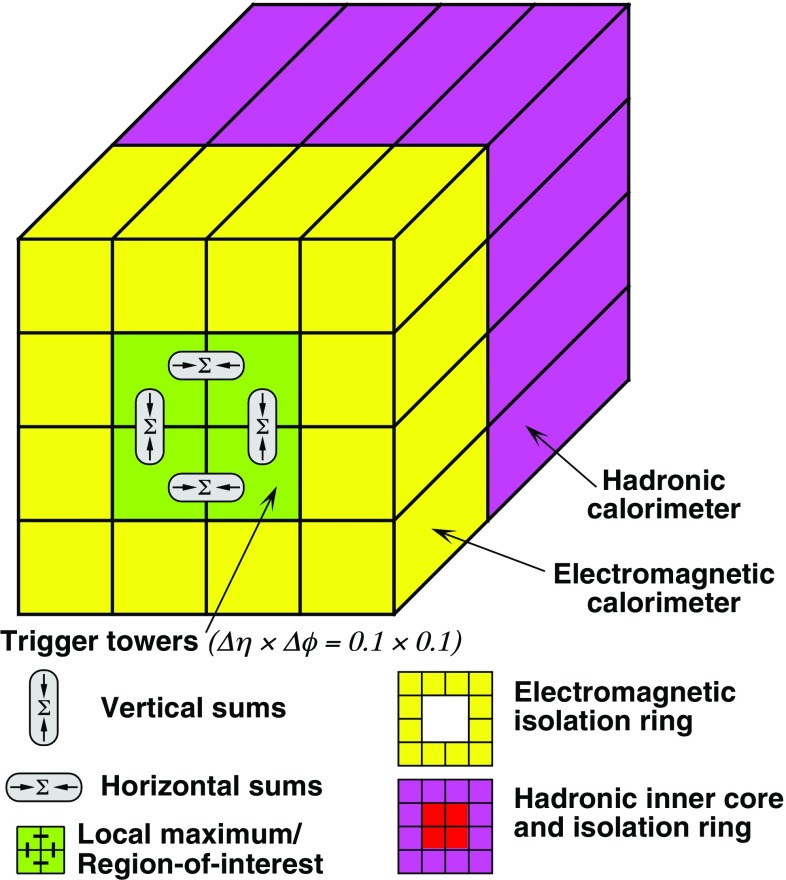
Schematic view of the trigger towers used as input to the L1Calo trigger algorithms

The details of the L1Calo trigger algorithms can be found in Ref. [19], and only the basic elements are described here. The electron/photon and tau trigger algorithm (Fig. 2) identifies an RoI as a $2\times 2$ trigger tower cluster in the electromagnetic calorimeter for which the sum of the transverse energy from at least one of the four possible pairs of nearest neighbour towers ($1\times 2$ or $2\times 1$) exceeds a predefined threshold. Isolation-veto thresholds can be set for the electromagnetic (EM) isolation ring in the electromagnetic calorimeter, as well as for hadronic tower sums in a central $2\times 2$ core behind the EM cluster and in the 12-tower hadronic ring around it. The $E_{\text{T}}$ threshold can be set differently for different $\eta$ regions at a granularity of 0.1 in $\eta$ in order to correct for varying detector energy responses. The energy of the trigger towers is calibrated at the electromagnetic energy scale (EM scale). The EM scale correctly reconstructs the energy deposited by particles in an electromagnetic shower in the calorimeter but underestimates the energy deposited by hadrons. Jet RoIs are defined as $4\times 4$ or $8\times 8$ trigger tower windows for which the summed electromagnetic and hadronic transverse energy exceeds predefined thresholds and which surround a $2\times 2$ trigger tower core that is a local maximum. The location of this local maximum also defines the coordinates of the jet RoI.

In preparation for Run 2, due to the expected increase in luminosity and consequent increase in the number of pile-up events, a major upgrade of several central components of the L1Calo electronics was undertaken to reduce the trigger rates.

**Fig. 3:**
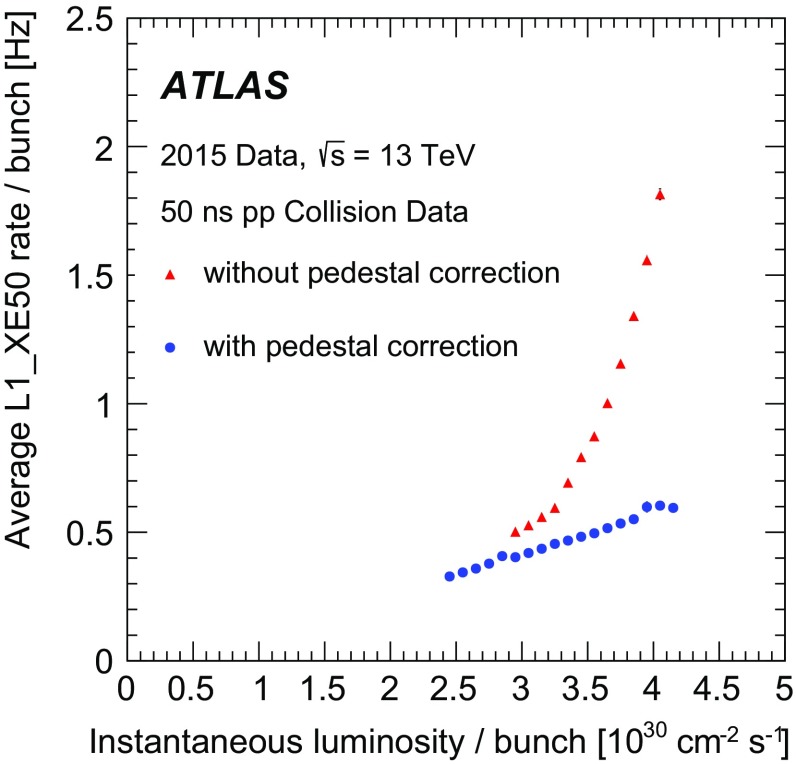
The per-bunch trigger rate for the L1 missing transverse momentum trigger with a threshold of 50 $\text{GeV}$ (L1_XE50) as a function of the instantaneous luminosity per bunch. The rates are shown with and without pedestal correction applied

For the preprocessor system [20], which digitises and calibrates the analogue signals (consisting of $\sim$7000 trigger towers at a granularity of $0.1\times 0.1$ in $\eta \times \phi$) from the calorimeter detectors, a new FPGA-based multi-chip module (nMCM) was developed [21] and about 3000 chips (including spares) were produced. They replace the old ASIC-based MCMs used during Run 1. The new modules provide additional flexibility and new functionality with respect to the old system. In particular, the nMCMs support the use of digital autocorrelation Finite Impulse Response (FIR) filters and the implementation of a dynamic, bunch-by-bunch pedestal correction, both introduced for Run 2. These improvements lead to a significant rate reduction of the L1 jet and L1 $E_{\text{T}}^{\text{miss}}$ triggers. The bunch-by-bunch pedestal subtraction compensates for the increased trigger rates at the beginning of a bunch train caused by the interplay of in-time and out-of-time pile-up coupled with the LAr pulse shape [22], and linearises the L1 trigger rate as a function of the instantaneous luminosity, as shown in Fig. 3 for the L1 $E_{\text{T}}^{\text{miss}}$ trigger. The autocorrelation FIR filters substantially improve the bunch-crossing identification (BCID) efficiencies, in particular for low energy deposits. However, the use of this new filtering scheme initially led to an early trigger signal (and incomplete events) for a small fraction of very high energy events. These events were saved into a stream dedicated to mistimed events and treated separately in the relevant physics analyses. The source of the problem was fixed in firmware by adapting the BCID decision logic for saturated pulses and was deployed at the start of the 2016 data-taking period.

The preprocessor outputs are then transmitted to both the Cluster Processor (CP) and Jet/Energy-sum Processor (JEP) subsystems in parallel. The CP subsystem identifies electron/photon and tau lepton candidates with $E_{\text{T}}$ above a programmable threshold and satisfying, if required, certain isolation criteria. The JEP receives jet trigger elements, which are $0.2\times 0.2$ sums in $\eta \times \phi$, and uses these to identify jets and to produce global sums of scalar and missing transverse momentum. Both the CP and JEP firmware were upgraded to allow an increase of the data transmission rate over the custom-made backplanes from 40 to 160 Mbps, allowing the transmission of up to four jet or five EM/tau trigger objects per module. A trigger object contains the $E_{\text{T}}$ sum, $\eta -\phi$ coordinates, and isolation thresholds where relevant. While the JEP firmware changes were only minor, substantial extra selectivity was added to the CP by implementing energy-dependent L1 electromagnetic isolation criteria instead of fixed threshold cuts. This feature was added to the trigger menu (defined in Sect. 4) at the beginning of Run 2. In 2015 it was used to effectively select events with specific signatures, e.g. EM isolation was required for taus but not for electrons.

Finally, new extended cluster merger modules (CMX) were developed to replace the L1Calo merger modules (CMMs) used during Run 1. The new CMX modules transmit the location and the energy of identified trigger objects to the new L1Topo modules instead of only the threshold multiplicities as done by the CMMs. This transmission happens with a bandwidth of 6.4 Gbps per channel, while the total output bandwidth amounts to above 2 Tbps. Moreover, for most L1 triggers, twice as many trigger selections and isolation thresholds can be processed with the new CMX modules compared to Run 1, considerably increasing the selectivity of the L1Calo system.

### Level-1 muon trigger

The muon barrel trigger was not significantly changed with respect to Run 1, apart from the regions close to the feet that support the ATLAS detector, where the presence of support structures reduces trigger coverage. To recover trigger acceptance, a fourth layer of RPC trigger chambers was installed before Run 1 in the projective region of the acceptance holes. These chambers were not operational during Run 1. During LS1, these RPC layers were equipped with trigger electronics. Commissioning started during 2015 and they are fully operational in 2016. Additional chambers were installed during LS1 to cover the acceptance holes corresponding to two elevator shafts at the bottom of the muon spectrometer but are not yet operational. At the end of the commissioning phase, the new feet and elevator chambers are expected to increase the overall barrel trigger acceptance by 2.8 and 0.8% points, respectively.

**Fig. 4:**
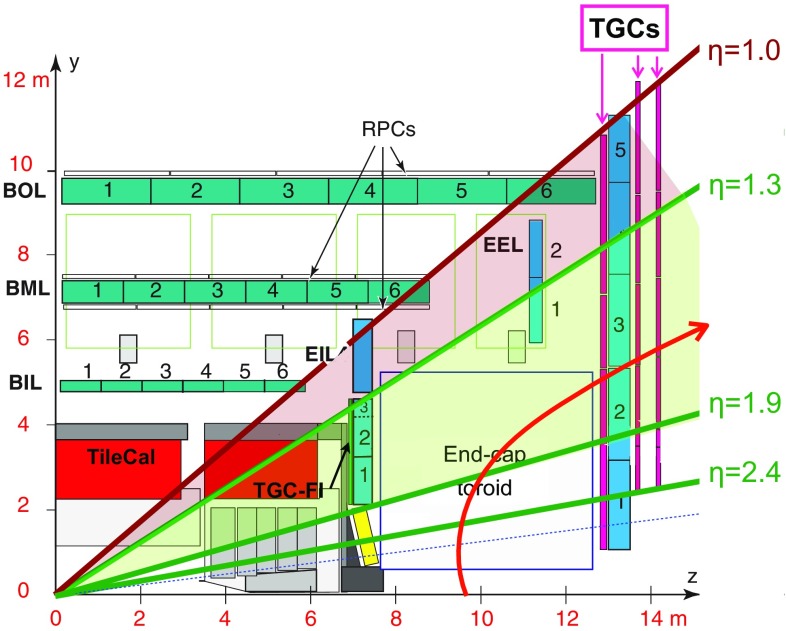
A schematic view of the muon spectrometer with lines indicating various pseudorapidity regions. The *curved arrow* shows an example of a trajectory from slow particles generated at the beam pipe around $z\sim 10\,\text{m}.$ Triggers due to events of this type are mitigated by requiring an additional coincidence with the TGC-FI chambers in the region $1.3< |\eta | < 1.9$

**Fig. 5:**
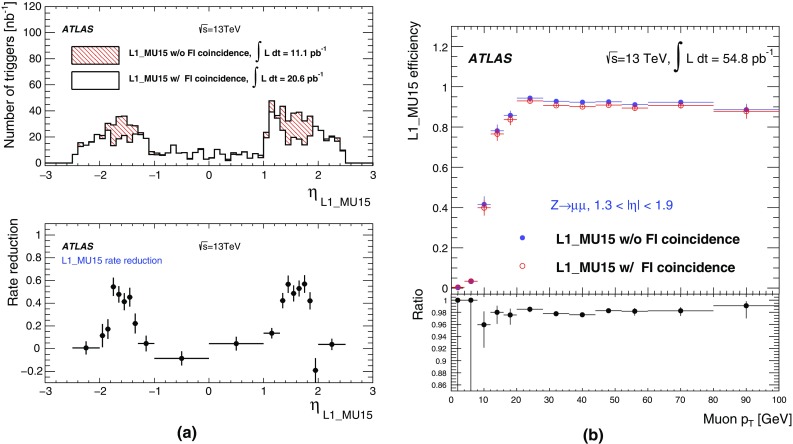
**a** Number of events with an L1 muon trigger with transverse momentum ($p_{\text{T}}$) above 15 $\text{GeV}$ (L1_MU15) as a function of the muon trigger $\eta$ coordinate, requiring a coincidence with the TGC-FI chambers (*open histogram*) and not requiring it (*cross-hatched histogram*), together with the fractional event rate reduction in the *bottom plot*. The event rate reduction in the regions with no TGC-FI chambers is consistent with zero within the uncertainty. **b** Efficiency of L1_MU15 in the end-cap region, as a function of the $p_{\text{T}}$ of the offline muon measured via a tag-and-probe method (see Sect. 6) using $Z\rightarrow \mu \mu$ events with (*open dots*) and without (*filled dots*) the TGC-FI coincidence, together with the ratio in the *bottom panel*

During Run 1, a significant fraction of the trigger rate from the end-cap region was found to be due to particles not originating from the interaction point, as illustrated in Fig. 4. To reject these interactions, new trigger logic was introduced in Run 2. An additional TGC coincidence requirement was deployed in 2015 covering the region $1.3< |\eta | < 1.9$ (TGC-FI). Further coincidence logic in the region $1.0< |\eta | < 1.3$ is being commissioned by requiring coincidence with the inner TGC chambers (EIL4) or the Tile hadronic calorimeter. Figure 5a shows the muon trigger rate as a function of the muon trigger pseudorapidity with and without the TGC-FI coincidence in separate data-taking runs. The asymmetry as a function of $\eta$ is a result of the magnetic field direction and the background particles being mostly positively charged. In the region where this additional coincidence is applied, the trigger rate is reduced by up to 60% while only about 2% of offline reconstructed muons are lost in this region, as seen in Fig. 5b.

## Trigger menu

The trigger menu defines the list of L1 and HLT triggers and consists of:

- *primary* triggers, which are used for physics analyses and are typically unprescaled;
- *support* triggers, which are used for efficiency and performance measurements or for monitoring, and are typically operated at a small rate (of the order of 0.5 Hz each) using prescale factors;
- *alternative* triggers, using alternative (sometimes experimental or new) reconstruction algorithms compared to the primary or support selections, and often heavily overlapping with the primary triggers;
- *backup* triggers, with tighter selections and lower expected rate;
- *calibration* triggers, which are used for detector calibration and are often operated at high rate but storing very small events with only the relevant information needed for calibration.

The primary triggers cover all signatures relevant to the ATLAS physics programme including electrons, photons, muons, tau leptons, (*b*-)jets and $E_{\text{T}}^{\text{miss}}$   which are used for Standard Model (SM) precision measurements including decays of the Higgs, *W* and *Z* bosons, and searches for physics beyond the SM such as heavy particles, supersymmetry or exotic particles. A set of low transverse momentum ($p_{\text{T}}$) dimuon triggers is used to collect *B*-meson decays, which are essential for the *B*-physics programme of ATLAS.

The trigger menu composition and trigger thresholds are optimised for several luminosity ranges in order to maximise the physics output of the experiment and to fit within the rate and bandwidth constraints of the ATLAS detector, TDAQ system and offline computing. For Run 2 the most relevant constraints are the maximum L1 rate of 100 kHz (75 kHz in Run 1) defined by the ATLAS detector readout capability and an average HLT physics output rate of 1000 Hz (400 Hz in Run 1) defined by the offline computing model. To ensure an optimal trigger menu within the rate constraints for a given LHC luminosity, prescale factors can be applied to L1 and HLT triggers and changed during data-taking in such a way that triggers may be disabled or only a certain fraction of events may be accepted by them. Supporting triggers may be running at a constant rate or certain triggers enabled later in the LHC fill when the luminosity and pile-up has reduced and the required resources are available. Further flexibility is provided by *bunch groups*, which allow triggers to include specific requirements on the LHC proton bunches colliding in ATLAS. These requirements include paired (colliding) bunch-crossings for physics triggers, empty or unpaired crossings for background studies or search for long-lived particle decays, and dedicated bunch groups for detector calibration.

Trigger names used throughout this paper consist of the trigger level (L1 or HLT, the latter often omitted for brevity), multiplicity, particle type (e.g. g for photon, j for jet, xe for $E_{\text{T}}^{\text{miss}}$, te for $\sum$ $E_{\text{T}}$  triggers) and $p_{\text{T}}$ threshold value in $\text{GeV}$ (e.g. L1_2MU4 requires at least two muons with $p_{\text{T}} >{4}\,{\text{GeV}}$ at L1, HLT_mu40 requires at least one muon with $p_{\text{T}} >{40}\,{\text{GeV}}$ at the HLT). L1 and HLT trigger items are written in upper case and lower case letters, respectively. Each HLT trigger is configured with an L1 trigger as its seed. The L1 seed is not explicitly part of the trigger name except when an HLT trigger is seeded by more than one L1 trigger, in which case the L1 seed is denoted in the suffix of the alternative trigger (e.g. HLT_mu20 and HLT_mu20_L1MU15 with the first one using L1_MU20 as its seed). Further selection criteria (type of identification, isolation, reconstruction algorithm, geometrical region) are suffixed to the trigger name (e.g. HLT_g120_loose).

### Physics trigger menu for 2015 data-taking

The main goal of the trigger menu design was to maintain the unprescaled single-electron and single-muon trigger $p_{\text{T}}$ thresholds around 25 $\text{GeV}$ despite the expected higher trigger rates in Run 2 (see Sect. 3). This strategy ensures the collection of the majority of the events with leptonic *W* and *Z* boson decays, which are the main source of events for the study of electroweak processes. In addition, compared to using a large number of analysis-specific triggers, this trigger strategy is simpler and more robust at the cost of slightly higher trigger output rates. Dedicated (multi-object) triggers were added for specific analyses not covered by the above. Table 1 shows a comparison of selected primary trigger thresholds for L1 and the HLT used during Run 1 and 2015 together with the typical thresholds for offline reconstructed objects used in analyses (the latter are usually defined as the $p_{\text{T}}$ value at which the trigger efficiency reached the plateau). Trigger thresholds at L1 were either kept the same as during Run 1 or slightly increased to fit within the allowed maximum L1 rate of 100 kHz. At the HLT, several selections were loosened compared to Run 1 or thresholds lowered thanks to the use of more sophisticated HLT algorithms (e.g. multivariate analysis techniques for electrons and taus).

**Table 1:** Comparison of selected primary trigger thresholds (in $\text{GeV}$) at the end of Run 1 and during 2015 together with typical offline requirements applied in analyses (the 2012 offline thresholds are not listed but have a similar relationship to the 2012 HLT thresholds). Electron and tau identification are assumed to fulfil the ‘medium’ criteria unless otherwise stated. Photon and *b*-jet identification (‘b’) are assumed to fulfil the ‘loose’ criteria. Trigger isolation is denoted by ‘i’. The details of these selections are described in Sect. 6

Year	2012		2015		
$\sqrt{s}\,$	8 $\text{TeV}$		13 $\text{TeV}$		
Peak luminosity	$7.7\times 10^{33}$ cm$^{-2}$ s$^{-1}$		$5.0\times 10^{33}$ cm$^{-2}$ s$^{-1}$		
	$p_{\text{T}}$ threshold [$\text{GeV}$], criteria				
Category	L1	HLT	L1	HLT	Offline
Single electron	18	24i	20	24	25
Single muon	15	24i	15	20i	21
Single photon	20	120	22i	120	125
Single tau	40	115	60	80	90
Single jet	75	360	100	360	400
Single *b*-jet	n/a	n/a	100	225	235
$E_{\text{T}}^{\text{miss}}$	40	80	50	70	180
Dielectron	2$\times$10	2$\times$12, loose	2$\times$10	2$\times$12, loose	15
Dimuon	2$\times$10	2$\times$13	2$\times$10	2$\times$10	11
Electron, muon	10, 6	12, 8	15, 10	17, 14	19, 15
Diphoton	16, 12	35, 25	2$\times$15	35, 25	40, 30
Ditau	15i, 11i	27, 18	20i, 12i	35, 25	40, 30
Tau, electron	11i, 14	28i, 18	12i(+jets), 15	25, 17i	30, 19
Tau, muon	8, 10	20, 15	12i(+jets), 10	25, 14	30, 15
Tau, $E_{\text{T}}^{\text{miss}}$	20, 35	38, 40	20, 45(+jets)	35, 70	40, 180
Four jets	4$\times$15	4$\times$80	3$\times$40	4$\times$85	95
Six jets	4$\times$15	6$\times$45	4$\times$15	6$\times$45	55
Two *b*-jets	75	35b, 145b	100	50b, 150b	60
Four(Two) (*b*-)jets	4$\times$15	2$\times$35b, 2$\times$35	3$\times$25	2$\times$35b, 2$\times$35	45
*B*-physics (Dimuon)	6, 4	6, 4	6, 4	6, 4	6, 4

**Fig. 6:**
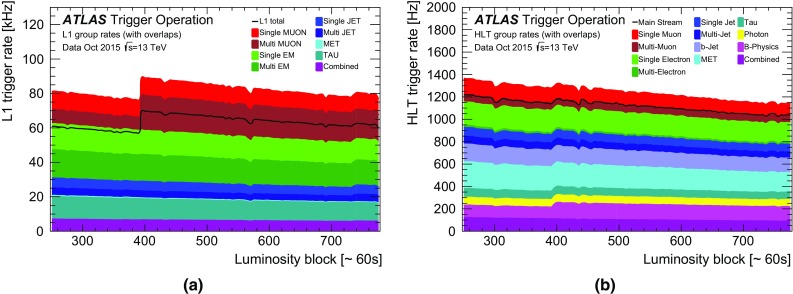
**a** L1 and **b** HLT trigger rates grouped by trigger signature during an LHC fill in October 2015 with a peak luminosity of $4.5\times 10^{33}$ cm$^{-2}$ s$^{-1}$. Due to overlaps the sum of the individual groups is higher than the **a** L1 total rate and **b** *Main* physics stream rate, which are shown as *black lines*. Multi-object triggers are included in the *b*-jets and tau groups. The rate increase around luminosity block 400 is due to the removal of prescaling of the *B*-physics triggers. The combined group includes multiple triggers combining different trigger signatures such as electrons with muons, taus, jets or $E_{\text{T}}^{\text{miss}}$

Figure 6a, b show the L1 and HLT trigger rates grouped by signatures during an LHC fill with a peak luminosity of $4.5\times 10^{33}$ cm$^{-2}$ s$^{-1}$. The preventive dead-time^2^ The single-electron and single-muon triggers contribute a large fraction to the total rate. While running at these relatively low luminosities it was possible to dedicate a large fraction of the bandwidth to the *B*-physics triggers. Support triggers contribute about 20$\%$ of the total rate. Since the time for trigger commissioning in 2015 was limited due to the fast rise of the LHC luminosity (compared to Run 1), several backup triggers, which contribute additional rate, were implemented in the menu in addition to the primary physics triggers. This is the case for electron, *b*-jet and $E_{\text{T}}^{\text{miss}}$  triggers, which are discussed in later sections of the paper.

### Event streaming

**Fig. 7:**
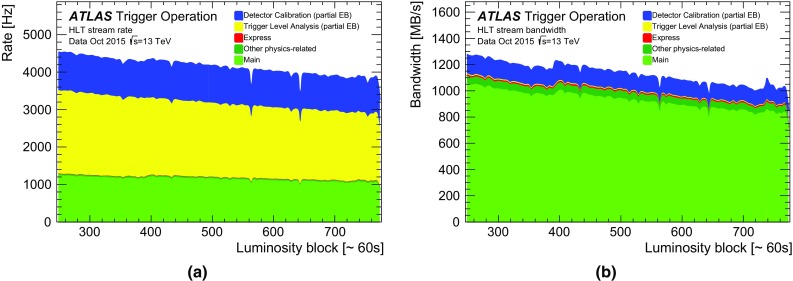
**a** HLT stream rates and **b** bandwidth during an LHC fill in October 2015 with a peak luminosity of $4.5\times 10^{33}$ cm$^{-2}$ s$^{-1}$. Partial Event Building (partial EB) streams only store relevant subdetector data and thus have smaller event sizes. The other physics-related streams contain events with special readout settings and are used to overlay with MC events to simulate pile-up

Events accepted by the HLT are written into separate data *streams*. Events for physics analyses are sent to a single *Main* stream replacing the three separate physics streams (*Egamma*, *Muons*, *JetTauEtMiss*) used in Run 1. This change reduces event duplication, thus reducing storage and CPU resources required for reconstruction by roughly 10%. A small fraction of these events at a rate of 10 to 20 Hz are also written to an *Express* stream that is reconstructed promptly offline and used to provide calibration and data quality information prior to the reconstruction of the full *Main* stream, which typically happens 36 h after the data are taken. In addition, there are about twenty additional streams for calibration, monitoring and detector performance studies. To reduce event size, some of these streams use partial event building (partial EB), which writes only a predefined subset of the ATLAS detector data per event. For Run 2, events that contain only HLT reconstructed objects, but no ATLAS detector data, can be recorded to a new type of stream. These events are of very small size, allowing recording at high rate. These streams are used for calibration purposes and *Trigger-Level Analysis* as described in Sect. 6.4.4. Figure 7 shows typical HLT stream rates and bandwidth during an LHC fill.

Events that cannot be properly processed at the HLT or have other DAQ-related problems are written to dedicated *debug* streams. These events are reprocessed offline with the same HLT configuration as used during data-taking and accepted events are stored into separate data sets for use in physics analyses. In 2015, approximately 339,000 events were written to debug streams. The majority of them (${\sim } 90\%$) are due to online processing timeouts that occur when the event cannot be processed within 2–3 min. Long processing times are mainly due to muon algorithms processing events with a large number of tracks in the muon spectrometer (e.g. due to jets not contained in the calorimeter). During the debug stream reprocessing, 330,000 events were successfully processed by the HLT of which about 85% were accepted. The remaining 9000 events could not be processed due to data integrity issues.

### HLT processing time

**Fig. 8:**
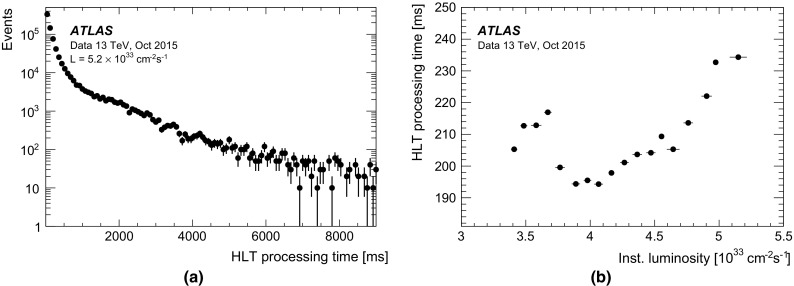
**a** HLT processing time distribution per event for an instantaneous luminosity of $5.2\times 10^{33}$ cm$^{-2}$ s$^{-1}$ and average pile-up $\langle \mu \rangle =15$ and **b** mean HLT processing time as a function of the instantaneous luminosity

The HLT processing time per event is mainly determined by the trigger menu and the number of pile-up interactions. The HLT farm CPU utilisation depends on the L1 trigger rate and the average HLT processing time. Figure 8 shows (a) the HLT processing time distribution for the highest luminosity run in 2015 with a peak luminosity of $5.2\times 10^{33}$ cm$^{-2}$ s$^{-1}$ and (b) the average HLT processing time as a function of the instantaneous luminosity. At the highest luminosity point the average event processing time was approximately 235 ms. An L1 rate of 80 kHz corresponds to an average utilisation of 67% of a farm with 28,000 available CPU cores. About 40, 35 and 15% of the processing time are spent on inner detector tracking, muon spectrometer reconstruction and calorimeter reconstruction, respectively. The muon reconstruction time is dominated by the large rate of low-$p_{\text{T}}$ *B*-physics triggers. The increased processing time at low luminosities observed in Fig. 8b is due to additional triggers being enabled towards the end of an LHC fill to take advantage of the available CPU and bandwidth resources. Moreover, trigger prescale changes are made throughout the run giving rise to some of the observed features in the curve. The clearly visible scaling with luminosity is due to the pileup dependence of the processing time. It is also worth noting that the processing time cannot naively be scaled to higher luminosities as the trigger menu changes significantly in order to keep the L1 rate below or at 100 kHz.

### Trigger menu for special data-taking conditions

Special trigger menus are used for particular data-taking conditions and can either be required for collecting a set of events for dedicated measurements or due to specific LHC bunch configurations. In the following, three examples of dedicated menus are given: menu for low number of bunches in the LHC, menu for collecting enhanced minimum-bias data for trigger rate predictions and menu during beam separation scans for luminosity calibration (van der Meer scans).

**Fig. 9:**
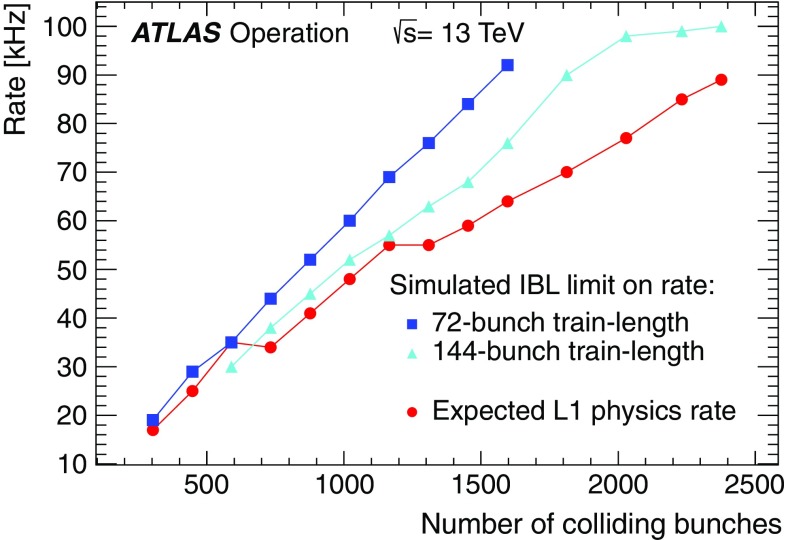
Simulated limits on the L1 trigger rate due to the IBL fixed frequency veto for two different filling schemes and the expected maximum L1 rate from rate predictions. The steps in the latter indicate a change in the prescale strategy. The simulated rate limit is confirmed with experimental tests. The rate limit is higher for the 72-bunch train configuration since the bunches are more equally spread across the LHC ring. The rate limitation was only crucial for the low luminosity phase, where the required physics L1 rate was higher than the limit imposed by the IBL veto. The maximum number of colliding bunches in 2015 was 2232

When the LHC contains a low number of bunches (and thus few bunch trains), care is needed not to trigger at resonant frequencies that could damage the wire bonds of the IBL or SCT detectors, which reside in the magnetic field. The dangerous resonant frequencies are between 9 and 25 kHz for the IBL and above 100 kHz for the SCT detector. To avoid this risk, both detectors have implemented in the readout firmware a so-called fixed frequency veto that prevents triggers falling within a dangerous frequency range [23]. The IBL veto poses the most stringent limit on the acceptable L1 rate in this LHC configuration. In order to provide trigger menus appropriate to each LHC configuration during the startup phase, the trigger rate has been estimated after simulating the effect of the IBL veto. Figure 9 shows the simulated IBL rate limit for two different bunch configurations and the expected L1 trigger rate of the nominal physics trigger menu. At a low number of bunches the expected L1 trigger rate exceeds slightly the allowed L1 rate imposed by the IBL veto. In order not to veto important physics triggers, the required rate reduction was achieved by reducing the rate of supporting triggers.

Certain applications such as trigger algorithm development, rate predictions and validation require a data set that is minimally biased by the triggers used to select it. This special data set is collected using the enhanced minimum-bias trigger menu, which consists of all primary lowest-$p_{\text{T}}$ L1 triggers with increasing $p_{\text{T}}$ threshold and a random trigger for very high cross-section processes. This trigger menu can be enabled in addition to the regular physics menu and records events at 300 Hz for a period of approximately one hour to obtain a data set of around one million events. Since the correlations between triggers are preserved, per-event weights can be calculated and used to convert the sample into a zero-bias sample, which is used for trigger rate predictions during the development of new triggers [24]. This approach requires a much smaller total number of events than a true zero-bias data set.

During van der Meer scans [25], which are performed by the LHC to allow the experiments to calibrate their luminosity measurements, a dedicated trigger menu is used. ATLAS uses several luminosity algorithms (see Ref. [26]) amongst which one relies on counting tracks in the ID. Since the different LHC bunches do not have the exact same proton density, it is beneficial to sample a few bunches at the maximum possible rate. For this purpose, a minimum-bias trigger selects events for specific LHC bunches and uses partial event building to read out only the ID data at about 5 kHz for five different LHC bunches.

## High-level trigger reconstruction

After L1 trigger acceptance, the events are processed by the HLT using finer-granularity calorimeter information, precision measurements from the MS and tracking information from the ID, which are not available at L1. As needed, the HLT reconstruction can either be executed within RoIs identified at L1 or for the full detector. In both cases the data is retrieved on demand from the readout system. As in Run 1, in order to reduce the processing time, most HLT triggers use a two-stage approach with a fast first-pass reconstruction to reject the majority of events and a slower precision reconstruction for the remaining events. However, with the merging of the previously separate L2 and EF farms, there is no longer a fixed bandwidth or rate limitation between the two steps. The following sections describe the main reconstruction algorithms used in the HLT for inner detector, calorimeter and muon reconstruction.

### Inner detector tracking

For Run 1 the ID tracking in the trigger consisted of custom tracking algorithms at L2 and offline tracking algorithms adapted for running in the EF. The ID trigger was redesigned for Run 2 to take advantage of the merged HLT and include information from the IBL. The latter significantly improves the tracking performance and in particular the impact parameter resolution [7]. In addition, provision was made for the inclusion of FTK tracks once that system becomes available later in Run 2.

#### Inner detector tracking algorithms

The tracking trigger is subdivided into *fast tracking* and *precision tracking* stages. The fast tracking consists of trigger-specific pattern recognition algorithms very similar to those used at L2 during Run 1, whereas the precision stage relies heavily on offline tracking algorithms. Despite similar naming the fast tracking as described here is not related to the FTK hardware tracking that will only become available during 2017. The tracking algorithms are typically configured to run within an RoI identified by L1. The offline tracking was reimplemented in LS1 to run three times faster than in Run 1, making it more suitable to use in the HLT. To reduce CPU usage even further, the offline track-finding is seeded by tracks and space-points identified by the fast tracking stage.

#### Inner detector tracking performance

**Fig. 10:**
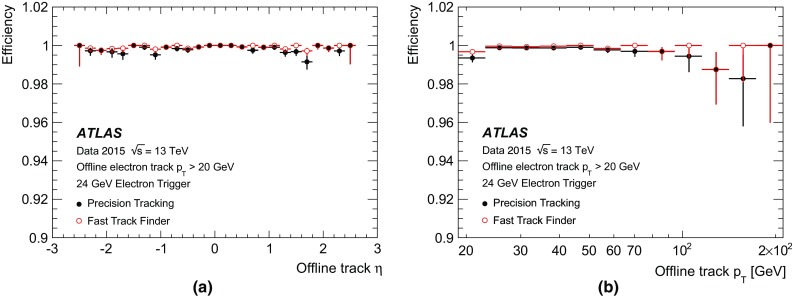
The ID tracking efficiency for the 24 $\text{GeV}$ electron trigger is shown as a function of the **a** $\eta$ and **b** $p_{\text{T}}$ of the track of the offline electron candidate. Uncertainties based on Bayesian statistics are shown

The tracking efficiency with respect to offline tracks has been determined for electrons and muons. The reconstructed tracks are required to have at least two (six) pixel (SCT) clusters and lie in the region $|\eta |<2.5$. The closest trigger track within a cone of size $\Delta R = \sqrt{(\Delta \eta )^2+(\Delta \phi )^2} = 0.05$ of the offline reconstructed track is selected as the matching trigger track.

Figure 10 shows the tracking efficiency for the 24 $\text{GeV}$ medium electron trigger (see Sect. 6.2) as a function of the $\eta$ and of the $p_{\text{T}}$ of the offline track. The tracking efficiency is measured with respect to offline tracks with $p_{\text{T}} >{20}\,{\text{GeV}}$ for tight offline electron candidates from the 24 $\text{GeV}$ electron support trigger, which does not use the trigger tracks in the selection, but is otherwise identical to the physics trigger. The efficiencies of the fast track finder and precision tracking exceed 99% for all pseudorapidities. There is a small efficiency loss at low $p_{\text{T}}$ due to bremsstrahlung energy loss by electrons.

**Fig. 11:**
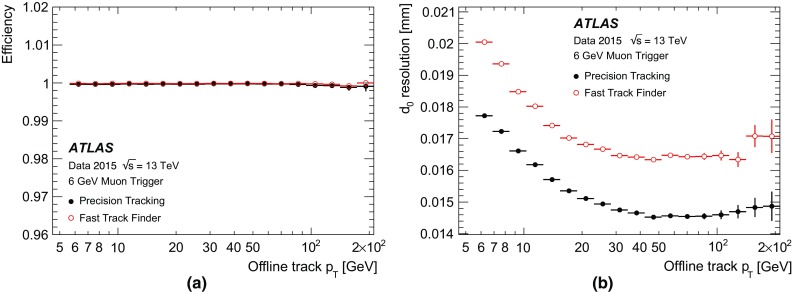
The ID tracking performance for the 6 $\text{GeV}$ muon trigger; **a** efficiency as a function of the offline reconstructed muon $p_{\text{T}}$, **b** the resolution of the transverse impact parameter, $d_{0}$ as a function of the offline reconstructed muon $p_{\text{T}}$. Uncertainties based on Bayesian statistics are shown

Figure 11a shows the tracking performance of the ID trigger for muons with respect to loose offline muon candidates with $p_{\text{T}} >{6}\,{\text{GeV}}$ selected by the 6 $\text{GeV}$ muon support trigger as a function of the offline muon transverse momentum. The efficiency is significantly better than 99% for all $p_{\text{T}}$ for both the fast and precision tracking. Shown in Fig. 11b is the resolution of the transverse track impact parameter with respect to offline as a function of the offline muon $p_{\text{T}}$. The resolution in the fast (precision) tracking is better than 17 $\upmu$m (15 $\upmu$m) for muon candidates with offline $p_{\text{T}} > {20}\,{\text{GeV}}$.

#### Multiple stage tracking

For the hadronic tau and *b*-jet triggers, tracking is run in a larger RoI than for electrons or muons. To limit CPU usage, multiple stage track reconstruction was implemented.

**Fig. 12:**
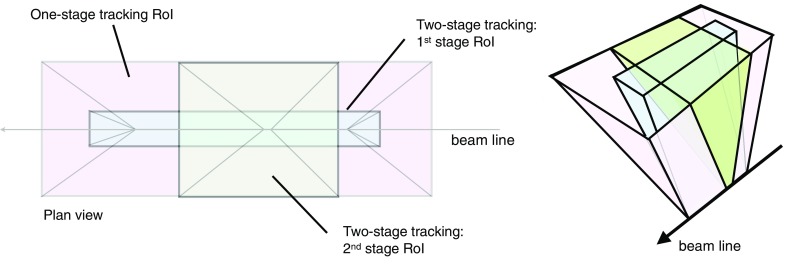
A schematic illustrating the RoIs from the single-stage and two-stage tau lepton trigger tracking, shown in plan view (x–z plane) along the transverse direction and in perspective view. The z-axis is along the beam line. The combined tracking volume of the 1st and 2nd stage RoI in the two-stage tracking approach is significantly smaller than the RoI in the one-stage tracking scheme

A two-stage processing approach was implemented for the hadronic tau trigger. First, the leading track and its position along the beamline are determined by executing fast tracking in an RoI that is fully extended along the beamline ($|z|<{225}$ mm) but narrow (0.1) in both $\eta$ and $\phi$. (See the blue-shaded region in Fig. 12.) Using this position along the beamline, the second stage reconstructs all tracks in an RoI that is larger (0.4) in both $\eta$ and $\phi$ but limited to $|\Delta z|<{10}$ mm with respect to the leading track. (See the green shaded region in Fig. 12.) At this second stage, fast tracking is followed by precision tracking. For evaluation purposes, the tau lepton signatures can also be executed in a single-stage mode, running the fast track finder followed by the precision tracking in an RoI of the full extent along the beam line and in eta and phi.

**Fig. 13:**
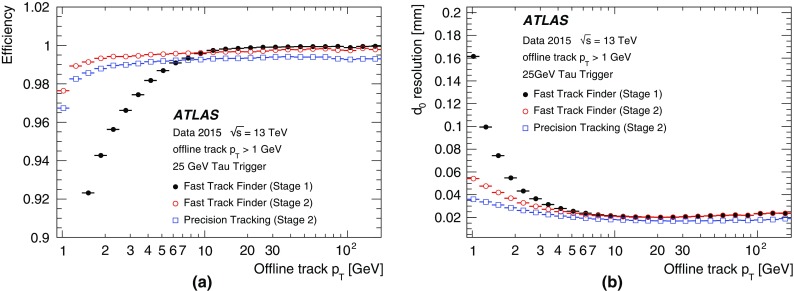
The ID trigger tau tracking performance with respect to offline tracks from very loose tau candidates with $p_{\text{T}} >{1}\,{\text{GeV}}$ from the 25 $\text{GeV}$ tau trigger; **a** the efficiency as a function of the offline reconstructed tau track $p_{\text{T}}$, **b** the resolution of the transverse impact parameter, $d_{0}$ as a function of the offline reconstructed tau track $p_{\text{T}}$. The offline reconstructed tau daughter tracks are required to have $p_{\text{T}} >{1}\,{\text{GeV}}$, lie in the region $|\eta |<2.5$ and have at least two pixel clusters and at least six SCT clusters. The closest matching trigger track within a cone of size $\Delta R = 0.05$ of the offline track is selected as the matching trigger track

Figure 13 shows the performance of the tau two-stage tracking with respect to the offline tau tracking for tracks with $p_{\text{T}} >{1}\,{\text{GeV}}$ originating from decays of offline tau lepton candidates with $p_{\text{T}} > {25}\,{\text{GeV}}$, but with very loose track matching in $\Delta R$ to the offline tau candidate. Figure 13a shows the efficiency of the fast tracking from the first and second stages, together with the efficiency of the precision tracking for the second stage. The second-stage tracking efficiency is higher than 96% everywhere, and improves to better than 99% for tracks with $p_{\text{T}} >{2}\,{\text{GeV}}$. The efficiency of the first-stage fast tracking has a slower turn-on, rising from 94% at 2 $\text{GeV}$ to better than 99% for $p_{\text{T}} >{5}\,{\text{GeV}}$. This slow turn-on arises due to the narrow width ($\Delta \phi <0.1$) of the first-stage RoI and the loose tau selection that results in a larger fraction of low-$p_{\text{T}}$ tracks from tau candidates that bend out of the RoI (and are not reconstructed) compared to a wider RoI. The transverse impact parameter resolution with respect to offline for loosely matched tracks is seen in Fig. 13b and is around 20 $\upmu$m for tracks with $p_{\text{T}} >{10}\,{\text{GeV}}$ reconstructed by the precision tracking. The tau selection algorithms based on this two-stage tracking are presented in Sect. 6.5.1.

**Fig. 14:**
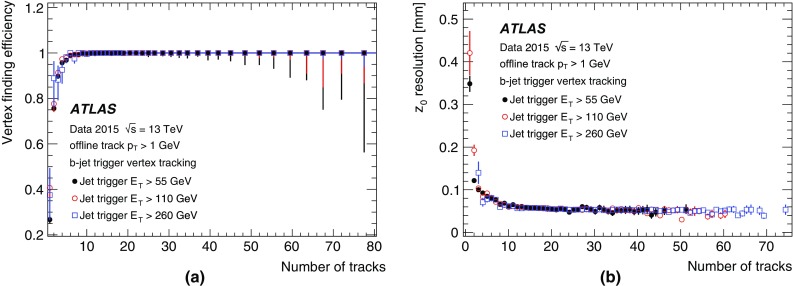
The trigger performance for primary vertices in the *b*-jet signatures for 55, 110 and 260$\text{GeV}$jet triggers; **a** the vertexing efficiency as a function of the number of offline tracks within the jets used for the vertex tracking, **b** the resolution in *z* of the vertex with respect to the offline vertex position as a function of the number of offline tracks from the offline vertex

For *b*-jet tracking a similar multi-stage tracking strategy was adopted. However, in this case the first-stage vertex tracking takes all jets identified by the jet trigger with $E_{\text{T}} >{30}\,{\text{GeV}}$ and reconstructs tracks with the fast track finder in a narrow region in $\eta$ and $\phi$ around the jet axis for each jet, but with $|z|<{225}$ mm along the beam line. Following this step, the primary vertex reconstruction [27] is performed using the tracks from the fast tracking stage. This vertex is used to define wider RoIs around the jet axes, with $|\Delta \eta |<0.4$ and $|\Delta \phi |<0.4$ but with $|\Delta z|<{20}$ mm relative to the primary vertex *z* position. These RoIs are then used for the second-stage reconstruction that runs the fast track finder in the wider $\eta$ and $\phi$ regions followed by the precision tracking, secondary vertexing and *b*-tagging algorithms.

The performance of the primary vertexing in the *b*-jet vertex tracking can be seen in Fig. 14a, which shows the vertex finding efficiency with respect to offline vertices in jet events with at least one jet with transverse energy above 55, 110, or 260 $\text{GeV}$ and with no additional *b*-tagging requirement. The efficiency is shown as a function of the number of offline tracks with $p_{\text{T}} >{1}\,{\text{GeV}}$ that lie within the boundary of the wider RoI (defined above) from the selected jets. The efficiency rises sharply and is above 90% for vertices with three or more tracks, and rises to more than 99.5% for vertices with five or more tracks. The resolution in *z* with respect to the offline *z* position as shown in Fig. 14b is better than 100 $\upmu$m for vertices with two or more offline tracks and improves to 60 $\upmu$m for vertices with ten or more offline tracks.

#### Inner detector tracking timing

**Fig. 15:**
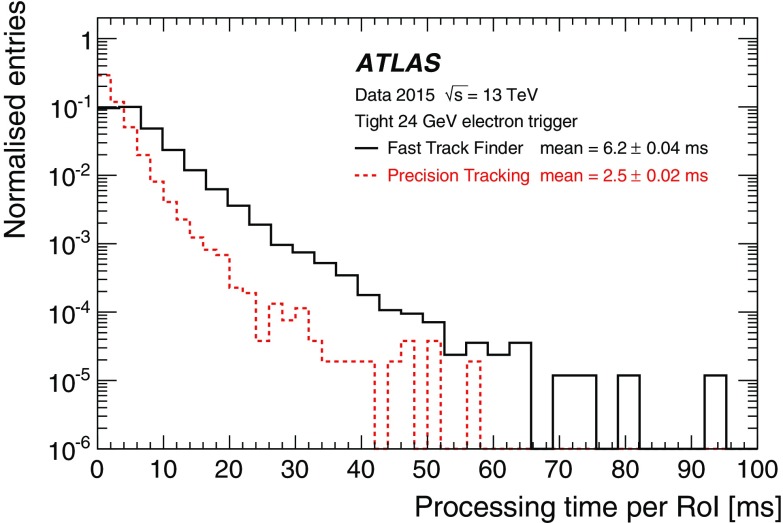
The CPU processing time for the fast and precision tracking per electron RoI for the 24 $\text{GeV}$ electron trigger. The precision tracking is seeded by the tracks found in the fast tracking stage and hence requires less CPU time

The timing of the fast tracking and precision tracking stages of the electron trigger executed per RoI can be seen in Fig. 15 for events passing the 24 $\text{GeV}$ electron trigger. The fast tracking takes on average 6.2 ms per RoI with a tail at the per-mille level at around 60 ms. The precision tracking execution time has a mean of 2.5 ms and a tail at the per-mille level of around 20 ms. The precision tracking is seeded by the tracks found in the fast tracking stage and hence requires less CPU time.

**Fig. 16:**
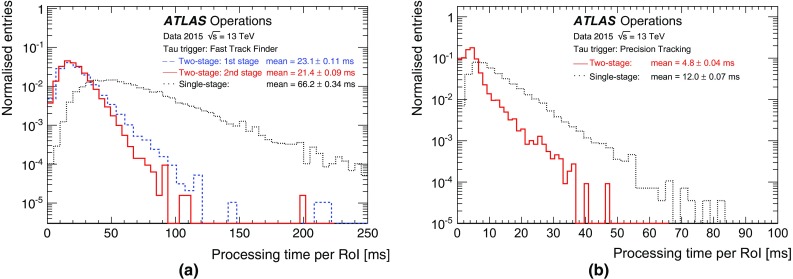
The ID trigger tau tracking processing time for **a** the fast track finder and **b** the precision tracking comparing the single-stage and two-stage tracking approach

The time taken by the tau tracking in both the single-stage and two-stage variants is shown in Fig. 16. Figure 16a shows the processing times per RoI for fast tracking stages: individually for the first and second stages of the two-stage tracking, and separately for the single-stage tracking with the wider RoI in $\eta$, $\phi$ and *z*. The fast tracking in the single-stage tracking has a mean execution time of approximately 66 ms, with a very long tail. In contrast, the first-stage tracking with an RoI that is wide only in the *z* direction has a mean execution time of 23 ms, driven predominantly by the narrower RoI width in $\phi$. The second-stage tracking, although wider in $\eta$ and $\phi$, takes only 21 ms on average because of the significant reduction in the RoI *z*-width along the beam line. Figure 16b shows a comparison of the processing time per RoI for the precision tracking. The two-stage tracking executes faster, with a mean of 4.8 ms compared to 12 ms for the single-stage tracking. Again, this is due to the reduction in the number of tracks to be processed from the tighter selection in *z* along the beam line.

### Calorimeter reconstruction

A series of reconstruction algorithms are used to convert signals from the calorimeter readout into objects, specifically cells and clusters, that then serve as input to the reconstruction of electron, photon, tau, and jet candidates and the reconstruction of $E_{\text{T}}^{\text{miss}}$. These cells and clusters are also used in the determination of the shower shapes and the isolation properties of candidate particles (including muons), both of which are later used as discriminants for particle identification and the rejection of backgrounds. The reconstruction algorithms used in the HLT have access to full detector granularity and thus allow improved accuracy and precision in energy and position measurements with respect to L1.

#### Calorimeter algorithms

The first stage in the reconstruction involves unpacking the data from the calorimeter. The unpacking can be done in two different ways: either by unpacking only the data from within the RoIs identified at L1 or by unpacking the data from the full calorimeter. The RoI-based approach is used for well-separated objects (e.g. electron, photon, muon, tau), whereas the full calorimeter reconstruction is used for jets and global event quantities (e.g. $E_{\text{T}}^{\text{miss}}$). In both cases the raw unpacked data is then converted into a collection of cells. Two different clustering algorithms are used to reconstruct the clusters of energy deposited in the calorimeter, the sliding-window and the topo-clustering algorithms [28]. While the latter provides performance closer to the offline reconstruction, it is also significantly slower (see Sect. 5.2.3).

The sliding-window algorithm operates on a grid in which the cells are divided into projective towers. The algorithm scans this grid and positions the window in such a way that the transverse energy contained within the window is the local maximum. If this local maximum is above a given threshold, a cluster is formed by summing the cells within a rectangular clustering window. For each layer the barycentre of the cells within that layer is determined, and then all cells within a fixed window around that position are included in the cluster. Although the size of the clustering window is fixed, the central position of the window may vary slightly at each calorimeter layer, depending on how the cell energies are distributed within them.

The topo-clustering algorithm begins with a seed cell and iteratively adds neighbouring cells to the cluster if their energies are above a given energy threshold that is a function of the expected root-mean-square (RMS) noise ($\sigma$). The seed cells are first identified as those cells that have energies greater than 4$\sigma$. All neighbouring cells with energies greater than 2$\sigma$ are then added to the cluster and, finally, all the remaining neighbours to these cells are also added. Unlike the sliding-window clusters, the topo-clusters have no predefined shape, and consequently their size can vary from cluster to cluster.

The reconstruction of candidate electrons and photons uses the sliding-window algorithm with rectangular clustering windows of size $\Delta \eta \times \Delta \phi$ = 0.075 $\times$ 0.175 in the barrel and 0.125 $\times$ 0.125 in the end-caps. Since the magnetic field bends the electron trajectory in the $\phi$ direction, the size of the window is larger in that coordinate in order to contain most of the energy. The reconstruction of candidate taus and jets and the reconstruction of $E_{\text{T}}^{\text{miss}}$ all use the topo-clustering algorithm. For taus the topo-clustering uses a window of 0.8 $\times$ 0.8 around each of the tau RoIs identified at L1. For jets and $E_{\text{T}}^{\text{miss}}$, the topo-clustering is done for the full calorimeter. In addition, the $E_{\text{T}}^{\text{miss}}$ is also determined based on the cell energies across the full calorimeter (see Sect. 6.6).

#### Calorimeter algorithm performance

**Fig. 17:**
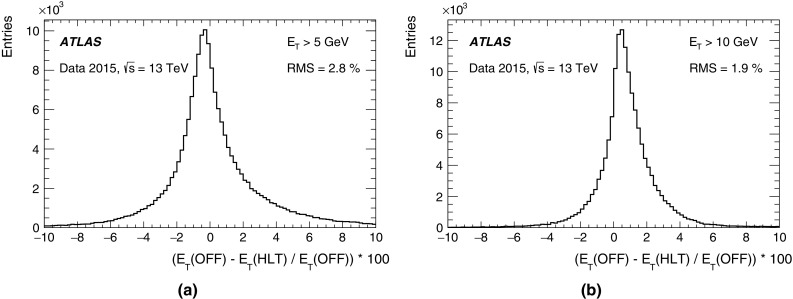
The relative differences between the online and offline $E_{\text{T}}$ for **a** sliding-window clusters and **b** topo-clusters. Online and offline clusters are matched within $\Delta R$ < 0.001. The distribution for the topo-clusters was obtained from the RoI-based topo-clustering algorithm that is used for online tau reconstruction

The harmonisation between the online and offline algorithms in Run 2 means that the online calorimeter performance is now much closer to the offline performance. The $E_{\text{T}}$ resolutions of the sliding-window clusters and the topo-clusters with respect to their offline counterparts are shown in Fig. 17. The $E_{\text{T}}$ resolution of the sliding-window clusters is 3% for clusters above 5 $\text{GeV}$, while the $E_{\text{T}}$ resolution of the topo-clustering algorithm is 2% for clusters above 10 $\text{GeV}$. The slight shift in cell energies between the HLT and offline is due to the fact that out-of-time pile-up effects were not corrected in the online reconstruction, resulting in slightly higher reconstructed cell energies in the HLT (this was changed for 2016). In addition, the topo-cluster based reconstruction shown in Fig. 17b suffered from a mismatch of some calibration constants between online and offline during most of 2015, resulting in a shift towards lower HLT cell energies.

#### Calorimeter algorithm timing

**Fig. 18:**
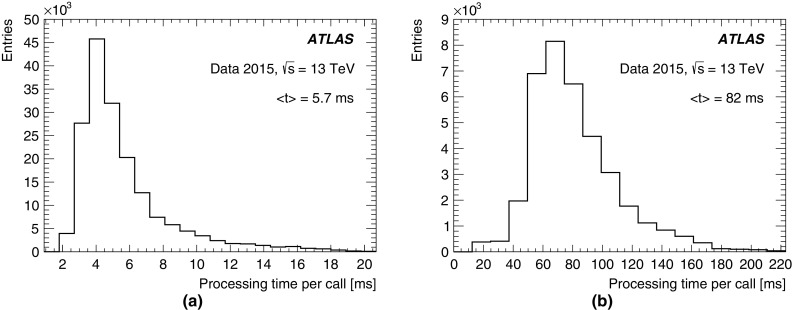
The distributions of processing times for the topo-clustering algorithm executed **a** within an RoI and **b** on the full calorimeter. The processing times within an RoI are obtained from tau RoIs with a size of $\Delta \eta \times \Delta \phi =0.8\times 0.8$

Due to the optimisation of the offline clustering algorithms during LS1, offline clustering algorithms can be used in the HLT directly after the L1 selection. At the data preparation stage, a specially optimised infrastructure with a memory caching mechanism allows very fast unpacking of data, even from the full calorimeter, which comprises approximately 187,000 cells. The mean processing time for the data preparation stage is 2 ms per RoI and 20 ms for the full calorimeter, and both are roughly independent of pile-up. The topo-clustering, however, requires a fixed estimate of the expected pile-up noise (cell energy contributions from pile-up interactions) in order to determine the cluster-building thresholds and, when there is a discrepancy between the expected pile-up noise and the actual pile-up noise, the processing time can show some dependence on the pile-up conditions. The mean processing time for the topo-clustering is 6 ms per RoI and 82 ms for the full calorimeter. The distributions of the topo-clustering processing times are shown in Fig. 18a for an RoI and Fig. 18b for the full calorimeter. The RoI-based topo-clustering can run multiple times if there is more than one RoI per event. The topo-clustering over the full calorimeter runs at most once per event, even if the event satisfied both jet and $E_{\text{T}}^{\text{miss}}$ selections at L1. The mean processing time of the sliding window clustering algorithm is not shown but is typically less than 2.5 ms per RoI.

### Tracking in the muon spectrometer

Muons are identified at the L1 trigger by the spatial and temporal coincidence of hits either in the RPC or TGC chambers within the rapidity range of $|\eta |<2.4$. The degree of deviation from the hit pattern expected for a muon with infinite momentum is used to estimate the $p_{\text{T}}$ of the muon with six possible thresholds. The HLT receives this information together with the RoI position and makes use of the precision MDT and CSC chambers to further refine the L1 muon candidates.

#### Muon tracking algorithms

The HLT muon reconstruction is split into *fast* (trigger specific) and *precision* (close to offline) reconstruction stages, which were used during Run 1 at L2 and EF, respectively.

In the fast reconstruction stage, each L1 muon candidate is refined by including the precision data from the MDT chambers in the RoI defined by the L1 candidate. A track fit is performed using the MDT drift times and positions, and a $p_{\text{T}}$ measurement is assigned using lookup tables, creating *MS-only* muon candidates. The MS-only muon track is back-extrapolated to the interaction point using the offline track extrapolator (based on a detailed detector description instead of the lookup-table-based approach used in Run 1) and combined with tracks reconstructed in the ID to form a *combined* muon candidate with refined track parameter resolution.

In the precision reconstruction stage, the muon reconstruction starts from the refined RoIs identified by the fast stage, reconstructing segments and tracks using information from the trigger and precision chambers. As in the fast stage, muon candidates are first formed by using the muon detectors (MS-only) and are subsequently combined with ID tracks leading to combined muons. If no matching ID track can be found, combined muon candidates are searched for by extrapolating ID tracks to the MS. This latter *inside-out* approach is slower and hence only used if the *outside-in* search fails. It recovers about 1–5% of the muons, most of them at low $p_{\text{T}}$.

The combined muon candidates are used for the majority of the muon triggers. However, MS-only candidates are used for specialised triggers that cannot rely on the existence of an ID track, e.g. triggers for long-lived particles that decay within the ID volume.

#### Muon tracking performance

**Fig. 19:**
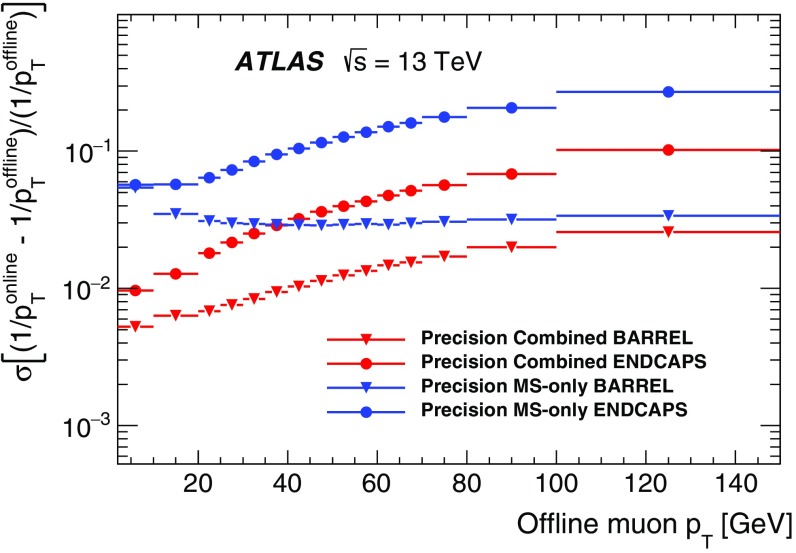
Width of the residuals for inverse-$p_{\text{T}}$ as a function of offline muon $p_{\text{T}}$ for the precision MS-only and combined algorithms in the barrel ($|\eta |<1.05$) and end-caps ($1.0<|\eta |<2.4$)

**Fig. 20:**
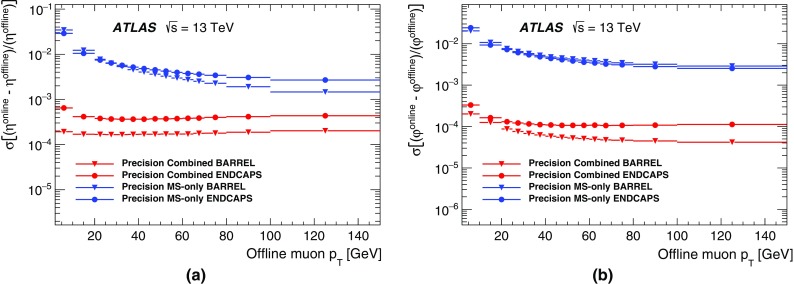
Width of the residuals as a function of the offline muon $p_{\text{T}}$ for **a** $\eta$ and **b** $\phi$ for the precision MS-only and combined algorithms in the barrel ($|\eta |<1.05$) and end-caps ($1.0<|\eta |<2.4$)

Comparisons between online and offline muon track parameters using $Z\rightarrow \mu \mu$ candidate events are presented in this section while muon trigger efficiencies are described in Sect. 6.3. Distributions of the residuals between online and offline track parameters ($1/p_{\text{T}}$, $\eta$ and $\phi$) are constructed in bins of $p_{\text{T}}$ and two subsequent Gaussian fits are performed on the core of the distribution to extract the widths, $\sigma$, of the residual distributions as a function of $p_{\text{T}}$. The inverse-$p_{\text{T}}$ residual widths, $\sigma ((1/p_{\text{T}}^{\mathrm{online}}-1/p_{\text{T}}^{\mathrm{offline}})/(1/p_{\text{T}}^{\mathrm{offline}}))$, are shown in Fig. 19 as a function of the offline muon $p_{\text{T}}$ for the precision MS-only and precision combined reconstruction. The resolution for combined muons is better than the resolution for MS-only muons due to the higher precision of the ID track measurements, especially at low $p_{\text{T}}$. As the tracks become closer to straight lines at high $p_{\text{T}}$, it becomes more difficult to precisely measure the $p_{\text{T}}$ of both the MS and ID tracks, and hence the resolution degrades. The $p_{\text{T}}$ resolution for low-$p_{\text{T}}$ MS-only muons is degraded when muons in the barrel are bent out of the detector before traversing the entire muon spectrometer. The resolution is generally better in the barrel than in the end-caps due to the difference in detector granularity. The $\eta$ residual widths, $\sigma (\eta^{\mathrm{online}}-\eta^{\mathrm{offline}})$, and $\phi$ residual widths, $\sigma (\phi^{\mathrm{online}}-\phi^{\mathrm{offline}})$, are shown as a function of $p_{\text{T}}$ in Fig. 20 for both the MS-only and combined algorithms. As the trajectories are straighter at high $p_{\text{T}}$, the precision of their position improves and so the spatial resolution decreases with $p_{\text{T}}$. Good agreement between track parameters calculated online and offline is observed.

#### Muon tracking timing

**Fig. 21:**
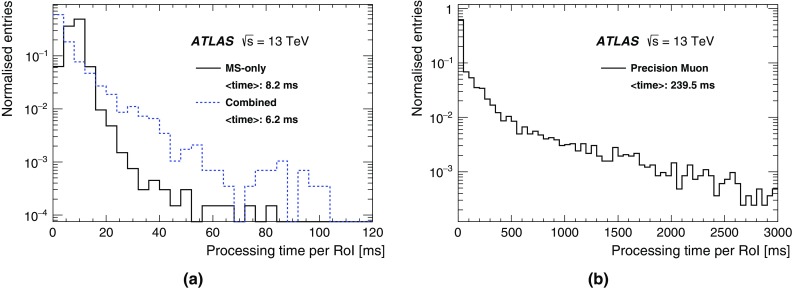
Processing times per RoI for the **a** fast MS-only and fast combined algorithms and **b** precision muon-finding algorithm. The time for the combined algorithm includes only the time for the ID–MS combination and not the tracking itself. The mean time of each algorithm is indicated in the legend. The large number of entries in the first bin in **b** is due to algorithm caching [29]

Figure 21 shows the processing times per RoI for the (a) fast MS-only and fast combined algorithms and (b) precision muon algorithm. The large time difference between the fast and precision algorithms, with the precision reconstruction using too much time to be run by itself at the full L1 muon trigger rate, motivates the need for a two-stage reconstruction.

## Trigger signature performance

The following sections describe the different selection criteria placed upon the reconstructed objects described in Sect. 5 in order to form individual trigger signatures that identify leptons, hadrons, and global event quantities such as $E_{\text{T}}^{\text{miss}}$. For each case the primary triggers used during 2015 are listed together with their output rate and performance. Where possible the trigger efficiency measured in data is compared with MC simulation. The following methods are used to derive an unbiased measurement of the trigger efficiency:

- *Tag-and-probe method*, which uses a sample of offline-selected events that contain a pair of related objects reconstructed offline, such as electrons from a $Z \rightarrow ee$ decay, where one has triggered the event and the other one is used to measure the trigger efficiency;
- *Bootstrap method*, where the efficiency of a higher trigger threshold is determined using events triggered by a lower threshold.

Trigger efficiencies are computed with respect to an offline-selected data sample. The ratio of the measured trigger efficiency to the simulated one is used as a correction factor in physics analyses. Unless otherwise specified, performance studies use good-quality data corresponding to an integrated luminosity of 3.2 fb$^{-1}$ collected during 2015 with a bunch-spacing of 25 ns. Trigger rates shown in the following sections are usually extracted from multiple data-taking runs to cover the maximum range in instantaneous luminosity. Due to different beam and detector conditions between runs, this can result in slightly different trigger rates for nearby luminosity values.

### Minimum-bias and forward triggers

Studies of the total cross-section, hadronisation, diffraction, hadrons containing strange quarks and other non-perturbative properties of *pp* interactions require the use of a high-efficiency trigger for selecting all inelastic interactions that result in particle production within the detector. The MBTS minimum-bias trigger is highly efficient, even for events containing only two charged particles with $p_{\text{T}} >{100}\,{\text{MeV}}$ and $|\eta | < 2.5$.

The primary minimum-bias and high-multiplicity data set at $\sqrt{s}={13}\,{\text{TeV}}$ was recorded in June 2015. The average pile-up $\left<\mu \right>$ varied between 0.003 and 0.03, and the interaction rate had a maximum of about 15 kHz. More than 200 million interactions were recorded during a one-week data-taking period. Most of the readout bandwidth was dedicated to the loosest L1_MBTS_1 trigger (described below) recording events at 1.0 to 1.5 kHz on average.

#### Reconstruction and selection

The MBTS are used as the primary L1 hardware triggers for recording inelastic events with minimum bias, as reported in Refs. [30, 31]. The plastic scintillation counters composing the system were replaced during LS1 and consist of two planes of twelve counters, each plane formed of an inner ring of eight counters and an outer ring of four counters. These rings are sensitive to charged particles in the interval $2.07\,<\,|\eta |\,<\,3.86$. Each counter is connected to a photomultiplier tube and provides a fast trigger via a constant fraction discriminator and is read out through the Tile calorimeter data acquisition system.

The MBTS triggers require a certain multiplicity of counters to be above threshold in a bunch-crossing with colliding beams. The L1_MBTS_1 and L1_MBTS_2 triggers require any one or two of the 24 counters to be above threshold, respectively. The coincidence of two hits in the latter suppresses beam-induced backgrounds from low-energy neutrons and photons. The L1_MBTS_1_1 trigger requires at least one counter to be above threshold in both the $+z$ and $-z$ hemispheres of the detector and is used to seed the high-multiplicity HLT triggers. The same trigger selections are also applied to empty (no beam present) and unpaired (one beam present) beam-crossings to investigate beam-induced backgrounds. No additional HLT selection is applied to L1_MBTS_1 and L1_MBTS_2 triggered events.

The mb_sptrk trigger is used to determine the efficiency of the MBTS. It is seeded using a random trigger on filled bunches and requires at least two reconstructed space-points in the Pixel system and three in the SCT, along with at least one reconstructed track with $p_{\text{T}} > {200}\,{\text{MeV}}$. Studies using MC simulation and a fully unbiased data sample have demonstrated that this control trigger is unbiased with respect to the offline selection.

The primary high-multiplicity trigger (e.g. used in the measurement of two-particle correlations [32]) is mb_sp900_trk60_hmt_L1MBTS_1_1 and requires at least 900 reconstructed space-points in the SCT and at least 60 reconstructed tracks with $p_{\text{T}} > {400}\,{\text{MeV}}$. This higher $p_{\text{T}}$ requirement for the high-multiplicity trigger is compatible with the $p_{\text{T}}$ cut used for physics analysis and reduces the computational complexity of the track-finding algorithms in the HLT to an acceptable level.

#### Trigger efficiencies

**Fig. 22:**
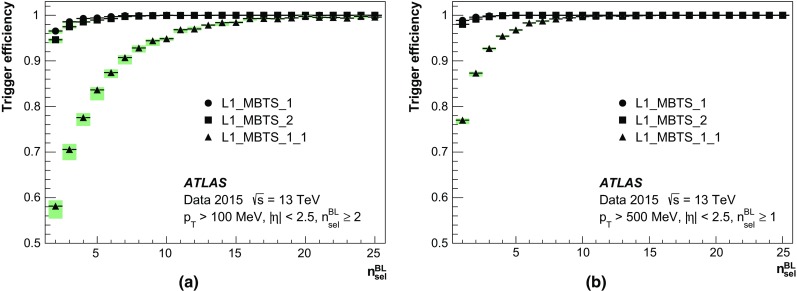
Efficiency of L1_MBTS_1, L1_MBTS_2 and L1_MBTS_1_1 triggers as a function of the number tracks compatible with the beam line for two different transverse momentum requirements **a** $p_{\text{T}} >{100}\,{\text{MeV}}$ and **b** $p_{\text{T}} >{500}\,{\text{MeV}}$. The bands denote the total uncertainty

The MBTS trigger efficiency is defined as the ratio of events passing MBTS trigger, the control trigger (mb_sptrk) and offline selection to events passing the control trigger and offline selection. The efficiency is shown in Fig. 22 for two offline selections as a function of the number of selected tracks compatible in transverse impact parameter ($|d_0|<{1.5}$ mm) with the beam line ($n_{\mathrm{sel}}^{\mathrm{BL}}$) for (a) $p_{\text{T}} > {100}\,{\text{MeV}}$ and (b) $p_{\text{T}} > {500}\,{\text{MeV}}$. The efficiency is close to 95% in the first bin, quickly rising to 100% for L1_MBTS_1 and L1_MBTS_2. The L1_MBTS_1_1 trigger, which requires at least one hit on both sides of the detector, only approaches 100% efficiency for events with around 15 tracks. The primary reason for the lower efficiency of the L1_MBTS_1_1 trigger compared to L1_MBTS_1 or L1_MBTS_2 is that at low multiplicities about 30% of the inelastic events are due to diffractive interactions where usually one proton stays intact and thus particles from the interactions are only produced on one side of the detector. Systematic uncertainties in the trigger efficiency are evaluated by removing the cut on the transverse impact parameter with respect to the beam line from the track selection and applying a longitudinal impact parameter cut with respect to the primary vertex (for events where a primary vertex is reconstructed). This results in a less than 0.1% shift. The difference in response between the two hemispheres is additionally evaluated to be at most 0.12%.

**Fig. 23:**
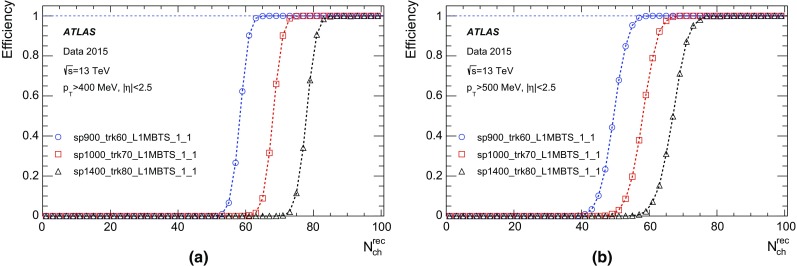
Efficiency of high-multiplicity triggers as a function of the number of tracks compatible with the primary vertex for two different offline transverse momentum requirements **a** $p_{\text{T}} >{400}\,{\text{MeV}}$ and **b** $p_{\text{T}} >{500}\,{\text{MeV}}$. The *curves* represent three different selections on the minimum number of SCT space-points and reconstructed tracks (900/60, 1000/70 and 1400/80)

The L1_MBTS_1 trigger is used as the control trigger for the determination of the efficiency turn-on curves for the high-multiplicity data set. The efficiency is parameterised as a function of the number of offline tracks associated with the primary vertex. Figure 23 shows the efficiency for three different selections of the minimum number of SCT space-points and reconstructed tracks and for two selections of the offline track $p_{\text{T}}$ requirement (above 400 and 500 $\text{MeV}$). In the case of matching offline and trigger $p_{\text{T}}$ selections ($p_{\text{T}} >{400}\,{\text{MeV}}$) shown in Fig. 23a, the triggers are 100% efficient for a value of five tracks above the offline threshold (e.g. trk60 becomes fully efficient for 65 offline tracks). If the offline requirement is raised to 500 $\text{MeV}$ as shown in Fig. 23b, the trigger is 100% efficient for the required number of tracks.

### Electrons and photons

Events with electrons and photons in the final state are important signatures for many ATLAS physics analyses, from SM precision physics, such as Higgs boson, top quark, *W* and *Z* boson properties and production rate measurements, to searches for new physics. Various triggers cover the energy range between a few GeV and several TeV. Low-$E_{\text{T}}$ triggers are used to collect data for measuring the properties of $J/\psi \rightarrow ee$, diphoton or low mass Drell–Yan production. Single-electron triggers with $E_{\text{T}}$ above 24 $\text{GeV}$, dielectron triggers with lower thresholds and diphoton triggers are used for the signal selection in a wide variety of ATLAS physics analyses such as studies of the Higgs boson.

#### Electron and photon reconstruction and selection

At L1 the electron and photon triggers use the algorithms described in Sect. 3.1. The isolation and hadronic leakage veto cuts are not required for EM clusters with transverse energy above 50 $\text{GeV}$.

At the HLT, electron and photon candidates are reconstructed and selected in several steps in order to reject events as fast as possible, thus allowing algorithms which reproduce closely the offline algorithms and require more CPU time to run at a reduced rate later in the trigger sequence. At first, fast calorimeter algorithms build clusters from the calorimeter cells (covering $0.025\times 0.025$ in $\eta \times \phi$ space) within the RoI ($\Delta \eta \times \Delta \phi =0.4\times 0.4$) identified by L1. Since electrons and photons deposit most of their energy in the second layer of the EM calorimeter, this layer is used to find the cell with the largest deposited transverse energy in the RoI. EM calorimeter clusters of size $3\times 7$ in the barrel ($|\eta |<1.4$) and $5\times 5$ in the end-cap ($1.4<|\eta |<2.47$) are used to reconstruct electrons and photons. The identification of electrons and photons is based on the cluster $E_{\text{T}}$ as well as cluster shape parameters such as $R_{\text{had}}$, $R_\eta$ and $E_{\text{ratio}}$,^3^ the latter being used for electron candidates and a few tight photon triggers. Electron candidates are required to have tracks from the fast tracking stage with $p_{\text{T}} > {1}\,{\text{GeV}}$ and to match clusters within $\Delta \eta<$ 0.2.

The second step relies on precise offline-like algorithms. The energy of the clusters is calibrated for electron and photon triggers separately using a multivariate technique where the response of the calorimeter layers is corrected in data and simulation [33]. Precision tracks extrapolated to the second layer of the EM calorimeter are required to match to clusters within $\Delta \eta$ of 0.05 and $\Delta \phi$ of 0.05. Electron identification relies on a multivariate technique using a likelihood (LH) discriminant with three operating points named *loose LH*, *medium LH* and *tight LH*. An additional working point named *very loose LH* is used for supporting triggers. The LH-based identification makes use of variables similar to the cut-based identification employed during Run 1  [2] but has better background rejection for the same signal efficiency. The discriminating variables used offline are also used by the trigger, exploiting the characteristic features of energy deposits in the EM calorimeters (longitudinal and lateral shower shapes), track quality, track-cluster matching, and particle identification by the TRT. All variables are described in Refs. [34, 35]. The composition of the likelihood is the same as in the offline reconstruction with the exception of momentum loss due to bremsstrahlung, $\Delta p/p$, which is not accounted for in the online environment. The photon identification relies only on the cluster shower-shape variables and three working points are also defined: *loose*, *medium* and *tight*.

Not applied during 2015 but foreseen for higher luminosities during Run 2 is an additional requirement on isolation for the lowest-threshold unprescaled single-electron trigger. The isolation parameter is calculated as the sum of the $p_{\text{T}}$ values of all tracks in a cone of size $\Delta R=0.2$ around the electron for tracks with $p_{\text{T}} > {1}\,{\text{GeV}}$ and $|\Delta z_{0} \sin \theta |<$ 0.3, where $\Delta z_0$ is the distance along *z* between the longitudinal impact parameter of the track and the leading track in the RoI. The ratio of this quantity to the EM cluster $E_{\text{T}}$, namely $\sum {p_{\text{T}}}/E_{\text{T}}$, is used to estimate the energy deposited by other particles.

#### Electron and photon trigger menu and rates

The primary L1 and HLT electron and photon triggers used in 2015 are listed in Table 1. The lowest-threshold single-electron trigger (e24_lhmedium_L1EM20VH) applies a 24 $\text{GeV}$ transverse energy threshold and requires the electron to pass medium LH identification requirements. The trigger is seeded by L1_EM20VH, which requires $E_{\text{T}} >{20}\,{\text{GeV}}$, and applies an $E_{\text{T}}$-dependent veto against energy deposited in the hadronic calorimeter behind the electromagnetic cluster of the electron candidate (hadronic veto, denoted by H in the trigger name). The $E_{\text{T}}$ threshold varies slightly as a function of $\eta$ to compensate for passive material in front of the calorimeter (denoted by V in the trigger name). To recover efficiency in the high transverse energy regime, this trigger is complemented by a trigger requiring a transverse energy above 120 $\text{GeV}$ with loose LH identification (e120_lhloose). With a maximum instantaneous luminosity of $5.2\times 10^{33}$ cm$^{-2}$ s$^{-1}$ reached during the 2015 data-taking, the rates of electron triggers could be sustained without the use of additional electromagnetic or track isolation requirements at L1 or HLT. The lowest-threshold dielectron trigger (2e12_lhloose_L12EM10VH) applies a 12 $\text{GeV}$ transverse energy threshold and requires the two electrons to pass loose LH identification requirements. The trigger is seeded by L1_2EM10VH, which requires two electrons with $E_{\text{T}}$ above 10 $\text{GeV}$ and a hadronic energy veto.

The primary single-photon trigger used in 2015 is g120_loose. It requires a transverse energy above 120 $\text{GeV}$ and applies loose photon identification criteria. It is seeded by L1_EM22VHI, which requires an isolated electromagnetic cluster (denoted by I in the trigger name) with $E_{\text{T}}$ above 22 $\text{GeV}$ and applies a hadronic veto and $\eta$-dependent $E_{\text{T}}$ thresholds as described above. As mentioned earlier, the electromagnetic isolation and hadronic veto requirements are not applied for $E_{\text{T}}$ above 50 $\text{GeV}$. The two main diphoton triggers are g35_loose_g25_loose, which requires two photons above 35 and 25 $\text{GeV}$ thresholds and loose photon identification requirements, and 2g20_tight, which requires two photons with $E_{\text{T}}$ above 20 $\text{GeV}$ and tight identification. Both triggers are seeded by L1_2EM15VH, which requires two electromagnetic clusters with $E_{\text{T}}$ above 15 $\text{GeV}$ and a hadronic veto.

**Fig. 24:**
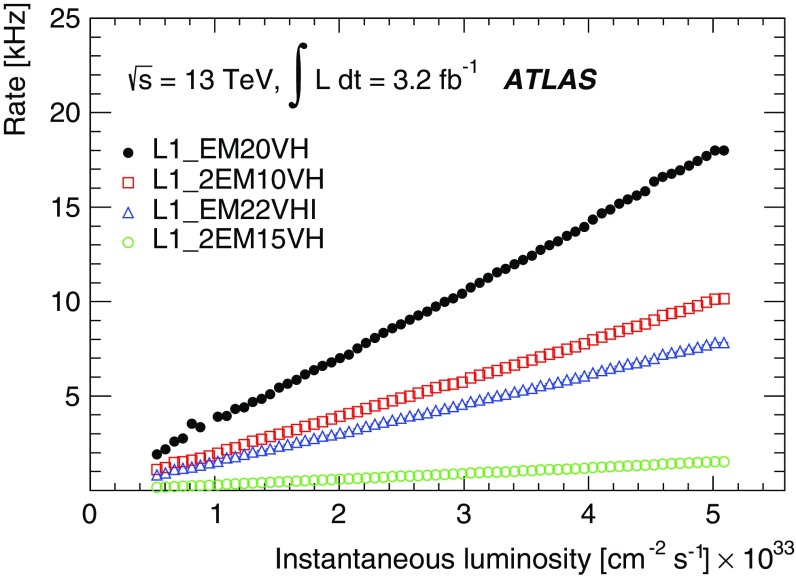
L1 trigger rates as a function of the instantaneous luminosity for selected single- and multi-object triggers

**Fig. 25:**
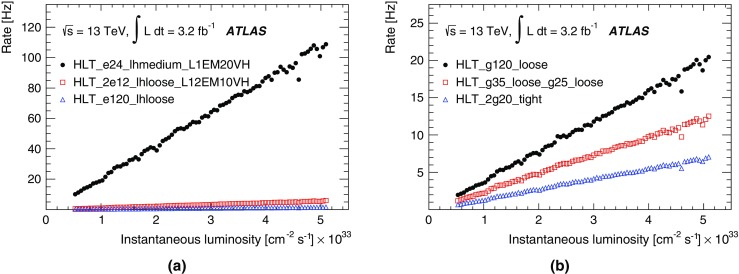
HLT trigger rates for **a** electron and **b** photon triggers as a function of the instantaneous luminosity for selected single- and multi-object triggers

Figures 24 and 25 show the rates of the electron and photon triggers as a function of the instantaneous luminosity. These trigger rates scale linearly with the instantaneous luminosity.

#### Electron and photon trigger efficiencies

**Fig. 26:**
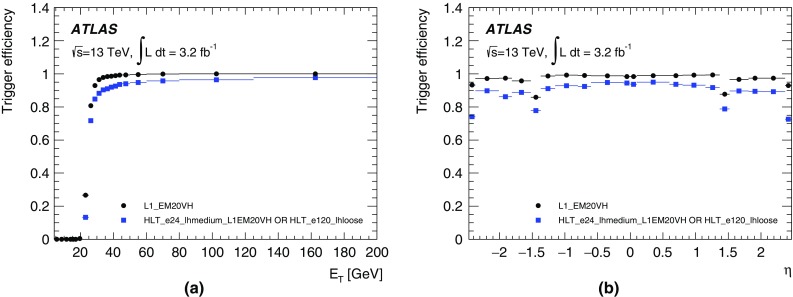
Efficiency of the L1_EM20VH trigger and the logical ‘or’ of the e24_lhmedium_L1EM20VH and e120_lhloose triggers as a function of **a** the probe electron transverse energy $E_{\text{T}}$ and **b** pseudorapidity $\eta$. The offline reconstructed electron candidate is required to have an $E_{\text{T}}$ value at least 1 $\text{GeV}$ above the trigger threshold

The performance of electron triggers is studied using a sample of $Z \rightarrow ee$ events. The tag-and-probe method utilises events triggered by a single-electron trigger and requires two offline reconstructed electrons with an invariant mass between 80 and 100 $\text{GeV}$. After identifying the electron that triggered the event (tag electron), the other electron (probe electron) is unbiased by the trigger selection, thus allowing its use to measure the electron trigger efficiency. HLT electrons (L1 EM objects) are matched to the probe electron if their separation is $\Delta R < 0.07 (0.15)$. The trigger efficiency is calculated as the ratio of the number of probe electrons passing the trigger selection to the number of probe electrons. The efficiency of the combination of the lowest unprescaled single-electron trigger e24_lhmedium_L1EM20VH and the high transverse momentum electron trigger e120_lhloose with respect to the offline objects is shown in Fig. 26 as a function of the offline reconstructed electron transverse energy and pseudorapidity. The figure also shows the efficiency of the L1 trigger (L1_EM20VH) seeding the lowest unprescaled single-electron trigger. A sharp turn-on can be observed for both the L1 and overall (L1 and HLT) efficiency, and the HLT inefficiency with respect to L1 is small. Inefficiencies observed around pseudorapidities of $-1.4$ and 1.4 are due to the transition region between the barrel and end-cap calorimeter.

**Fig. 27:**
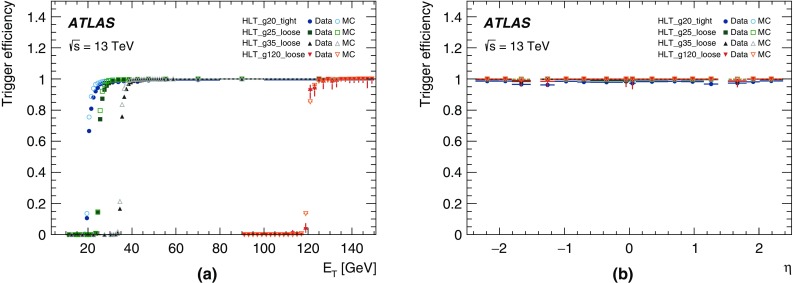
Efficiency of HLT photon triggers g20_tight, g25_loose, g35_loose, and g120_loose relative to a looser HLT photon trigger as a function of **a** the transverse energy $E_{\text{T}}$ and **b** pseudorapidity $\eta$ of the photon candidates reconstructed offline and satisfying the tight identification and isolation requirements. The offline reconstructed photon candidate is required to have an $E_{\text{T}}$ value at least 5 $\text{GeV}$ above the trigger threshold. The transition region between the barrel and end-cap calorimeter ($1.37<|\eta |<1.52$) is excluded

The photon trigger efficiency is computed using the bootstrap method as the efficiency of the HLT trigger relative to a trigger with a lower $E_{\text{T}}$ threshold. Figure 27 shows the efficiency of the main single-photon trigger and the photons of the main diphoton trigger as a function of the offline reconstructed photon transverse energy and pseudorapidity for data and MC simulation. Very good agreement is observed between data and simulation.

### Muons

Muons are produced in many final states of interest to the ATLAS physics programme, from SM precision physics to searches for new physics. Muons are identified with high purity compared to other signatures and cover a wide transverse momentum range, from a few $\text{GeV}$ to several $\text{TeV}$. Muon trigger thresholds in the $p_{\text{T}}$ range from 4 to 10 $\text{GeV}$ are used to collect data for measurements of processes such as $J/\psi \rightarrow \mu \mu$, low-$p_{\text{T}}$ dimuons, and $Z\rightarrow \tau \tau$ [36, 37]. Higher $p_{\text{T}}$ thresholds are used to collect data for new-physics searches as well as measuring the properties and production rates of SM particles such as the Higgs, $W$ and $Z$ bosons, and top quarks [38–40].

#### Muon reconstruction and selection

The trigger reconstruction algorithms for muons at L1 and the HLT are described in Sects. 3.2 and 5.3, respectively. The selection criteria depend on the algorithm used for reconstruction. The MS-only algorithm selects solely on the $p_{\text{T}}$ of the muon candidate measured by the muon spectrometer; the combined algorithm makes selections based on the match between the ID and MS tracks and their combined $p_{\text{T}}$; and the isolated muon algorithm applies selection criteria based on the amount of energy in the isolation cones.

#### Muon trigger menu and rates

The lowest-threshold single-muon trigger (mu20_iloose_L1MU15) requires a minimum transverse momentum of 20 $\text{GeV}$ for combined muon candidates in addition to a loose isolation: the scalar sum of the track $p_{\text{T}}$ values in a cone of size $\Delta R = 0.2$ around the muon candidate is required to be smaller than 12% of the muon transverse momentum. The isolation requirement reduces the rate by a factor of approximately 2.5 with a negligible efficiency loss. The trigger is seeded by L1_MU15, which requires a transverse momentum above 15 $\text{GeV}$. At a transverse momentum above 50 $\text{GeV}$ this trigger is complemented by a trigger not requiring isolation (mu50), to recover a small efficiency loss in the high transverse momentum region.

**Fig. 28:**
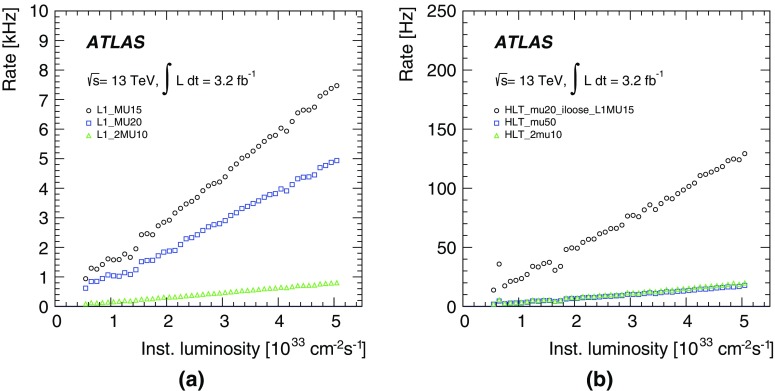
**a** L1 and **b** HLT muon trigger rates as a function of the instantaneous luminosity for primary single and dimuon triggers

The lowest-threshold unprescaled dimuon trigger (2mu10) requires a minimum transverse momentum of 10 $\text{GeV}$ for combined muon candidates. The trigger is seeded by L1_2MU10, which requires two muons with transverse momentum above 10 $\text{GeV}$. Figure 28 shows the rates of these triggers as a function of the instantaneous luminosity. The trigger rates scale linearly with the instantaneous luminosity. Dimuon triggers with lower $p_{\text{T}}$ thresholds and further selections (e.g. on the dimuon invariant mass) were also active and are discussed in Sect. 6.8. Additionally, an asymmetric dimuon trigger (mu18_mu8noL1) is included, where mu18 is seeded by L1_MU15 and mu8noL1 performs a search for a muon in the full detector at the HLT. By requiring only one muon at L1, the dimuon trigger does not suffer a loss of efficiency that would otherwise have if two muons were required at L1. This trigger is typically used by physics searches involving two relatively high-$p_{\text{T}}$ muons to improve the acceptance with respect to the standard dimuon triggers.

#### Muon trigger efficiencies

The L1 and HLT muon efficiencies are determined using a tag-and-probe method with $Z\rightarrow \mu \mu$ candidate events. Events are required to contain a pair of reference muons with opposite charge and an invariant mass within 10 $\text{GeV}$ of the $Z$ mass. Reference muons reconstructed offline using both ID and MS information are required to be inside the fiducial volume of the muon triggers ($|\eta |<2.4$) and pass the *medium* identification requirements [41, 42].

**Fig. 29:**
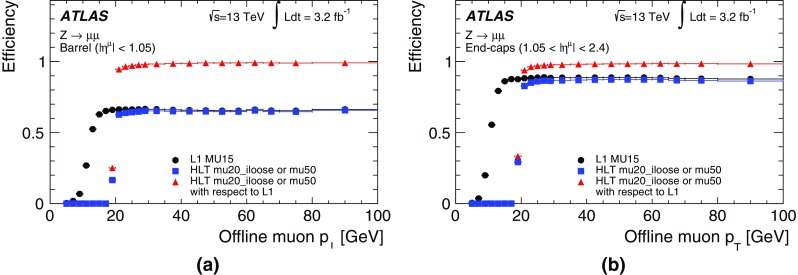
Efficiency of the L1 muon trigger L1_MU15 and the combination of the HLT muon triggers mu20_iloose_L1MU15 and mu50 as a function of the probe muon $p_{{\mathrm{T}}}$, separately for **a** the barrel and **b** the end-cap regions

**Fig. 30:**
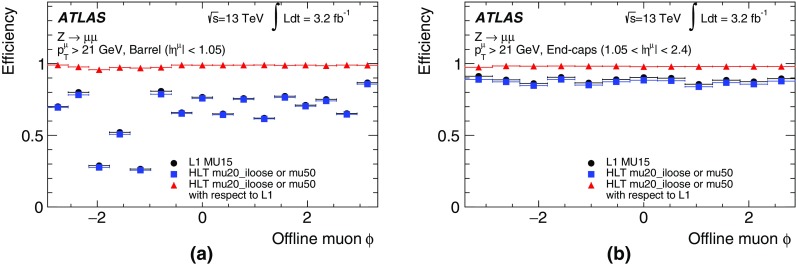
Efficiency of the L1 muon trigger L1_MU15 and the combination of the HLT muon triggers mu20_iloose_L1MU15 and mu50 as a function of the probe muon $\phi$, separately for **a** the barrel and **b** the end-cap regions

The absolute efficiency of the L1_MU15 trigger and the absolute and relative efficiencies of the logical ‘or’ of mu20_iloose and mu50 as a function of the $p_{\text{T}}$ of the offline muon track are shown in Fig. 29. The L1 muon trigger efficiency is close to 70% in the barrel and 90% in the end-caps. The different efficiencies are due to the different geometrical acceptance of the barrel and end-cap trigger systems and local detector inefficiencies. The HLT efficiency relative to L1 is close to 100% both in the barrel and in the end-caps. Figure 30 shows the muon trigger efficiency as a function of the azimuthal angle $\phi$ of the offline muon track for (a) the barrel and (b) the end-cap regions. The reduced barrel acceptance can be seen in the eight bins corresponding to the sectors containing the toroid coils and in the two feet sectors around $\phi \approx -1.6$ and $\phi \approx -2.0$, respectively.

### Jets

Jet triggers are used for signal selection in a wide variety of physics measurements and detector performance studies. Precision measurements of inclusive jet, dijet and multi-jet topologies rely on the events selected with the single-jet and multi-jet triggers. Events selected by the single-jet triggers are also used for the calibration of the calorimeter jet energy scale and resolution. All-hadronic decays of $t\bar{t}$ events can be studied using multi-jet signatures and the all-hadronic decay of the weak bosons, Higgs bosons and top quarks can be selected in high transverse momentum (‘boosted’) topologies using large-radius jets. Searches for physics beyond the SM, such as high-mass dijet resonances, supersymmetry or large extra dimensions, often utilise single-jet and multi-jet unprescaled triggers with a high transverse momentum threshold.

#### Jet reconstruction

A detailed description of the jet triggers used during Run 1 can be found in Ref. [5]. Jets are reconstructed in the HLT using the anti-$k_t$ jet algorithm [43] with a radius parameter of $R=0.4$ or $R=1.0$. The inputs to the algorithm are calorimeter topo-clusters that are reconstructed from the full set of calorimeter cell information calibrated by default at the EM scale. The jets are calibrated in a procedure similar to that adopted for offline physics analyses [44]. First, contributions to the jet energy from pile-up collisions are subtracted on an event-by-event basis using the calculated area of each jet and the measured energy density within $|\eta |<2$. Second, the response of the calorimeter is corrected using a series of $p_{\text{T}}$- and $\eta$-dependent calibration factors derived from simulation.

The jet reconstruction in the HLT is highly flexible and some triggers use non-standard inputs or a calibration procedure that differs from the default outlined above. For example, the clusters can be reconstructed using cells from a restricted region in the calorimeter defined using the RoIs identified by the L1 trigger. The clusters can also be calibrated using local calibration weights that are applied after classifying each cluster as electromagnetic or hadronic in origin. Furthermore, the jet calibration can be applied in four ways: no jet calibration, pile-up subtraction only, jet response correction only, or both pile-up subtraction and jet response corrections (default). Finally, the jet reconstruction can be run twice to produce *reclustered* jets [45], in which the input to the second jet-finding is the output from the first, e.g. to build large-*R* jets from small-*R* jets.

#### Jet trigger menu and rates

The jet trigger menu consists of *single-jet* triggers, which require at least one jet above a given transverse energy threshold, *multi-jet* triggers, which require at least *N* jets above a given transverse energy threshold, $H_{\text{T}}$ triggers, which require the scalar sum of the transverse energy of all jets in the event, $H_{\text{T}}$, above a given threshold, and *analysis-specific* triggers for specific topologies of interest. The jet triggers use at L1 either a random trigger (on colliding bunches) or an L1 jet algorithm. The random trigger is typically used for triggers that select events with offline jet $p_{\text{T}} <{45}\,{\text{GeV}}$ to avoid bias due to inefficiencies of the L1 jet algorithm for low-$p_{\text{T}}$ jets. In the following, only the most commonly used jet triggers are discussed.

The lowest-threshold unprescaled single-jet trigger for standard jets ($R=0.4$) selects events that contain a jet at L1 with transverse energy above 100 $\text{GeV}$ (L1_J100) and a jet in the HLT with transverse energy above 360 $\text{GeV}$ (j360). This trigger has a rate of 18 Hz at a luminosity of $5\times 10^{33}$ cm$^{-2}$ s$^{-1}$. The lowest-threshold unprescaled multi-jet triggers are 3j175, 4j85, 5j60 and 6j45, which have rates of 6, 20, 15 and 12 Hz, respectively. The lowest-threshold unprescaled $H_{\text{T}}$ trigger used in 2015 is ht850 with a rate of 12 Hz where one jet with transverse energy above 100 $\text{GeV}$ is required at L1 and $H_{\text{T}}$ is required to be above 850 $\text{GeV}$ at HLT.

In addition to the unprescaled triggers, a set of lower-threshold triggers select events that contain jets with lower transverse momentum and are typically prescaled to give an event rate of 1 Hz each. The lowest-threshold single-jet trigger in 2015 is j15, which uses a random trigger at L1. Multiple thresholds for single jets exist between j15 and j360 to cover the entire $p_{\text{T}}$ spectrum.

#### Jet trigger efficiencies

**Fig. 31:**
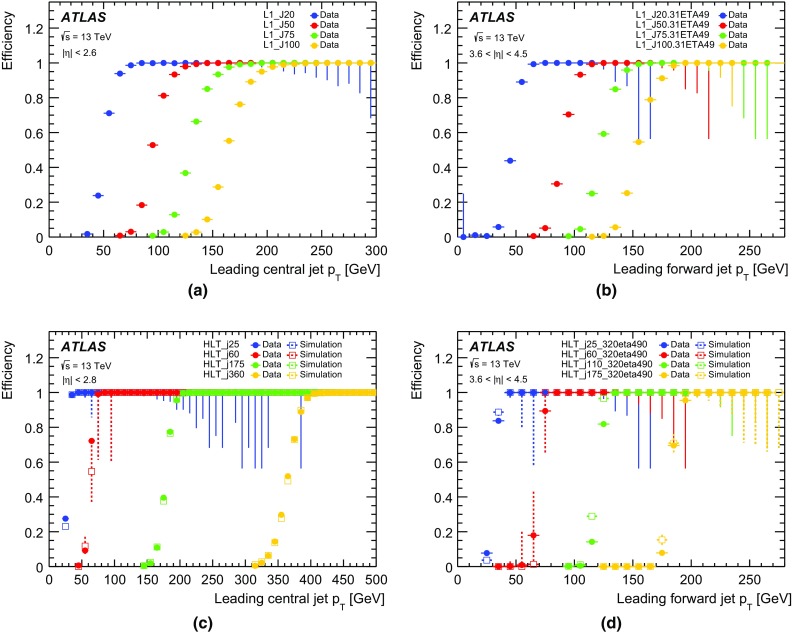
Efficiency of single-jet triggers as a function of offline jet $p_{\text{T}}$ for **a** L1 in the central region, **b** L1 in the forward region, **c** HLT in the central region, and **d** HLT in the forward region

**Fig. 32:**
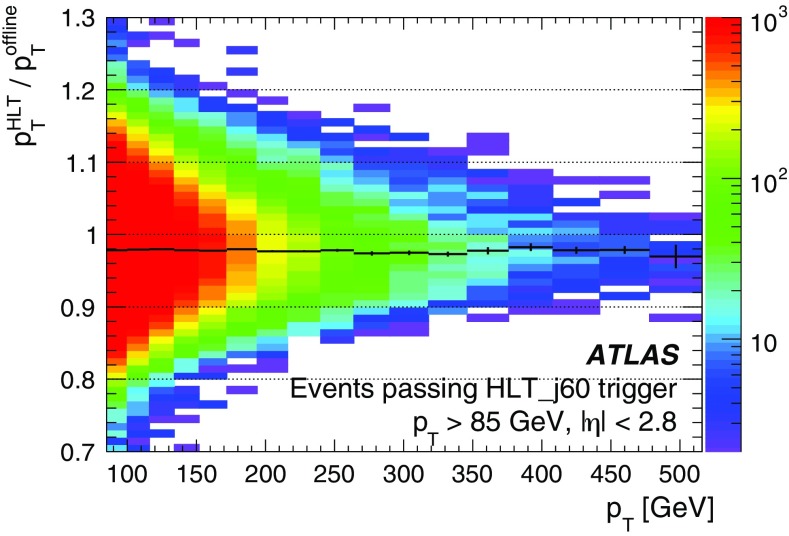
Comparison between the jet energy scales of trigger and offline jets. The *black points* represent the mean of the distribution at a given $p_{\text{T}}$ value. The 2% shift is due to differences in the jet calibration applied online and offline

Jet trigger efficiencies are determined using the bootstrap method with respect to the $p_{\text{T}}$ of the jet. The single-jet trigger efficiencies for L1 and the HLT are shown in Fig. 31 for both the central and forward regions of the calorimeter. The ranges in $|\eta |$ are chosen to ensure that the probe jet is fully contained within the $|\eta |$ region of study. Good agreement is observed between simulation and data. The sharp HLT efficiency turn-on curves in Fig. 31 are due to good agreement between the energy scale of jets in the HLT and offline, as shown in Fig. 32.

**Fig. 33:**
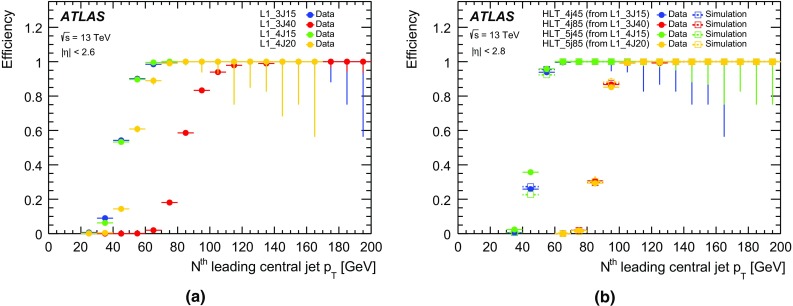
Efficiency of multi-jet **a** L1 and **b** HLT triggers as a function of offline jet $p_{\text{T}}$

The multi-jet trigger efficiencies are dominated by the trigger efficiency of the *N*th leading jet and are shown in Fig. 33 for (a) L1 and (b) HLT as a function of the *N*th leading jet transverse momentum. Good agreement is found for the efficiency as a function of the *N*th jet for different jet multiplicities with the same threshold (e.g. L1_6J15, L1_4J15 and 4j45, 5j45) and between data and simulation for the HLT.

**Fig. 34:**
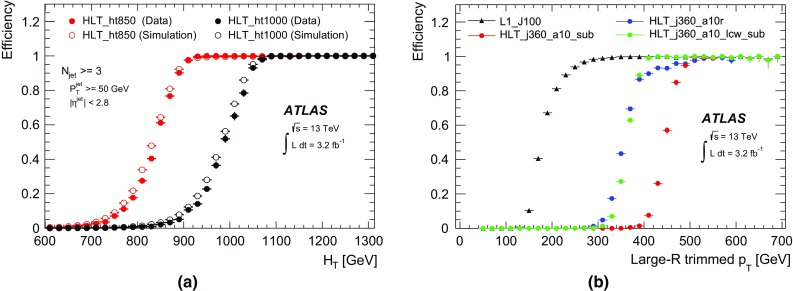
Efficiency of **a** $H_{\text{T}}$ triggers as a function of offline $H_{\text{T}}$ and **b** large-*R* ($R=1.0$) single-jet triggers as a function of offline $p_{\text{T}}$ . $H_{\text{T}}$ is defined as the summed transverse energy of all jets that are reconstructed above a transverse energy threshold of 50 $\text{GeV}$

Finally, the efficiency of the $H_{\text{T}}$ and large-*R* ($R=1.0$) triggers are shown in Fig. 34. The $H_{\text{T}}$ trigger efficiencies are measured with respect to the HLT_j150_L1J40 trigger. There is a small offset in the efficiency curves for data and simulation for both thresholds. For the large-*R* triggers, the HLT threshold is set to 360 $\text{GeV}$ and the efficiency curves are shown for three different calibrations and jet input options: jets built from topo-clusters at the EM scale with a pile-up subtraction applied (a10_sub), jets built from topo-clusters with local calibration weights and pile-up subtraction applied (a10_lcw_sub) and reclustered jets built from $R=0.4$ jets using both pile-up subtraction and local calibration weights (a10r).

#### Jets and trigger-level analysis

**Fig. 35:**
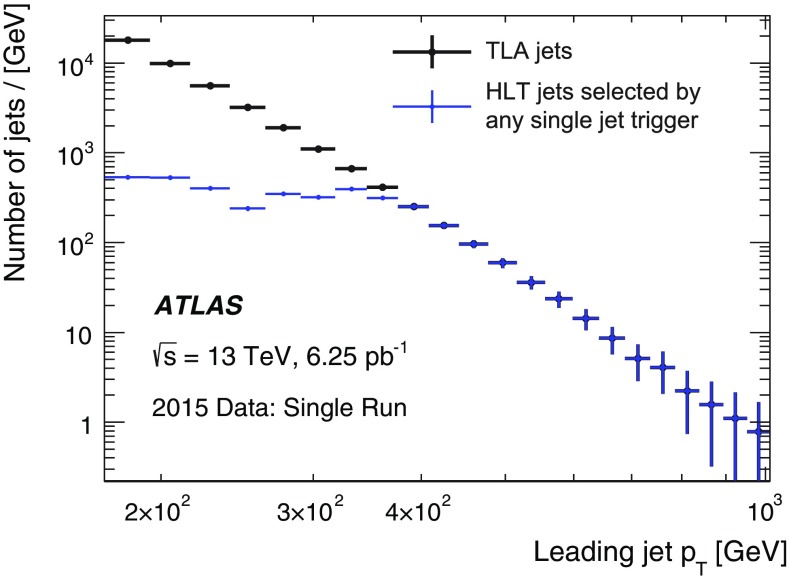
Jet $p_{\text{T}}$ spectrum after the basic kinematic selection for the TLA trigger jets (*black*) compared to trigger jets recorded by all single-jet triggers (*blue*)

Searches for dijet resonances with sub-$\text{TeV}$ masses are statistically limited by the bandwidth allocated to inclusive single-jet triggers. Due to large SM multi-jet backgrounds, these triggers must be prescaled in order to fit within the total physics trigger output rate of 1 kHz. However, as the properties of jets reconstructed at the HLT are comparable to that of jets reconstructed offline, one can avoid this rate limitation by using Trigger-Level Analysis (TLA) triggers that record partial events, containing only relevant HLT jet objects needed for the search, to a dedicated stream. Using Trigger-Level Analysis triggers allows a factor of 100 increase in the event recording rates, and results in a significant increase in the number of low-$p_{\text{T}}$ jets as shown in Fig. 35. Dedicated calibration and jet identification procedures are applied to these partially built events, accounting for differences between offline jets and trigger jets as well as for the lack of detector data other than from the calorimeters. These procedures are described in detail in Ref. [46].

### Tau leptons

Tau leptons are a key signature in many SM measurements and searches for new physics. The decay into tau lepton pairs provides the strongest signal for measurements of the SM Higgs boson coupling to fermions. Final states containing tau leptons are also often favoured by heavier Higgs bosons or other new resonances in many scenarios beyond the SM. Most (about 65%) of tau leptons decay hadronically. Hence an efficient trigger on hadronic tau decays is crucial for many analyses using tau leptons.

Dedicated tau trigger algorithms were designed and implemented based on the main features of hadronic tau decays: narrow calorimeter energy deposits and a small number of associated tracks. Due to the high production rate of jets with features very similar to hadronic tau decays, keeping the rate of tau triggers under control is particularly challenging.

#### Tau reconstruction and selection

At L1 the tau trigger uses the algorithms described in Sect. 3.1. The isolation requirement was tuned with 13 $\text{TeV}$ simulation to yield an efficiency of 98% and is not applied for tau candidates with a transverse energy above 60 $\text{GeV}$.

At the HLT three sequential selections are made. First, a minimum requirement is applied to the transverse energy of the tau candidate. The energy is calculated using the locally calibrated topo-clusters of calorimeter cells contained in a cone of size $\Delta R=0.2$ around the L1 tau RoI direction taken from the L1 cluster. A dedicated tau energy calibration scheme is used. Second, two-stage fast tracking (Sect. 5.1.3) is used to select tau candidates with low track multiplicity. A leading track is sought within a narrow cone ($\Delta R=0.1$) around the tau direction followed by a second fast tracking step using a larger cone ($\Delta R=0.4$) but with the tracks required to originate from within a fixed interval along the beam line around the leading track. Tracks with $p_{\text{T}} >{1}\,{\text{GeV}}$ are counted in the core cone region $\Delta R<0.2$ and in the isolation annulus $0.2<\Delta R<0.4$ around the tau candidate direction. A track multiplicity requirement selects tau candidates with $1\le N^{\mathrm{trk}}_{\Delta R<0.2}\le 3$ and $N^{\mathrm{trk}}_{0.2<\Delta R<0.4}\le 1$. Finally, the HLT precision tracking is run, and a collection of variables built from calorimeter and track variables are input to a Boosted Decision Tree (BDT), which produces a score used for the final tau identification. The implementation of those variables follows closely their offline counterparts as described in Ref. [47]. In addition, the same BDT training is used offline and online to ensure a maximal correlation between online and offline identification criteria. The performance of the offline training was found to be comparable to a dedicated online training. To ensure a robust response under differing pile-up conditions, corrections as a function of the average number of interactions per bunch-crossing are applied to the discriminating variables. Working points of the BDT are tuned separately for 1-prong and 3-prong candidates. The baseline *medium* working point operates with an efficiency of 95% (70%) for true 1-prong (3-prong) taus.

#### Tau trigger menu and rates

The primary tau triggers consist of triggers for single high transverse momentum taus, and combined $\tau +X$ triggers, where *X* stands for an electron, muon, a second tau or $E_{\text{T}}^{\text{miss}}$. The transverse momentum thresholds used in the single-tau and ditau triggers in 2015 are indicated in Table 1. For all tau triggers the L1 isolation, HLT track multiplicity and online *medium* identification requirements are applied to the tau candidates.

**Fig. 36:**
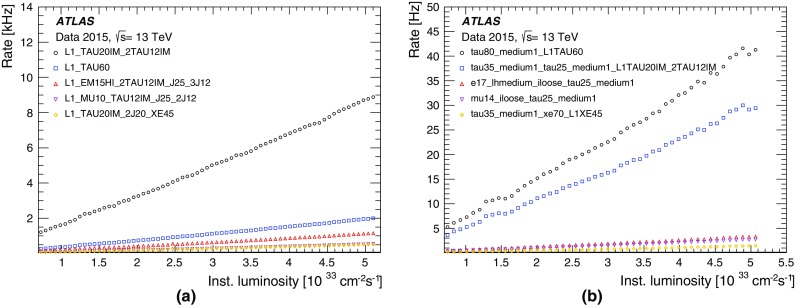
Trigger rates as a function of instantaneous luminosity for several **a** L1 and **b** HLT tau triggers

Due to L1 rate limitations, the combined triggers $\tau +(e,\mu )$ and $\tau +$ $E_{\text{T}}^{\text{miss}}$ require the presence of an additional jet candidate at L1 with transverse momentum above 25 and 20 $\text{GeV}$, respectively. Variants of these triggers with higher thresholds for the tau transverse momentum and without the L1 jet requirement are also included in the trigger menu. Figure 36 shows the L1 and HLT output rates as function of the instantaneous luminosity for the primary single-tau, ditau, $\tau +e$, $\tau +\mu$ and $\tau +$ $E_{\text{T}}^{\text{miss}}$ triggers.

#### Tau trigger efficiencies

The efficiency of the tau trigger was measured using a tag-and-probe (T&P) method in an enriched sample of $Z\rightarrow \tau _\mu \tau _{\mathrm{had}}\rightarrow \mu +2\nu +\tau _{\mathrm{had}}$ events, where $\tau _\mu$ is a tau lepton decaying to $\mu \nu \nu$ and $\tau _{\mathrm{had}}$ is a tau lepton decaying hadronically. Events are selected by the lowest unprescaled single-muon trigger and are tagged by an offline reconstructed and isolated muon with transverse momentum above 22 $\text{GeV}$. The presence of an offline reconstructed tau candidate with transverse momentum above 25 $\text{GeV}$, one or three tracks, fulfilling the *medium* identification criteria and with electric charge opposite to the muon charge is also required. This reconstructed tau candidate is the probe with respect to which the tau trigger efficiency is measured. The event selection used to enhance the sample with $Z\rightarrow \tau _\mu \tau _{\mathrm{had}}$ events and therefore the purity of the probe tau candidate is similar to the one described in Ref. [47]: to reject $Z(\rightarrow \mu \mu )+\text{jets}$ and $W(\rightarrow \mu \nu )+\text{jets}$ events, the invariant mass of the muon and the offline tau candidate is required to be between 45 and 80 $\text{GeV}$, the transverse mass, $m_{{\mathrm{T}}}$, composed of the muon $p_{\text{T}}$ and $E_{\text{T}}^{\text{miss}}$ ($m_{{\mathrm{T}}}^2=2p_{{\mathrm{T}}}^{\mu }E_{\text{T}}^{\text{miss}} (1-\cos \Delta \phi (\mu ,E_{\text{T}}^{\text{miss}}))$) is required to be smaller than 50 $\text{GeV}$, and the variable built from the difference in azimuth between the muon and $E_{\text{T}}^{\text{miss}}$ and between the offline tau candidate and $E_{\text{T}}^{\text{miss}}$ ($\cos \Delta \phi (\mu ,E_{\text{T}}^{\text{miss}})+\cos \Delta \phi (\tau ,E_{\text{T}}^{\text{miss}})$) is required to be above $-0.5$. The dominant sources of background events in the resulting sample are $W(\rightarrow \mu \nu )+\text{jets}$ and multi-jet events and their contributions are determined in data as described in Ref. [47]. The multi-jet contribution is estimated from events where the offline tau candidate and the muon have the same electric charge. The $W(\rightarrow \mu \nu )+\text{jets}$ contribution is estimated from events with high $m_{{\mathrm{T}}}$.

**Fig. 37:**
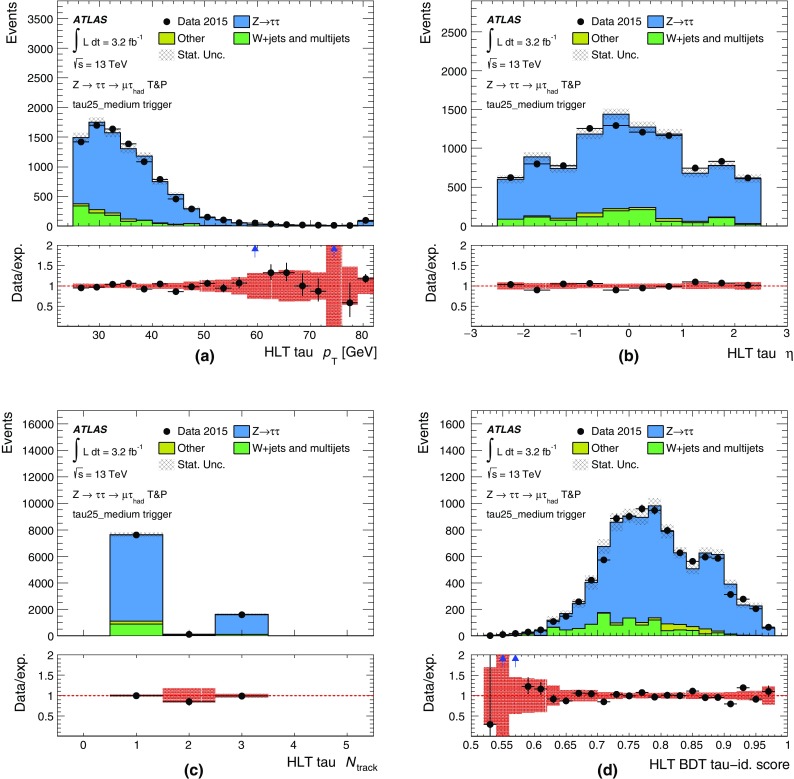
Distributions of the HLT tau candidates passing the tau25_medium trigger: **a** transverse momentum, **b** pseudorapidity, **c** track multiplicity distributions of the core tracks $\Delta R<0.2$ of the tau-axis and **d** online BDT identification score. The HLT tau candidates are matched to offline tau candidates with transverse momentum above 25 $\text{GeV}$, with one or three tracks and satisfying the offline *medium* tau identification criterion. Only statistical uncertainties are shown, and the last bin in **a** contains overflow events. The ratio of the observed data to the expected signal and background events is also shown, where the *red band* shows the statistical uncertainty of the total prediction

Distributions of the transverse momentum, pseudorapidity, track multiplicity and BDT discriminant score for the HLT tau candidates matched to the offline probe tau candidates are shown in Fig. 37. The HLT tau candidates pass the tau25_medium trigger, which requires an isolated L1 RoI with transverse momentum above 12 $\text{GeV}$ and a tau candidate at the HLT with transverse momentum above 25 $\text{GeV}$ satisfying the track multiplicity and the online *medium* identification criteria. The observed distributions in data are in good agreement with simulation.

**Fig. 38:**
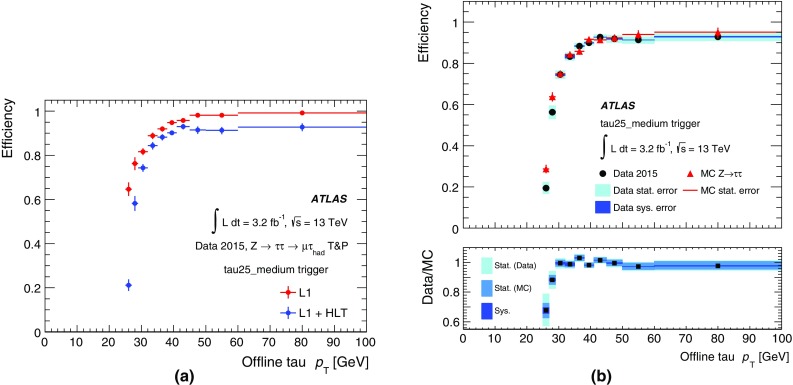
Efficiency of the tau25_medium trigger measured in data as a function of the offline tau $p_{\text{T}}$ for offline tau candidates with $p_{\text{T}}$ above 25 $\text{GeV}$, one or three tracks and satisfying the offline *medium* identification requirement. The expected background contribution has been subtracted from the data. **a** Efficiencies after the L1 (*red*) and L1+HLT (*blue*) selections are shown separately with only statistical uncertainties. **b** Comparison of the measured efficiency after L1+HLT to simulation. Statistical uncertainties associated with data and simulation and the systematic uncertainty associated with the background subtraction procedure in data are shown

The estimated background is subtracted from data and the uncertainty in this subtraction is considered as a systematic uncertainty in the measured efficiency. This systematic uncertainty includes uncertainties in the background contributions estimated from both simulation and data. Figure 38a shows the measured efficiency for the tau25_medium trigger as a function of the transverse momentum of the offline tau candidate. The efficiency loss of the HLT with respect to L1 is mainly due to the HLT’s track multiplicity selection and its BDT selection, which uses slightly different input variables online and offline. In Fig. 38b this efficiency is compared with simulation. The statistical uncertainties in data and simulation are shown together with the systematic uncertainties associated with the background subtraction procedure in data.

### Missing transverse momentum

The $E_{\text{T}}^{\text{miss}}$ trigger is used in searches where the final state contains only jets and large $E_{\text{T}}^{\text{miss}}$. The $E_{\text{T}}^{\text{miss}}$ trigger can also be the most efficient trigger for selecting final states that contain highly energetic muons. An example is searches for supersymmetric particle production where jets, leptons and invisible particles are produced. Another major use is for multi-particle final states where the combination of $E_{\text{T}}^{\text{miss}}$ with other trigger objects such as jets, electrons, or photons enables lower thresholds to be used for these other objects than would otherwise be possible. Finally, the $E_{\text{T}}^{\text{miss}}$ trigger collects data samples used for detector performance studies. For example, the data set used for electron efficiency calculations in events consistent with a *W* boson is selected with an $E_{\text{T}}^{\text{miss}}$ trigger.

#### $E_{\text{T}}^{\text{miss}}$ reconstruction and selection

The very large rate of hadronic jet production means that, even with reasonably good calorimeter resolution, jet energy mismeasurement can lead to an unaffordably large $E_{\text{T}}^{\text{miss}}$ trigger rate. The difficulty is exacerbated by pile-up collisions that add energy to the calorimeter and hence degrade the $E_{\text{T}}^{\text{miss}}$ resolution. Controlling the rate via increased trigger thresholds usually reduces the efficiency for analyses.

The improvements in the L1 $E_{\text{T}}^{\text{miss}}$ determination, including the L1 dynamic pedestal correction described in Sect. 3.1, have been important in maintaining L1 performance. In particular they have permitted the L1_XE50 trigger to be used without prescale throughout 2015.

To fulfil the desired broad $E_{\text{T}}^{\text{miss}}$-based physics programme, different HLT algorithmic strategies based on cells, jets or topo-clusters in addition to two methods for correcting the effects of pile-up were developed during LS1 and deployed during 2015 data-taking. While the offline algorithms do often include reconstructed muons in the $E_{\text{T}}^{\text{miss}}$ calculation, the trigger algorithms described herein use only energy measurements in the calorimeter. Five different algorithms, involving different levels of complexity (and thus different CPU requirements) were commissioned and evaluated with data during 2015. Since the time-consuming (topo-)clustering is shared between the different algorithms, running them all in parallel does only require a small amount of extra CPU time. The algorithms are as follows:

- **Cell algorithm** (xe): The measured energy in each LAr and Tile calorimeter cell, labelled *i*, and the position of the cell in the detector are used to obtain the components of the cell measured momentum in the massless approximation, i.e. $p_{x,i}=E_i \sin \theta _i\cos \phi _i$ and $p_{y,i}= E_i\sin \theta _i\sin \phi _i$. To suppress noise and cells with large negative energy, only those cells with energy satisfying $|E_i| > 2\sigma _i$ and $E_i > -5\sigma _i$, are considered further, where $\sigma _i$ is the noise in the cell energy measurement, including the noise-like effects from pile-up.^4^ Non-functioning calorimeter cells are masked out and do not contribute to the calculation. The total missing transverse momentum two-vector ${\vec {p}_{{\mathrm{T}}}^{\mathrm{\,miss}}}= -\sum _i (p_{x,i}, p_{y,i})$ is found from the remaining contributing cells, and the $E_{\text{T}}^{\text{miss}}$ calculated from its norm $E_{\text{T}}^{\text{miss}} =|{\vec {p}_{{\mathrm{T}}}^{\mathrm{\,miss}}}|$.
- **Jet-based algorithm** (xe_tc_mht): $E_{\text{T}}^{\text{miss}}$ is calculated directly from the negative of the transverse momentum vector sum of all jets reconstructed by the jet trigger algorithm presented in Sect. 6.4, which have been corrected for the energy contribution from pile-up.
- **Topo-cluster algorithm** (xe_tc_lcw): Topo-clusters (described in Sect. 5.2.1) are built for the entire calorimeter and used for the $E_{\text{T}}^{\text{miss}}$ reconstruction. For each topo-cluster *j*, the momentum components $(p_{x,j}, p_{y,j})$ are calculated in the approximation that the particles contributing energy to the cluster are massless, and, in a manner similar to the cell algorithm, the missing transverse momentum is calculated from the negative vector sum of these components.
- **Pile-up suppression algorithm** (xe_tc_pueta): This algorithm is based on the topo-cluster $E_{\text{T}}^{\text{miss}}$ algorithm described above, but includes a further pile-up suppression method that is intended to limit the degradation of the $E_{\text{T}}^{\text{miss}}$ resolution at very high pile-up. The method starts by calculating the average topo-cluster energy and standard deviation in ten regions of pseudorapidity covering, in equal steps, $-5.0<\eta <5.0$ in the calorimeter. In each pseudorapidity region, known as a ring, the topo-clusters of energy above $2\sigma$ are omitted and the average energy of the residual topo-clusters is calculated. This average represents an estimate of the energy contribution from pile-up in that ring. The pile-up energy density in each ring is obtained by dividing the average energy by the solid angle of the ring. This energy density is then multiplied by the solid angle of each topo-cluster and then subtracted from the energy of that topo-cluster to obtain a topo-cluster energy measurement corrected for pile-up. The $E_{\text{T}}^{\text{miss}}$ is recalculated as described above using the $(p_{x,j}, p_{y,j})$ of topo-clusters after the pile-up subtraction.
- **Pile-up fit algorithm** (xe_tc_pufit): Starting again from the topo-cluster $E_{\text{T}}^{\text{miss}}$ described above, a different pile-up suppression method is used in this algorithm. The calorimeter is partitioned into 112 towers each of size $\eta \times \phi \approx 0.71\times 0.79$. For each tower, the $p_x$ and $p_y$ components of all the topo-clusters with centres in that tower are summed to obtain the transverse momentum ${\vec {p}}_{{{\mathrm{T}}},k}$ of that *k*th tower. The transverse energy sum of the tower $E_{{\mathrm{T}},k}$ is also calculated from the scalar sum of the $p_{\text{T}}$ of the individual clusters. If $E_{{{\mathrm{T}}},k} < {45}\,{\text{GeV}}$, the tower is determined to be below threshold and its energy assumed to be due to pile-up. The average pile-up $E_{\text{T}}$ density is calculated from $\sum _k E_{{\mathrm{T}},k}/\sum _k A_k$ of all the towers below threshold, where $A_k$ is the total area in $(\eta ,\phi )$ coordinates of those towers. A fit estimates the $E_{\text{T}}$ contributed by pile-up in each tower above threshold using the average pile-up $E_{\text{T}}$ density and constraining the event-wide $E_{\text{T}}^{\text{miss}}$ from pile-up to be zero within resolution. These estimated pile-up contributions are subtracted from the corresponding $E_{\text{T}}$ measurements for towers above threshold, and these corrected $E_{\text{T}}$ values are used to calculate $E_{\text{T}}^{\text{miss}}$.

**Fig. 39:**
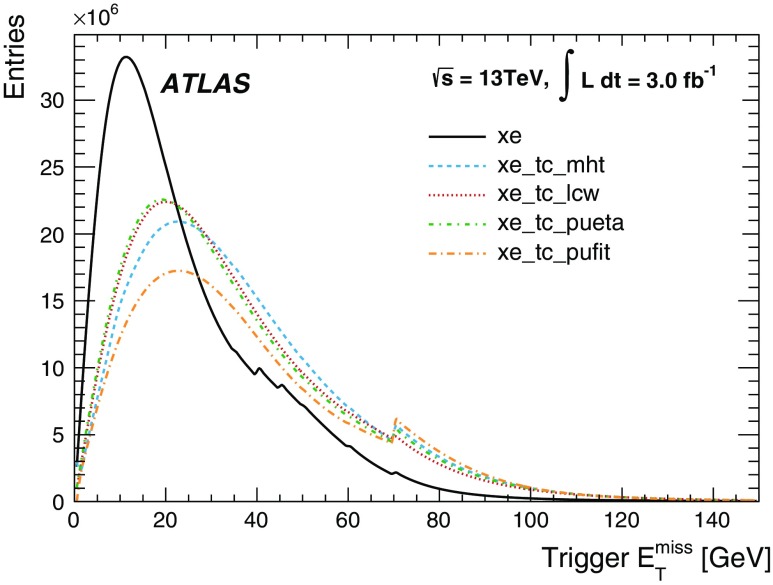
Comparison of the different $E_{\text{T}}^{\text{miss}}$ distributions for events accepted by the HLT into the *Main* physics stream. The algorithms consist of a cell-based $E_{\text{T}}^{\text{miss}}$ (xe) and different topo-cluster-based algorithms described in the text. The zero entries of the xe_tc_pufit algorithm, which occur when no tower is above threshold, have been suppressed. The steps in the distributions are caused by the various trigger thresholds

Figure 39 shows the $E_{\text{T}}^{\text{miss}}$ distribution of the various HLT algorithms for events accepted into the *Main* physics stream. The differences observed between the cell-based and the topo-cluster-based $E_{\text{T}}^{\text{miss}}$ distributions are caused in part by different calibration; the cell-based algorithm is calibrated at the EM scale, while algorithms based on topo-clusters generally have larger values of $E_{\text{T}}^{\text{miss}}$ as they include a correction for the calorimeter response to hadrons (hadronic scale). Differences between the $E_{\text{T}}^{\text{miss}}$ distributions for the various pile-up correction schemes are small, since these algorithms were optimised to improve the resolution at large pile-up values of 80 overlapping interactions that will only be achieved in future LHC runs.

#### $E_{\text{T}}^{\text{miss}}$ trigger menu and rates

**Fig. 40:**
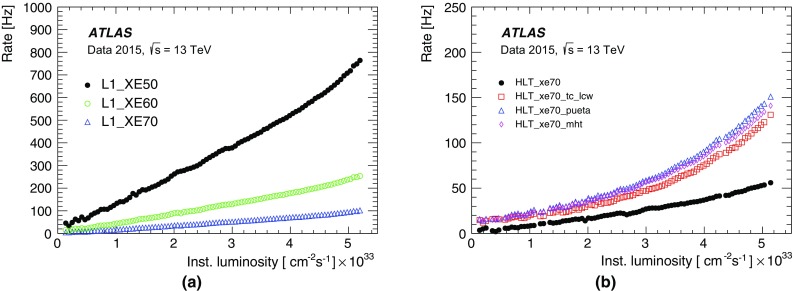
$E_{\text{T}}^{\text{miss}}$ trigger rates (**a**) at L1 and (**b**) for various HLT algorithms operating with nominal thresholds of 70 $\text{GeV}$. The HLT algorithms are each seeded by L1_XE50. Rates are shown as a function of instantaneous luminosity from various runs taken in 2015 excluding periods with atypically high or low rates arising from different pile-up conditions for the same instantaneous luminosity

All the primary HLT $E_{\text{T}}^{\text{miss}}$ algorithms used in 2015 were seeded by the L1_XE50 trigger with a nominal threshold, calibrated at the EM scale, of 50 $\text{GeV}$. The L1_XE50 output rate was approximately 700 Hz at an instantaneous luminosity of $5\times 10^{33}$ cm$^{-2}$ s$^{-1}$ as shown in Fig. 40a. The HLT xe trigger with a threshold of 70 $\text{GeV}$ remained unprescaled throughout the 2015 data-taking period. The typical output rate for this trigger was approximately 50 Hz at the same luminosity as seen in Fig. 40b. The topo-cluster-based algorithms, all of which are calibrated at the hadronic scale, had rates of approximately 110 Hz at the equivalent nominal threshold of 70 $\text{GeV}$. The output rate from these algorithms is larger for the same nominal threshold due in part to the different calibration methods. Prescaled triggers at a set of lower L1 and HLT thresholds, with HLT output rates of order 1 Hz each, were included in the menu to record a sample of data from which the efficiency of the unprescaled, primary physics triggers could be calculated. Further triggers based on the significance of the observed $E_{\text{T}}^{\text{miss}}$ , known as xs triggers [48] were used to select $W\rightarrow e\nu$ events for electron reconstruction performance studies. Triggers used during Run 1 for selecting events based on the scalar sum of the transverse energy of all calorimeter cells $\Sigma E_{\text{T}}$ were found to have a high sensitivity to pile-up [48], and so were not used during the proton–proton run in 2016.^5^

#### $E_{\text{T}}^{\text{miss}}$ trigger efficiencies

**Fig. 41:**
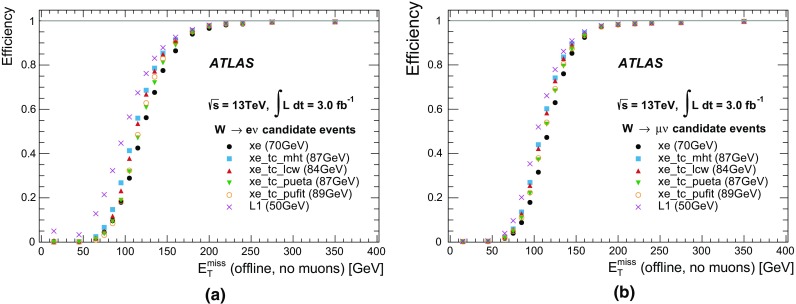
$E_{\text{T}}^{\text{miss}}$ trigger efficiency curves with respect to the $E_{\text{T}}^{\text{miss}}$ reconstructed offline without muon corrections for all events passing the **a** $W\rightarrow e\nu$ or **b** $W\rightarrow \mu \nu$ selections. The different efficiencies were measured for L1, and for the combination of L1 with each of the HLT $E_{\text{T}}^{\text{miss}}$ algorithms. The thresholds for the different algorithms correspond to an approximately equal trigger rate

Since $E_{\text{T}}^{\text{miss}}$ is a global observable calculated from many contributions, each of which has its own detector resolution, the efficiency of the $E_{\text{T}}^{\text{miss}}$ trigger for any particular analysis inevitably depends on the event selection used in that analysis. The efficiency turn-on curves of the various $E_{\text{T}}^{\text{miss}}$ trigger algorithms are shown in Fig. 41, for $W\rightarrow e\nu$ and $W\rightarrow \mu \nu$ selections. The selection is similar to that of the *W* boson cross-section measurement [39], requiring exactly one lepton (electron or muon) with $p_{\text{T}} >{25}\,{\text{GeV}}$, transverse mass $m_{{\mathrm{T}}}>{50}\,{\text{GeV}}$, and a single lepton trigger (24 $\text{GeV}$ single-electron or 20 $\text{GeV}$ single-muon). The efficiencies are shown as a function of a modified offline $E_{\text{T}}^{\text{miss}}$ calculation with no muon correction, emulating the calorimeter-only $E_{\text{T}}^{\text{miss}}$ calculation used in the trigger. The event kinematics for the same $E_{\text{T}}^{\text{miss}}$ are very different for the decays into electron and muon, since the energy of the electron for $W\rightarrow e\nu$ is included in both the online and offline calculations of $E_{\text{T}}^{\text{miss}}$, whereas this is not the case for the muon in $W\rightarrow \mu \nu$. Events with high $p_{\text{T}}$ muons are recorded by the muon triggers.

The turn-on curves are shown for different nominal HLT $E_{\text{T}}^{\text{miss}}$ thresholds, selected such that they give rates close to that of the xe algorithm at its lowest unprescaled (70 $\text{GeV}$) threshold. All the HLT algorithms, with their stated thresholds, are close to fully efficient with respect to the offline $E_{\text{T}}^{\text{miss}}$ for values of $E_{\text{T}}^{\text{miss}} >{200}\,{\text{GeV}}$. At that value of $E_{\text{T}}^{\text{miss}}$, the L1_XE50 trigger itself has an efficiency in the range of 95–99%, depending on the exact event selection required. The topo-cluster-based algorithms, and in particular xe_tc_mht have higher efficiency in the turn-on region than the cell-based algorithm.

**Fig. 42:**
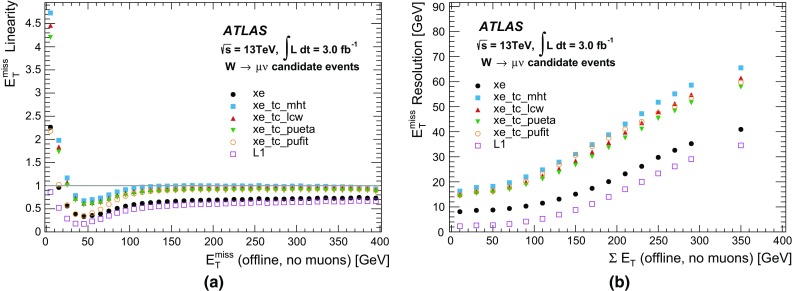
**a** $E_{\text{T}}^{\text{miss}}$ trigger linearity with respect to the $E_{\text{T}}^{\text{miss}}$ reconstructed offline without muon corrections and **b** $E_{\text{T}}^{\text{miss}}$ trigger resolution with respect to the $\Sigma E_{\text{T}}$ reconstructed offline without muon corrections, for all events passing $W\rightarrow \mu \nu$ selections for L1 and for each HLT $E_{\text{T}}^{\text{miss}}$ algorithm. Linearity and resolution are defined in the text

The linearity of the $E_{\text{T}}^{\text{miss}}$ trigger is defined as the average ratio of the trigger $E_{\text{T}}^{\text{miss}}$ to the offline $E_{\text{T}}^{\text{miss}}$ . The linearity of the L1 algorithm and the various HLT algorithms is shown in Fig. 42a. For the larger values of offline $E_{\text{T}}^{\text{miss}}$ where the triggers approach full efficiency, the topo-cluster-based HLT algorithms show good linearity at values close to unity. The L1 and the xe HLT algorithms also show stable linearity in the trigger efficiency plateau, but at a lower value, reflecting their calibration at the EM scale rather than the hadronic scale.

The $E_{\text{T}}^{\text{miss}}$ resolution is defined as the RMS of the *x*-component of the core of the ${\vec {p}_{{\mathrm{T}}}^{\mathrm{\,miss}}}$ distribution. Since the resolution is dominated by the stochastic fluctuations in calorimeter energy measurements, it is shown in Fig. 42b as a function of the offline value of $\Sigma E_{\text{T}}$ (reconstructed offline without muon corrections). The expected approximate scaling of $E_{\text{T}}^{\text{miss}}$ with $\sqrt{\Sigma E_{\text{T}}}$ can be observed. The stochastic contribution to the resolution can be seen to be accompanied by an offset that varies from algorithm to algorithm and that is lower in the cell-based, electromagnetically calibrated L1 and xe algorithms. Such differences are expected because different noise suppression schemes are used to define calorimeter cells and topological clusters.

**Fig. 43:**
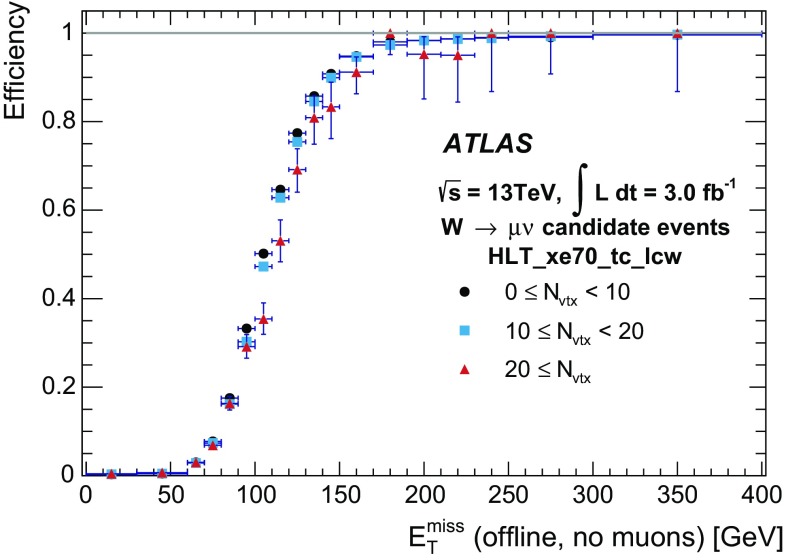
$E_{\text{T}}^{\text{miss}}$ trigger efficiency curves with respect to the $E_{\text{T}}^{\text{miss}}$ reconstructed offline without muon corrections for the $W\rightarrow \mu \nu$ selection. The different efficiencies were obtained for different pile-up conditions expressed in terms of various ranges of the average number of reconstructed vertices per bunch-crossing (denoted here as $N_{\mathrm{vtx}}$). The efficiency of the L1 algorithm is included

Figure 43 shows the efficiency of the trigger-level $E_{\text{T}}^{\text{miss}}$ algorithm for $W\rightarrow \mu \nu$ events for several ranges of the number of reconstructed vertices. The effect of pile-up on the $E_{\text{T}}^{\text{miss}}$ turn-on curves can be seen in this figure for the topo-cluster algorithm (xe_tc_lcw), which does not employ any pile-up correction methods. Some degradation of efficiency is observed for larger numbers of proton–proton vertices $N_{\mathrm{vtx}}$. The larger pile-up both increases the trigger rate, through increasing the probability to pass the trigger at lower $E_{\text{T}}^{\text{miss}}$ , and degrades the efficiency in the turn-on region.

### *b*-Jets

Bottom-quark-initiated jet (‘*b*-jet’) triggers are designed to identify heavy-flavour content in real time and provide the means to efficiently record events with fully hadronic final states containing *b*-jets. Various signatures from the Higgs boson or physics beyond the SM rely on triggering on *b*-jets. These include the SM processes $t\bar{t} H(H\rightarrow b\bar{b})$ and vector-boson fusion production with $H\rightarrow b\bar{b}$, the supersymmetric decay $bA\rightarrow bb\bar{b}$, search for di-*b*-jet resonances, and resonant and non-resonant Higgs boson pair production $HH\rightarrow b\bar{b} b\bar{b}$.

#### *b*-Jet reconstruction and selection

Several *b*-hadron properties are exploited to identify (tag) *b*-jets. The *b*-hadrons have a mean lifetime of $\sim$1.5 ps and often travel several millimetres before decaying. Consequently, a secondary vertex (SV) displaced from a primary interaction point characterises the decay. Reconstructed tracks associated with this SV have large transverse and longitudinal ($z_0$) impact parameters with respect to the primary vertex. In addition, *b*-hadrons go through hard fragmentation and have a relatively high mass of about 5 $\text{GeV}$. Thus, in addition to the decay length, b-jets can be distinguished from light-quark jets by having a large invariant mass, a large fraction of jet energy carried by tracks and a large track multiplicity.

As track and vertex reconstruction are crucial for the identification of *b*-jets, the *b*-jet trigger relies heavily on the performance of the ID tracking described in Sect. 5.1. Several improvements in the ID tracking made for Run 2 have directly benefited the *b*-jet trigger. The new IBL improves the impact parameter resolution of reconstructed tracks, leading to better *b*-jet identification and overall performance of the *b*-jet triggers [7]. Another improvement for Run 2 is the multiple-stage tracking described in Sect. 5.1.3. This new approach provides improved primary vertex finding and mitigates CPU requirements in the face of increased pile-up.

**Fig. 44:**
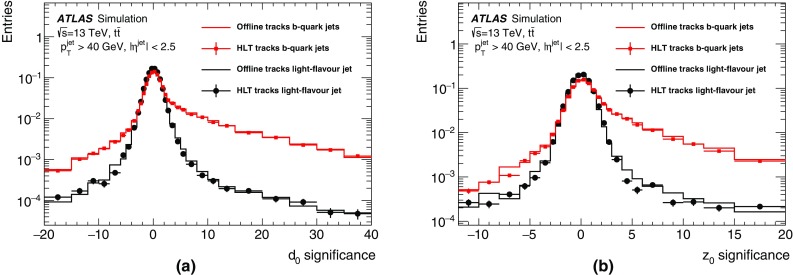
**a** Transverse and **b** longitudinal impact parameter significance for tracks associated with light-flavour (*black*) and *b*-quark (*red*) jets measured in a sample of simulated $t\bar{t}$ events. The *solid lines* show the distribution for the offline tracks. The *points* show the corresponding distribution for tracks used in the *b*-jet trigger. The impact parameter significance is defined as the impact parameter divided by the associated uncertainty. The impact parameters are signed such that track displacements in the direction of the jet have positive values, while tracks with displacements opposite of the jet direction are negative

The basic inputs to *b*-tagging are reconstructed jets, reconstructed tracks and the position of the primary vertex. The jet reconstruction used in the trigger is described in Sect. 6.4.1. The *b*-jet trigger uses tracks from the precision stage of the ID trigger reconstruction. The beam-spot location is used for the position of the primary vertex in the plane transverse to the beam line. Dedicated algorithms are run online to reconstruct and monitor the position of the beam spot in real time. The position of the primary vertex along the beam line is taken from the *z* position of the primary vertex reconstructed as described in Sect. 5.1.3. Distributions of the transverse and longitudinal impact parameter significances for light-flavour and *b*-quark jets are shown in Fig. 44 for a sample of simulated $t\bar{t}$ events. Tracks used in the online *b*-tagging are compared to the corresponding tracks used offline.

During Run 1, the *b*-jet triggers used a combination of two likelihood-based algorithms, IP3D and SV1 [49]. The IP3D algorithm discriminates between *b*- and light-jets using the two-dimensional distribution of the longitudinal and transverse impact parameter significances. The SV1 algorithm exploits properties of the secondary vertex such as the invariant mass of tracks matched to the vertex, the fraction of the jet energy associated with the secondary vertex and the number of two-track vertices. These Run 1 algorithms, optimised for Run 2 conditions, were used during 2015 data-taking. Three operating points, *loose*, *medium* and *tight*, are defined to correspond to *b*-jet identification efficiencies obtained from simulated $t\bar{t}$ events of 79, 72 and 62%, respectively.

**Fig. 45:**
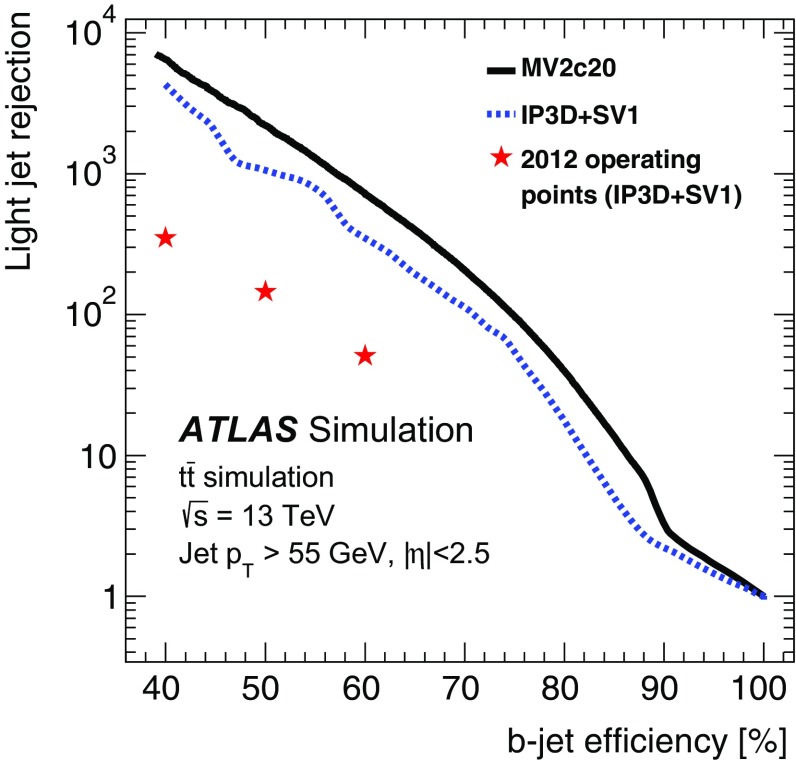
The expected performance of the MV2c20 trigger tagger (*solid black line*) in terms of light-jet rejection is shown together with the expected performance of the IP3D+SV1 trigger tagger in Run 2 (*dashed blue line*) and its actual performance achieved during Run 1 (*red stars*)

Another major development in the *b*-jet trigger for Run 2 is the adaptation of the offline *b*-tagging algorithms [50] for use in the trigger. The use of the offline MV2 multivariate *b*-tagging algorithm provides better online *b*-jet identification and leads to a higher level of coherence between the online and offline *b*-tagging decisions. The MV2 algorithm uses inputs from the IP3D, SV1 and JetFitter algorithms. The JetFitter algorithm exploits the topological structure of weak *b*- and *c*-hadron decays inside the jet. The MV2 algorithm used in the trigger was optimised to identify *b*-jets using a training sample with a background composition of 80% (20%) light- (*c*-) jets and is referred to as MV2c20. Operating points analogous to *loose*, *medium* and *tight* were defined for MV2c20 and give light-flavour rejections similar to the corresponding operating points of the Run 1 *b*-tagging algorithm. Triggers utilising the MV2c20 *b*-tagging algorithm were run in 2015 for commissioning purposes. MV2c20 is the baseline *b*-tagging algorithm for 2016. Figure 45 shows the expected performance of the MV2c20 and the IP3D+SV1 trigger taggers in Run 2 compared to the actual performance of the IP3D+SV1 tagger that was achieved during Run 1.

**Fig. 46:**
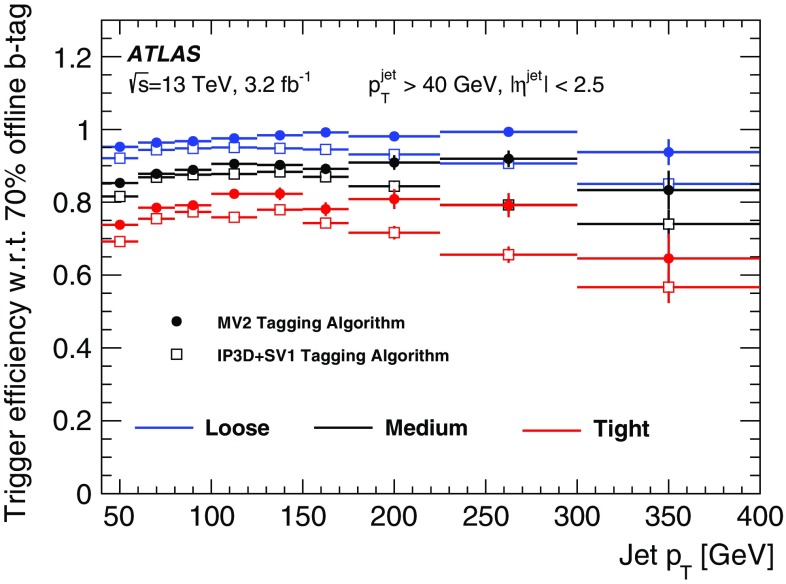
*b*-jet trigger efficiency as a function of jet $p_{\text{T}}$ for the loose (*blue*), medium (*black*) and tight (*red*) operating points. The *open squares* show the trigger efficiency using the IP3D+SV1 *b*-tagging algorithm. The *closed circles* show the trigger efficiency for the corresponding MV2 *b*-tagging algorithm working points. The efficiencies are measured in a pure sample of *b*-jets selected in $t\bar{t}$ events and are computed with respect to jets identified by the 70% working point of the offline MV2c20 *b*-tagging algorithm

Figure 46 shows the efficiency of the online *b*-tagging as a function of jet $p_{\text{T}}$ for the three operating points. The efficiencies are calculated in a pure sample of *b*-jets from fully leptonic $t\bar{t}$ decays and are computed with respect to jets identified by the 70% working point of the MV2c20 algorithm. Events used in the efficiency calculation require an online jet with $p_{\text{T}}$ greater than 40 $\text{GeV}$. A significant gain in trigger efficiency is seen when moving to the MV2 *b*-tagging algorithms.

#### *b*-Jet trigger menu and rates

**Fig. 47:**
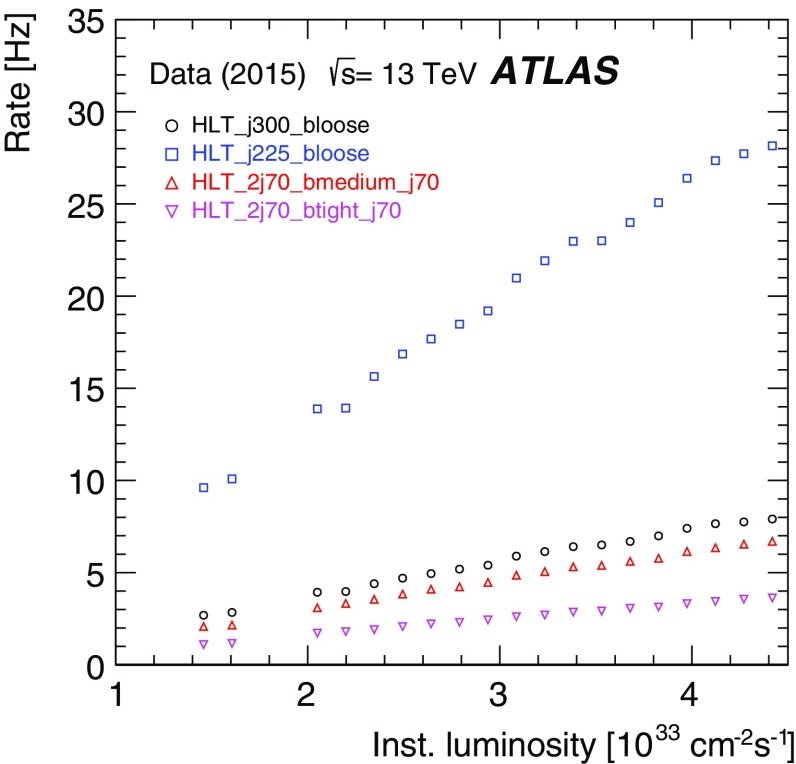
Rates of *b*-jet triggers as a function of the instantaneous luminosity

Several *b*-jet triggers have been implemented with different combinations of jets and *b*-tagged jets, using different $p_{\text{T}}$ thresholds and *b*-tagging operating points. The operating points, thresholds and multiplicities, for several of the primary *b*-jet triggers are listed in Table 1. The jet multiplicities vary between one and four, with up to two *b*-tagged jets. The *b*-jet triggers are typically seeded at L1 using either a single jet with $E_{\text{T}} >{100}\,{\text{GeV}}$ or three jets with $E_{\text{T}} >{25}\,{\text{GeV}}$ and pseudorapidity $|\eta | < 2.5$. Rates of various *b*-jet triggers as a function of luminosity are shown in Fig. 47.

The benefit of exploiting *b*-tagging in the HLT can be seen by comparing the thresholds used in jet triggers with and without *b*-tagging. The threshold for the lowest unprescaled single-jet trigger without *b*-tagging is 360 $\text{GeV}$. A *loose* requirement in the trigger allows this threshold to be lowered to 225 $\text{GeV}$. For the four-jet trigger, 85 $\text{GeV}$ thresholds are used when no *b*-tagging is applied. Requiring two jets to satisfy the *tight* *b*-tagging requirement allows the four-jet threshold to be lowered to 35 $\text{GeV}$.

### *B*-physics

The trigger selection of events for *B*-physics analyses is primarily based on the identification of *b*-hadrons through decays including a muon pair in the final state. Examples are decays with charmonium, $B\rightarrow J/\psi (\rightarrow \mu \mu )X$, rare decays $B^0_{(s)}\rightarrow \mu \mu$, and semileptonic $B\rightarrow \mu \mu X$. Decays of prompt charmonium and bottomonium are also identified through their dimuon decays, and are therefore similar to *b*-hadron decays, apart from the lack of measurable displacement from the *pp* interaction point.

#### *B*-physics reconstruction and selection

The primary suite of triggers require two muons at L1. Their rate is substantially reduced compared to single-muon L1 triggers. However, this results in inefficiencies at high transverse momentum, where the opening angle of the two muons becomes small for low-mass resonances, and the granularity at L1 is not sufficient to form separate RoIs. At the HLT, muons are reconstructed using the same algorithms as described in Sect. 5.3 with the additional requirement that the two muons should have opposite charges and form a good vertex (where the fit is performed using the ID track parameters) within a certain invariant mass window. The primary triggers use three dimuon mass windows: 2.5 to 4.3 $\text{GeV}$ intended for the selection of $J/\psi$ and $\psi (2\mathrm{S})$ decays into muon pairs (including charmonia produced in *b*-hadron decays), 4.0 to 8.5 $\text{GeV}$ for $B^0_{(s)}\rightarrow \mu \mu$ decays, and 8 to 12 $\text{GeV}$ for $\Upsilon (1,2,3\mathrm{S})\rightarrow \mu \mu$ decays. These invariant mass selections are indicated by the bJpsimumu, bBmumu and bUpsimumu suffixes in the trigger names, respectively.

Additional primary and supporting triggers are also implemented. Triggers using a single L1 muon RoI with an additional track found at the HLT do not have similar opening angle issues, but suffer from high rates and run with high prescale factors. These combined muon triggers are, however, essential components in data-driven estimates of the dimuon trigger efficiencies. Triggers requiring three muons at L1 help to maintain the lowest muon $p_{\text{T}}$ thresholds for certain event signatures with a likely presence of a third muon. Finally, for selecting semileptonic decays, such as $B^0\rightarrow \mu \mu K^{*0}(\rightarrow K^+\pi^-)$, searches for additional ID tracks and a combined vertex fit are performed assuming a few exclusive decay hypotheses. This reduces the rate with respect to a simple dimuon vertex selection thus allowing the dimuon mass window to be widened to the full kinematically allowed range. The corresponding trigger names use the bBmumuxv2 suffix.

#### *B*-physics trigger menu and rates

**Fig. 48:**
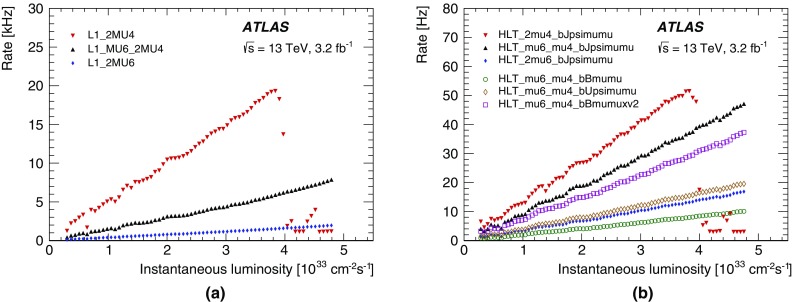
Trigger rates for **a** low-$p_{\text{T}}$ dimuon L1 triggers with various muon $p_{\text{T}}$ thresholds and **b** primary HLT *B*-physics triggers as a function of instantaneous luminosity. **b** Shows triggers requiring two muons to pass various $p_{\text{T}}$ thresholds, to have an invariant mass within the $J/\psi$ mass window, and to form a good vertex (*full markers*); also shown are triggers requiring two muons with $p_{\text{T}} > 6$ and 4 $\text{GeV}$ and either having an invariant mass in a different window ($B^0_{(s)}$, $\Upsilon (1,2,3\mathrm{S})$) or forming a $B\rightarrow \mu \mu X$ candidate after combination with additional tracks found in ID (*open markers*). As L1_2MU4 was prescaled at luminosities above $4\times 10^{33}\,{\text{cm}}^{-2}{\text{s}}^{-1}$, the rate of 2mu4_bJpsimumu seeded from this L1 trigger drops above that luminosity

Dimuon trigger rate restrictions at L1 define the lowest muon transverse momentum thresholds for primary *B*-physics triggers in 2015 data-taking. HLT triggers using L1_2MU4 were unprescaled up to a luminosity of $4\times 10^{33}$ cm$^{-2}$ s$^{-1}$. Above this, triggers seeded from L1_MU6_2MU4,^6^ which requires two muons with $p_{\text{T}}$ above 4 and 6 $\text{GeV}$, were unprescaled. The overall loss of events collected with the former amounts to 15%. Higher-threshold triggers seeded from L1_2MU6 and L1_2MU10 were also active. Figure 48 shows the L1 rates for low-$p_{\text{T}}$ dimuon triggers as well as the HLT rates for various primary triggers seeded from them, as a function of the instantaneous luminosity.

**Fig. 49:**
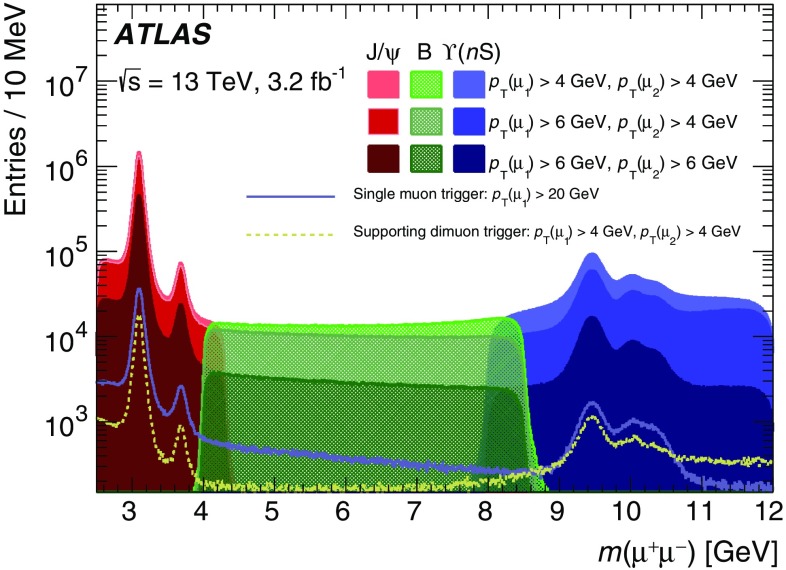
Invariant mass distribution of offline-selected dimuon candidates passing the lowest thresholds of dimuon *B*-physics triggers. Triggers targeting different invariant mass ranges are illustrated with different colours, and the differing thresholds are shown with different shadings. No accounting for overlaps between triggers is made, and the distributions are shown overlaid, and not stacked. For comparison, the number of candidates passing the lowest unprescaled single-muon trigger and supporting dimuon trigger is also shown

The invariant mass distribution of offline reconstructed dimuon candidates passing the suite of primary triggers is shown in Fig. 49. For comparison, the number of candidates passing the lowest unprescaled single-muon trigger is also shown, as well as the supporting dimuon trigger with wide invariant mass range.

#### *B*-physics trigger efficiencies

To evaluate the efficiency of the *B*-physics selection at the HLT, two supporting triggers with and without the opposite-sign and vertex criteria are used. The first trigger requires that the events contain two opposite-sign muons and form a good fit to a common vertex, using the ID track parameters of the identified muons with a $\chi^{2} < 20$ for the one degree-of-freedom. This selection is the same as used in primary dimuon triggers but has a wider invariant mass window. The second trigger differs by the absence of the muon charge selection and vertex fit. The efficiency is calculated using a sample collected by these triggers.

**Fig. 50:**
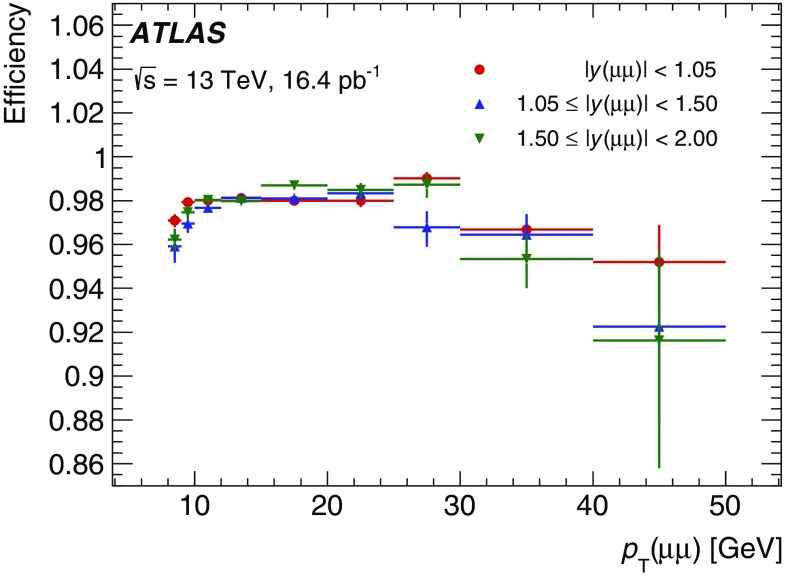
The efficiency of the opposite-sign muon requirement and vertex quality selection applied for dimuon *B*-physics triggers as a function of $p_{\text{T}} (\mu \mu )$ for three rapidity regions. Supporting dimuon triggers with and without the selection criteria applied are used to determine the efficiency. The integrated luminosity shown takes into account the high prescale factors applied to the supporting triggers

For the efficiency measurement, events are selected by requiring two offline reconstructed combined muons satisfying the *tight* quality selection criteria and $p_{\text{T}} (\mu ) > {4}\,{\text{GeV}}$, $|\eta (\mu )|<2.3$. The offline muons are fit to a common vertex, using their ID track parameters, with a fit quality of $\chi^2/\mathrm{dof}<10$ and invariant mass $|m(\mu \mu ) - m_{J/\psi }| < {0.3}\,{\text{GeV}}$. The number of $J/\psi$ candidates is determined from a fit to the offline dimuon invariant mass distribution. The efficiency of the opposite-sign muon requirement and vertex quality selection is shown in Fig. 50 as a function of the offline dimuon transverse momentum $p_{\text{T}} (\mu \mu )$ calculated using the track parameters extracted after the vertex fit, for three slices of $J/\psi$ rapidity. The observed small drop in efficiency at high $p_{\text{T}} (\mu \mu )$ is due to the increasing collinearity of the two muons.

## Conclusion

A large number of trigger upgrades and developments for the ATLAS experiment were made during the first long shutdown of the LHC in preparation for the Run 2 data-taking. A summary of the various updates as well as the first Run 2 performance studies can be found in this paper.

Many improvements in the L1 trigger were implemented including the addition of completely new systems. Upgrades in the L1 calorimeter trigger included the implementation of a dynamic pedestal correction to mitigate pile-up effects. In the L1 muon trigger, a new coincidence logic between the muon end-cap trigger and the innermost muon chamber has been used since 2015, and it is being extended with the hadronic calorimeter, to suppress the fake-muon rate. New chambers were also installed to increase the trigger coverage. In addition, the new central trigger processor doubles the number of L1 trigger thresholds and the L1 output rate limit has increased from 70 to 100 kHz. Furthermore, a new topological processor was installed and is being commissioned. A new HLT architecture was developed to unify the Level-2 and Event Filter scheme used in Run 1, improving the flexibility of the system. The HLT software was also upgraded, making the algorithms and selections closer to the offline reconstruction to maximise the efficiency, and making use of the newly installed systems such as the innermost pixel layer IBL.

The trigger menu was revisited and redesigned to cope with the greater rates due to the higher centre-of-mass energy and increasing instantaneous luminosity. The different trigger signatures were set up according to the physics needs, considering different luminosity scenarios. The ATLAS trigger system was successfully commissioned with the first data acquired at 13 $\text{TeV}$. First performance studies of the different trigger signatures and trigger efficiencies with respect to the offline quantities are presented using the 13 TeV proton–proton collision data with a 25 ns bunch separation collected during 2015.

## Footnotes

1 ATLAS uses a right-handed coordinate system with its origin at the nominal interaction point (IP) in the centre of the detector and the *z*-axis along the beam pipe. The *x*-axis points from the IP to the centre of the LHC ring, and the *y*-axis points upward. Cylindrical coordinates $(r,\phi )$ are used in the transverse plane, $\phi$ being the azimuthal angle around the *z*-axis. The pseudorapidity is defined in terms of the polar angle $\theta$ as $\eta =-\ln \tan (\theta /2)$.

2 The four complex dead-time settings were 15/370, 42/381, 9/351 and 7/350, where the first number specifies the number of triggers and the second number specifies the number of bunch-crossings, e.g. 7 triggers in 350 bunch-crossings.

3
$R_{\text{had}} = E^{\text{had}}_{{\mathrm{T}}} / E^{\text{EM}}_{{\mathrm{T}}}$ is the ratio of the cluster transverse energy in the hadronic calorimeter to that in the EM calorimeter. $R_\eta$ is based on the cluster shape in the second layer of the EM calorimeter and defined as the ratio of transverse energy in a core region of $3\times 7$ cells in $\eta \times \phi$ to that in a $7\times 7$ region, expanded in $\eta$ from the $3\times 7$ core. $E_{\text{ratio}}$ is defined as the ratio of the energy difference between the largest and second-largest energy deposits in the cluster over the sum of these energies in the front layer of the EM calorimeter.

4 A one-sided $2\sigma$ noise cut was used during Run 1, which resulted in a bias towards higher $E_{\text{T}}^{\text{miss}}$-values.

5 A $\Sigma E_{\text{T}}$ trigger was used during heavy-ion collisions at L1.

6 L1 muon thresholds are inclusive, i.e. L1_MU6_2MU4 is a dimuon trigger.
